# Preparation, characterization, and catalytic performance of a 2,3-dihydroxybenzoic acid-based deep eutectic solvent for the synthesis of chromenoquinolinones

**DOI:** 10.1039/d6ra07325e

**Published:** 2026-09-17

**Authors:** Hadis Goudarzi, Davood Habibi, Abdolhamid Alizadeh, Arezo Monem

**Affiliations:** a Department of Organic Chemistry, Faculty of Chemistry and Petroleum Chemistry, Bu-Ali Sina University Hamedan 6517838683 Iran davood.habibi@gmail.com dhabibi@basu.ac.ir +98 81 31408025 +98 81 31406070; b Department of Organic Chemistry, Faculty of Chemistry, Al-Zahra University Tehran 1993893973 Iran

## Abstract

An ethyltriphenylphosphonium bromide/2,3-dihydroxybenzoic acid deep eutectic solvent (ETPPBr/2,3-DHBA-DES, 1 : 2 molar ratio) was prepared and characterized by eutectic phase behavior analysis, FT-IR spectroscopy, ^1^H NMR spectroscopy, TGA/DTA, and density measurements, confirming the formation of a eutectic system. The DES was then employed as a recyclable catalyst for the one-pot three-component synthesis of 6*H*-benzo[*h*]chromeno[4,3-*b*]quinolin-6-ones *via* the condensation of 4-hydroxycoumarin, 1-naphthylamine, and aryl or heteroaryl aldehydes under solvent-free conditions. Under the optimized reaction conditions (0.50 mmol catalyst, 80 °C), ten target derivatives were obtained in 60.8–85.0% isolated yields within 45–90 min. The catalyst was readily recovered by aqueous extraction and reused for four consecutive cycles, affording product yields from 85% in the first run to 72% in the fourth. FT-IR analysis of the recovered catalyst indicated that its characteristic structural features were largely preserved after recycling. These results demonstrate that the ETPPBr/2,3-DHBA-DES is an effective and recyclable catalyst for the solvent-free synthesis of 6*H*-benzo[*h*]chromeno [4,3-*b*]quinolin-6-ones.

## Abstract 2

An ETPPBr/2,3-DHBA-DES, 1 : 2 molar ratio) was prepared and characterized by eutectic phase behavior analysis, FT-IR spectroscopy, ^1^H NMR spectroscopy, TGA/DTA, and density measurements, confirming the formation of a eutectic system.

## Graphical abstract

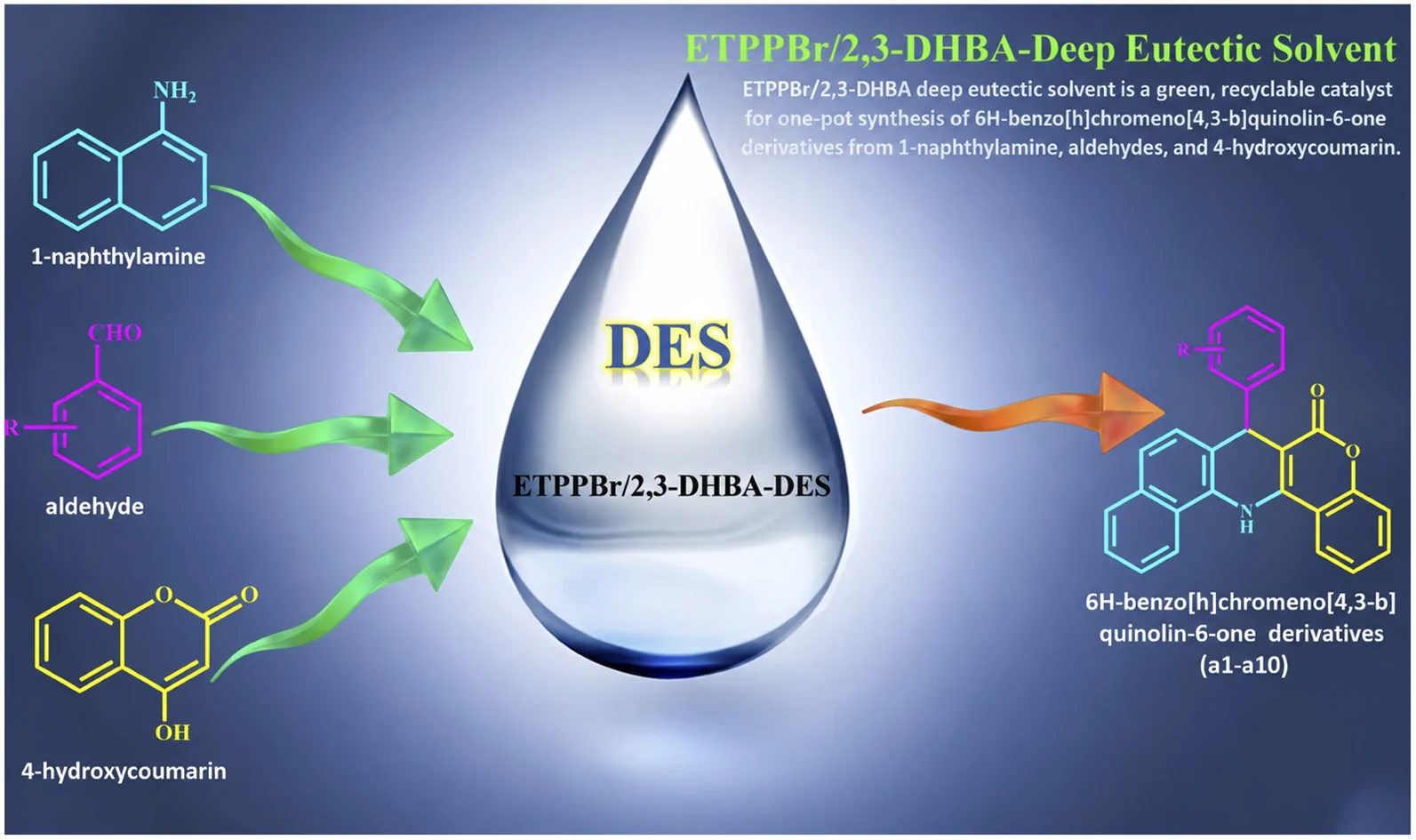

## 1. Introduction

Green chemistry emerged during the 1990s with the primary goal of reducing the use and environmental impact of toxic, volatile, and corrosive solvents in response to growing environmental concerns.^1,2^ Consequently, considerable research effort has been devoted to the development of suitable alternative solvents, including ionic liquids, liquid polymers, and supercritical fluids.^3,4^

Deep eutectic solvents (DESs) are a class of green solvents that have attracted considerable attention due to their cost-effectiveness, ease of preparation, short preparation time, minimal waste generation, structural diversity, and biodegradability. These solvents are formed through the interaction between a hydrogen bond donor (HBD) and a hydrogen bond acceptor (HBA), resulting in a melting point significantly lower than those of the individual components.^5^ Since their discovery, DESs have found numerous applications in various fields, mainly because of their unique solvent properties and environmentally friendly nature, which enable them to dissolve a wide range of substances. Their applications include metal extraction and recovery from solutions,^6,7^ ore refining,^8^ use as solvents and catalysts in chemical reactions,^9–13^ pharmaceutical solvents,^14–18^ oxidative desulfurization,^19,20^ natural gas sweetening and CO_2_ removal,^21,22^ and the extraction of phenolic compounds from orange peel and Juglans.^23,24^

In recent years, deep eutectic solvents have emerged as attractive media for the green synthesis of heterocyclic compounds, particularly through multicomponent reactions (MCRs). Besides serving as environmentally benign solvents, DESs can also act as catalysts by promoting hydrogen-bond interactions that activate carbonyl compounds and reaction intermediates, thereby facilitating bond formation under mild conditions. Their dual role as solvent and catalyst, together with their recyclability, operational simplicity, and compatibility with solvent-free protocols, makes DESs highly suitable for the sustainable synthesis of structurally diverse heterocyclic scaffolds.^25–27^

The progress and development of green chemistry in recent years have had a significant impact on the synthesis of various heterocyclic compounds. Chromenes are an important class of heterocyclic compounds consisting of a benzene ring fused to a pyran ring.^28^ Quinolines are also important structural motifs found in numerous natural products and synthetic compounds and exhibit a broad range of pharmacological activities.^29^ Accordingly, 6*H*-chromeno[4,3-*b*]quinolines, which combine the structural features of chromene and quinoline moieties, are of considerable pharmaceutical interest, for example, as β-selective ligands for the estrogen receptor.^30^

Several classical chromeno-quinoline fused core compounds have promising pharmaco-logical activity. For example, 9,10-dimethoxy-6*H*-chromeno[3,4-*b*]quinoline and 2-fluoro-9, 10-dimethoxy-6*H*-chromeno[3,4-*b*]quinoline act as anticancer compounds (DNA topoisomer-ase-I inhibitor), 7-chloro-3,9-dihydroxy-6*H*-chromeno[4,3-*b*]quinoline as a selective estrogen-receptor-β ligand and 6*H*-chromeno[4,3-*b*]quinoline as a parent medicinal-chemistry scaffold, rather than a medicine.^31^ Mulakayala and coworkers synthesized a series of these chromeno–quinolinone compounds and evaluated them against human cancer cells. Several showed substantial antiproliferative activity. Mechanistic experiments suggested that sirtuin inhibition could contribute to the activity.^32^ Sultana and coworkers reported 18 compounds based on the 6-phenyl-6*H*-chromeno[4,3-*b*]quinoline scaffold. They tested the library against A549, DU145, PC3, MCF7, HT29 and HCT116 cancer cells. One of them was the most noteworthy reported member, showing approximately IC_50_ = 2.61 ± 0.34 µM against HT29 colon cancer cells. Apoptosis and inhibition of cancer-cell migration were also reported.^33^ In general, chromenoquinolinones possess significant fluorescence, biological, and medicinal properties.^34^

Since the catalytic behavior of deep eutectic solvents strongly depends on the nature of both the hydrogen-bond donor (HBD) and the hydrogen-bond acceptor (HBA), the development of new DES systems and the evaluation of their catalytic performance in different organic transformations remain active areas of research. In our previous work, we reported a phosphonium-based deep eutectic solvent containing 3,4-dihydroxybenzoic acid as an efficient catalyst for the synthesis of multi-substituted imidazoles. Building upon this work, the present study explores a new phosphonium-based deep eutectic solvent by employing the positional isomer 2,3-dihydroxybenzoic acid as the hydrogen-bond donor and evaluates its catalytic performance in the solvent-free synthesis of benzo[*h*]chromeno[4,3-*b*]quinolines.^35^

**Fig. 1:**
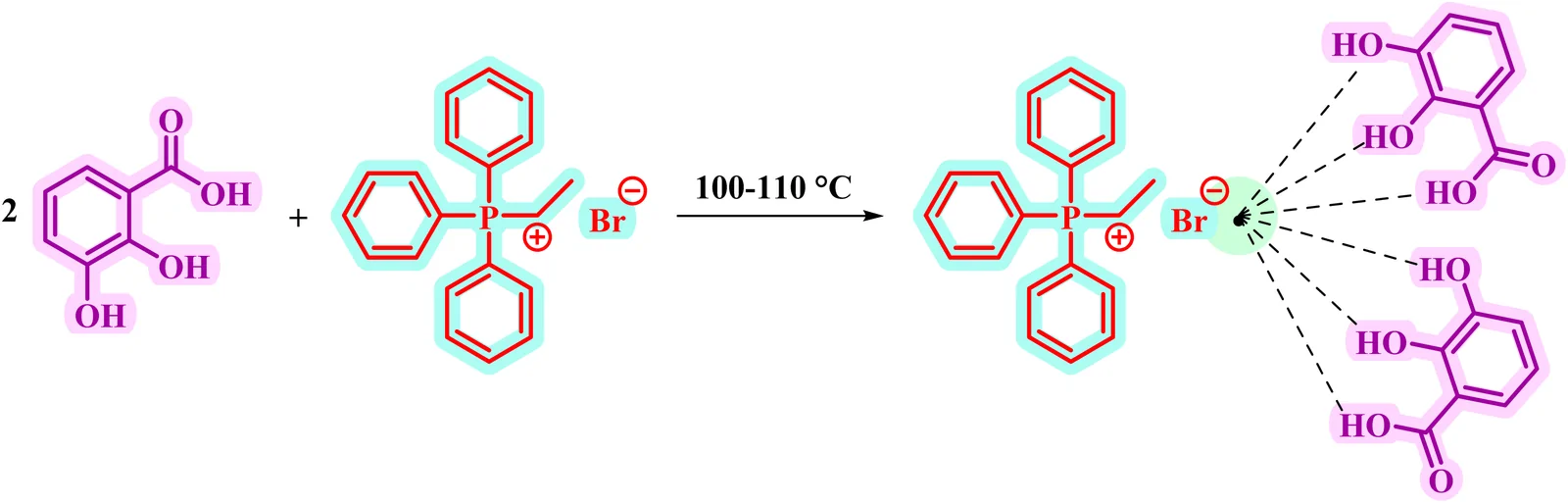
Schematic preparation of ETPPBr/2,3-DHBA-DES.

In the present study, a new deep eutectic solvent (ETPPBr/2,3-DHBA-DES) was prepared from ethyltriphenylphosphonium bromide (ETPPBr) and 2,3-dihydroxybenzoic acid (2,3-DHBA) at the optimum molar ratio determined from the eutectic phase diagram. The resulting DES was characterized by FT-IR, TGA/DTA, and ^1^H NMR spectroscopy, together with density measurements (Fig. 1).^36–38^

**Fig. 2:**
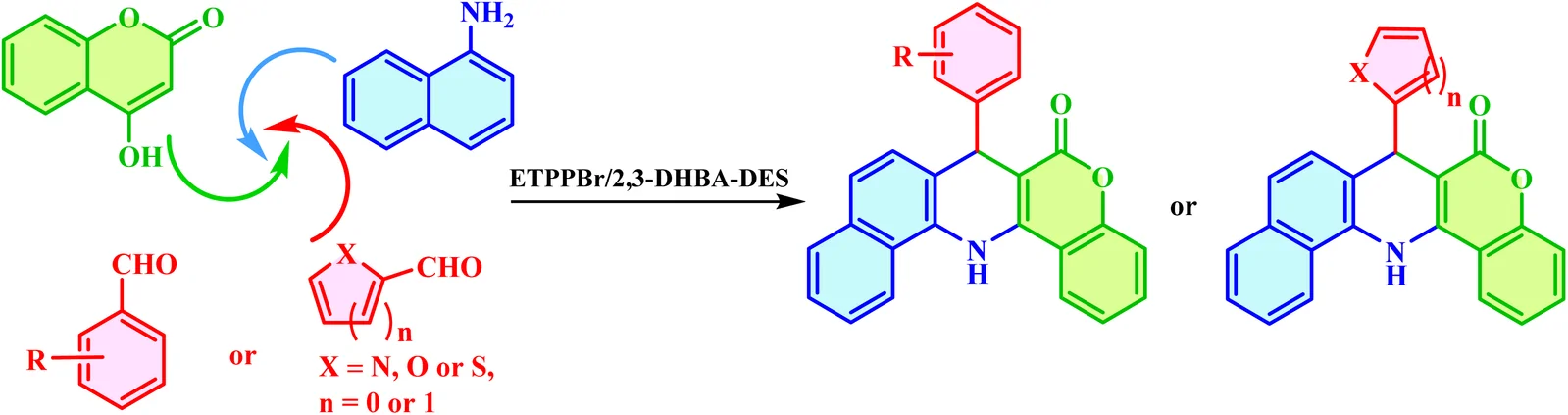
Synthetic route towards a(1–10).

Then, the catalytic performance of the new DES was evaluated in the solvent-free one-pot three-component synthesis of 6*H*-benzo[*h*]chromeno[4,3-*b*]quinolin-6-ones a(1–10) (Fig. 2).

## 2. Experimental

### 2.1. Materials

All reagents were purchased from Sigma-Aldrich and Merck and used without further purification. The ^1^H NMR spectrum of ETPPBr was recorded at 400 MHz, while the ^1^H NMR spectra of 2,3-DHBA and the prepared DES were recorded at 500 MHz. The synthesized products were characterized by ^1^H and ^13^C NMR spectroscopy at 250 and 62.5 MHz, respectively using DMSO-*d*_6_ as the solvent. FT-IR spectra were recorded using KBr pellets on a PerkinElmer FT-IR spectrophotometer. Analytical thin-layer chromatography (TLC) was performed on pre-coated silica gel 60 F254 TLC plates (Merck, Darmstadt, Germany). Melting points were determined in open capillary tubes using a Stuart melting point apparatus and are uncorrected. Density measurements were performed using an AND HR-200 densitometer.

### 2.2. Density determination of ETPPBr/2,3-DHBA-DES

The density of ETPPBr/2,3-DHBA-DES was determined using an and HR-200 density determination kit based on the Archimedes principle. The density was calculated according to the equation *ρ* = *ρ*_w_/1−*M*_w_ ÷ *M*_d_, where *ρ* is the density of the sample, *ρ*_w_ is the density of water, *M*_w_ is the mass of the wet sample, and *M*_d_ is the mass of the dry sample.

### 2.3. General procedure for preparation of ETPPBr/2,3-DHBA-DES

**Fig. 3:**
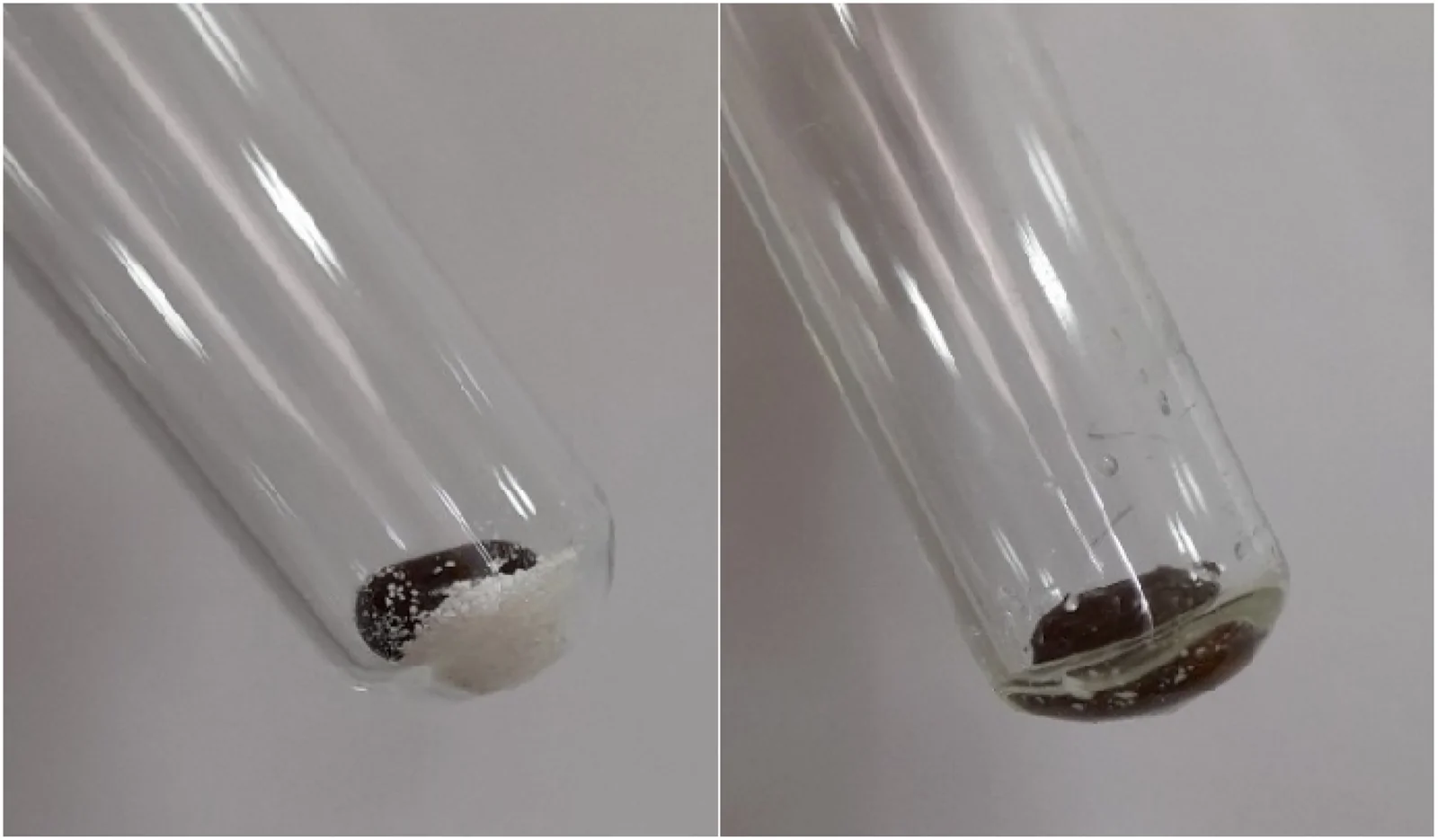
Physical appearance of the ETPPBr/2,3-DHBA-DES system before (left) and after heating (right). Formation of a homogeneous transparent deep eutectic solvent was observed after heating. The dark-colored object visible at the bottom of the tube is the stirring magnet, not the solid phase of the DES.

ETPPBr and 2,3-DHBA were used as received without additional drying. ETPPBr (0.185 g, 0.50 mmol) and 2,3-DHBA (0.154 g, 1.00 mmol) were mixed at a molar ratio of 1 : 2 and heated at 100–110 °C for approximately 30 min with continuous mixing until a homogeneous and transparent phase was obtained. The resulting mixture was then allowed to cool to room temperature, where it formed a transparent, highly viscous and essentially non-flowing phase. Upon reheating, the sample was heated gradually, and the temperature was monitored using a thermometer. The temperature required for complete fluidity was determined visually as the temperature at which the sample became completely fluid and readily flowable. For the 1 : 2 ETPPBr/2,3-DHBA composition, complete fluidity was observed at approximately 70 °C, and the material was subsequently used as the catalyst in the following reactions. The DES was freshly prepared immediately before each catalytic experiment and used without further drying. A separately prepared DES sample was stored at room temperature for several weeks to evaluate its physical stability. No visible crystallization or phase separation was observed during this period (Fig. 3).

### 2.4. General procedure for preparation of a(1–10)

A mixture of aldehyde (1.0 mmol), 1-naphthylamine (1.0 mmol), 4-hydroxycoumarin (1.0 mmol), and ETPPBr/2,3-DHBA-DES (0.50 mmol) was stirred at 80 °C under solvent-free conditions until completion of the reaction (45–90 min, monitored by TLC). After cooling to room temperature, the solid product was isolated by filtration. Compound a1 was washed with ethanol and dried, whereas the remaining derivatives a(2–10) were purified by recrystallization from DMF.

For recovery of the DES, water and chloroform (or ethyl acetate) were added to the remaining reaction mixture. The DES was transferred into the aqueous phase, whereas the organic impurities remained in the chloroform (or ethyl acetate) layer. The aqueous phase was separated, and water was removed under reduced pressure to afford the recovered DES, which was reused in subsequent catalytic runs.

## 3. Results and discussion

### 3.1. Characterization of ETPPBr/2,3-DHBA-DES

The prepared ETPPBr/2,3-DHBA-DES was characterized using FT-IR spectroscopy, ^1^H NMR spectroscopy, TGA/DTA, density measurements, and eutectic phase diagram analysis to evaluate its structural and physicochemical properties. The results obtained from these characterization techniques are discussed below.

### 3.2. Eutectic point phase diagram of ETPPBr/2,3-DHBA-DES

**Fig. 4:**
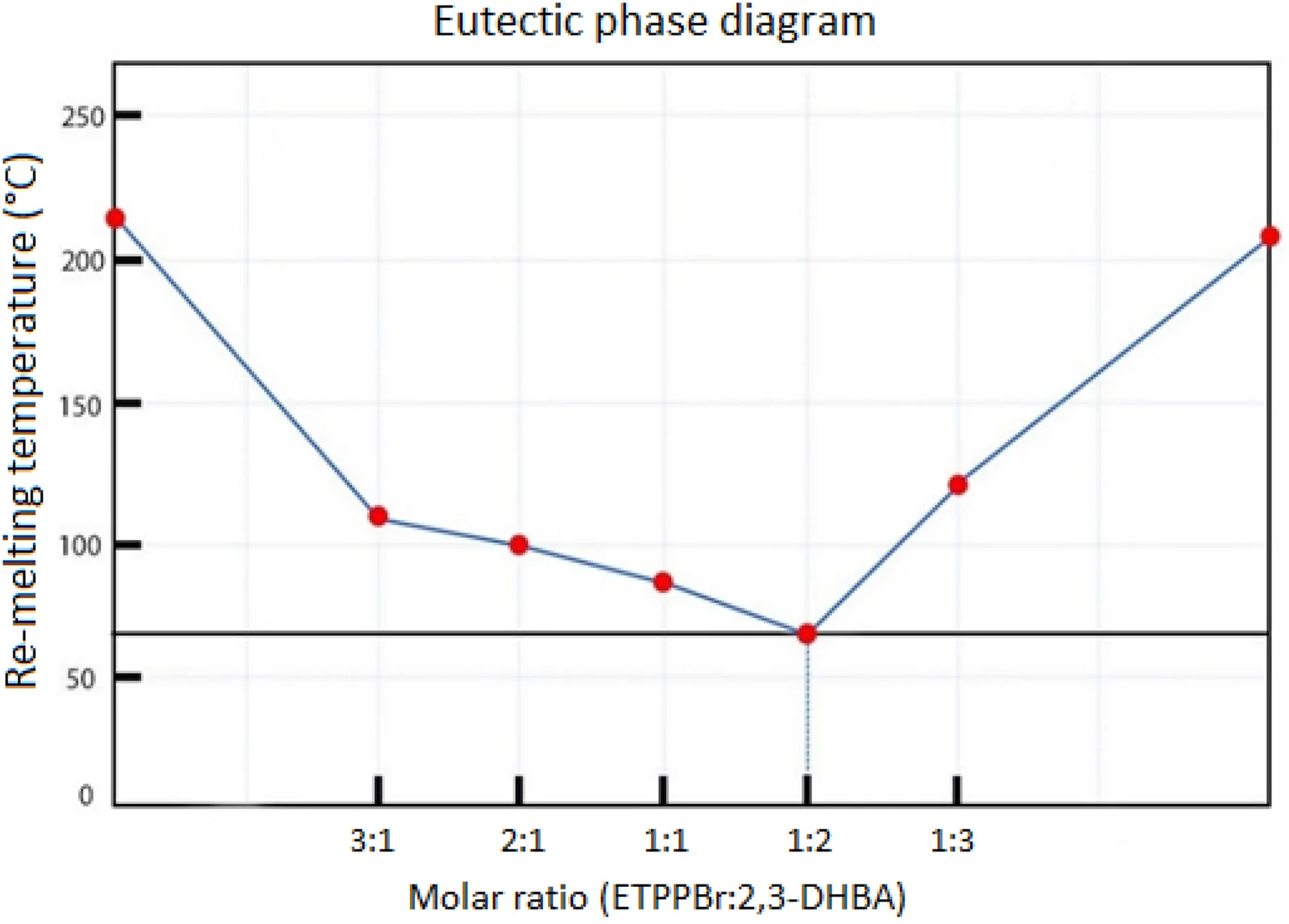
Eutectic point phase diagram of the ETPPBr/2,3-DHBA-DES system. The minimum re-melting temperature (70 °C) was observed at an ETPPBr : 2,3-DHBA molar ratio of 1 : 2, corresponding to the eutectic composition.

To determine the optimum eutectic composition, different molar ratios of ETPPBr and 2,3-DHBA were prepared. After the initial preparation, each sample was cooled to room temperature and subsequently reheated. The temperature at which the sample became completely transparent and a freely flowing liquid was recorded, and based on this, the eutectic phase diagram was plotted. As shown in Fig. 4, the molar ratio of 1 : 2 (ETPPBr : 2,3-DHBA) exhibited the lowest temperature for achieving complete transparency and fluidity (70 °C) and was therefore selected as the optimum eutectic composition for the preparation of ETPPBr/2,3-DHBA-DES and subsequent catalytic studies.

### 3.3. FT-IR analysis of ETPPBr/2,3-DHBA-DES

**Fig. 5:**
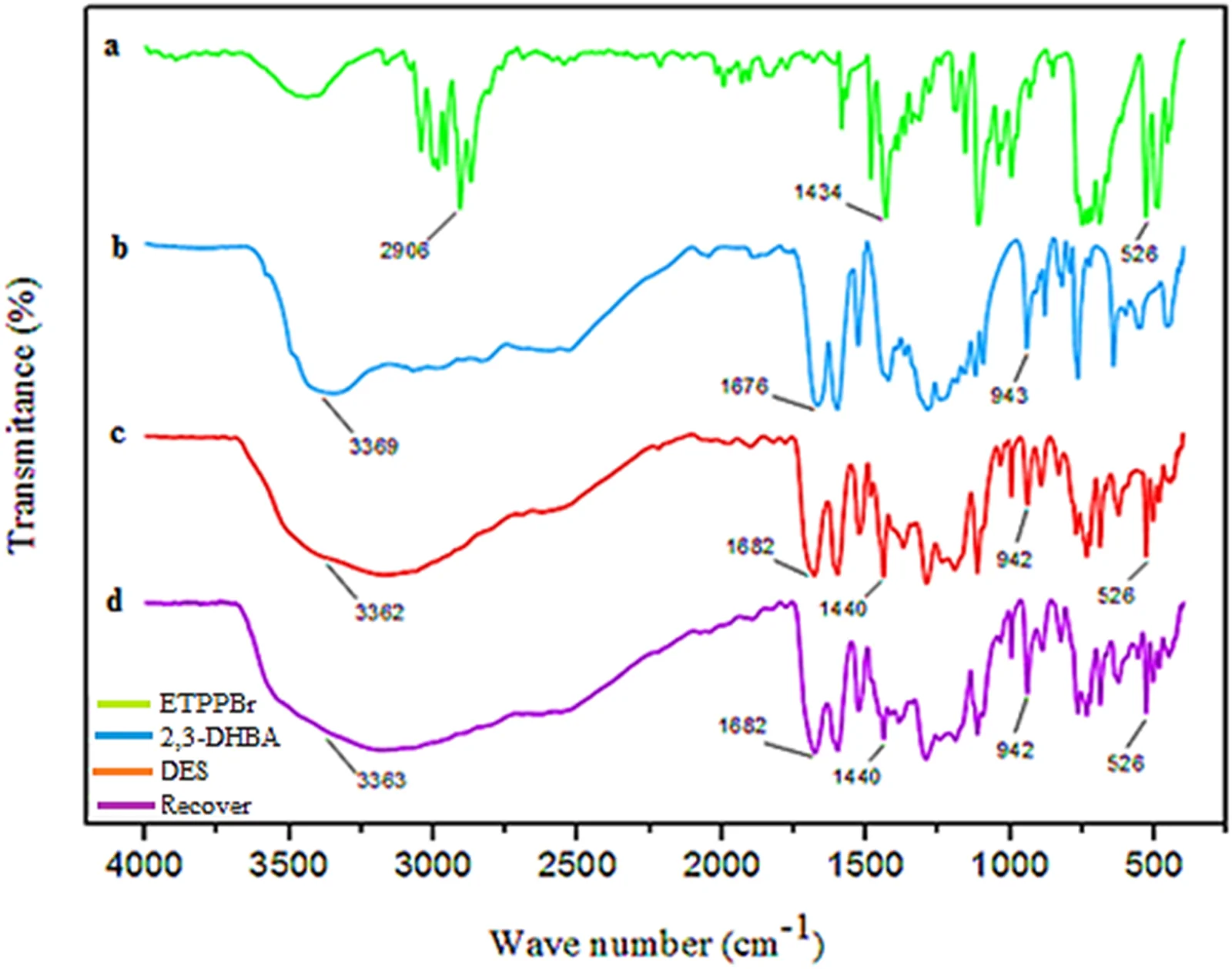
Comparison of the FT-IR spectra of ETPPBr, 2,3-DHBA, freshly prepared ETPPBr/2,3-DHBA-DES, and recovered ETPPBr/2,3-DHBA-DES.

Spectrum (a) corresponds to ethyltriphenylphosphonium bromide, with characteristic bands in the regions of 520, 1430, and 2900–3100 cm^−1^, which can be attributed to the C–P stretching vibrations, aromatic ring skeletal vibrations, and aliphatic and aromatic C–H stretching vibrations, respectively. Spectrum (b) corresponds to 2,3-dihydroxybenzoic acid, showing characteristic bands in the 1200–1300, 1670, and broad 3300 cm^−1^ regions, associated with phenolic C–O stretching, carbonyl (C=O) stretching, and O–H stretching vibrations, respectively. All characteristic bands of the starting materials are clearly retained in the DES spectrum (c), while their slight shifts can be attributed to hydrogen-bonding interactions between the components during DES formation. Spectrum (d), corresponding to the recovered DES, shows that the characteristic spectral features are largely preserved, with no significant changes observed, indicating the structural stability of the DES after recovery (Fig. 5).

### 3.4. TGA-DTA analysis of ETPPBr/2,3-DHBA-DES

**Fig. 6:**
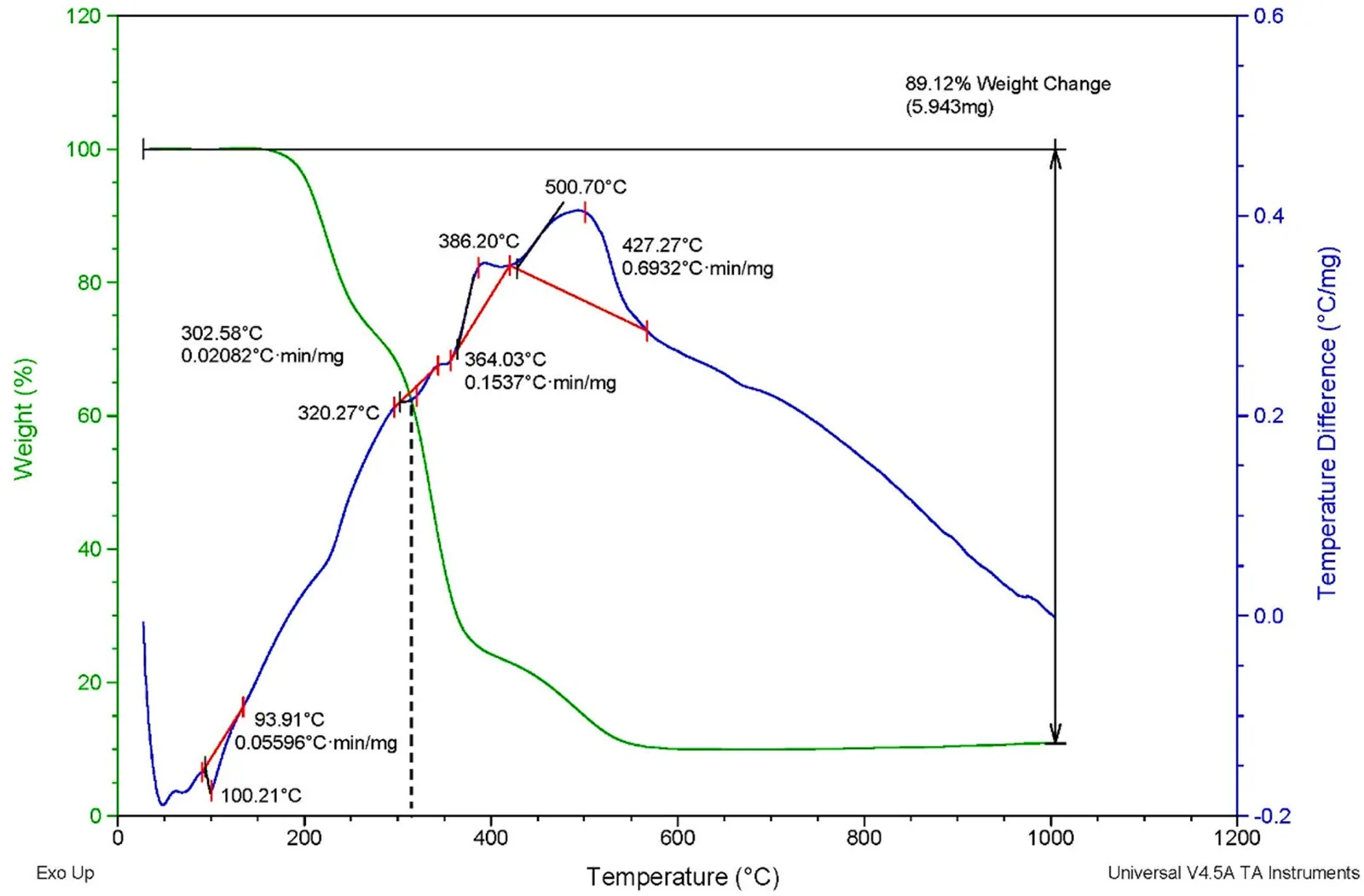
TGA-DTA curves of ETPPBr/2,3-DHBA-DES.

The TGA/DTA curves of ETPPBr/2,3-DHBA-DES indicate a multistep thermal decomposition process (Fig. 6). A very small weight loss below approximately 150 °C is attributed to the removal of physically adsorbed moisture. The main decomposition begins above approximately 320 °C and proceeds through successive degradation steps, which can be assigned to disruption of the hydrogen-bonding network, decomposition of the 2,3-dihydroxy benzoic acid component, and subsequent degradation of the phosphonium salt. An overall weight loss of approximately 89% was observed up to 1000 °C, leaving a small residual mass that is likely associated with phosphorus-containing and carbonaceous residues. These results demonstrate that ETPPBr/2,3-DHBA-DES possesses sufficient thermal stability for the present catalytic reactions, which were performed at only 80 °C.

### 3.5. ^1^H NMR analysis of ETPPBr/2,3-DHBA-DES

**Fig. 7:**
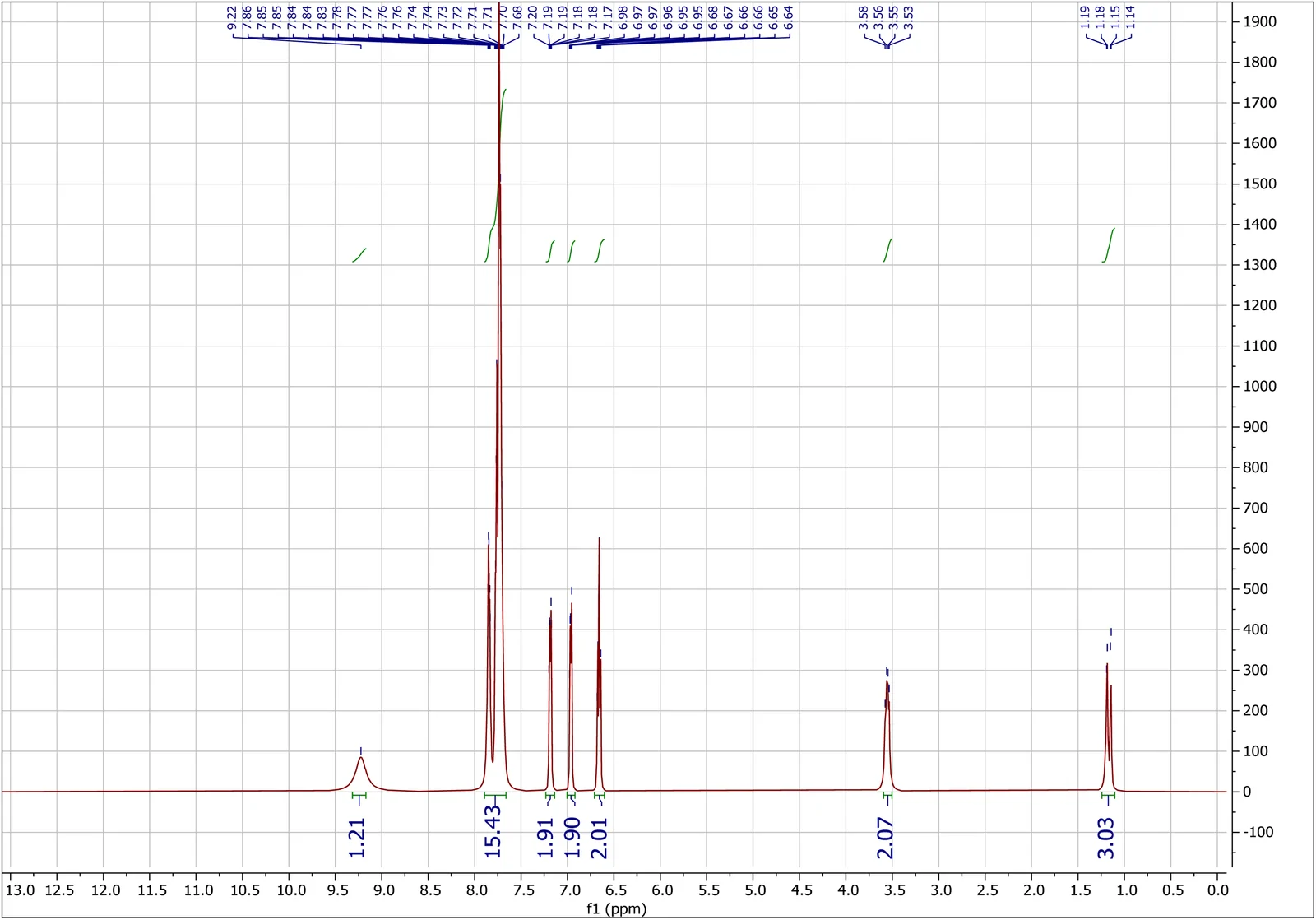
^1^H NMR spectrum of the freshly prepared ETPPBr/2,3-DHBA-DES.

The ^1^H NMR spectrum of the freshly prepared ETPPBr/2,3-DHBA-DES is presented in Fig. 7. The characteristic proton signals of both ETPPBr and 2,3-dihydroxybenzoic acid (2,3-DHBA) were observed in the DES spectrum, indicating that the structural features of both components were retained after DES formation.

**Fig. 8:**
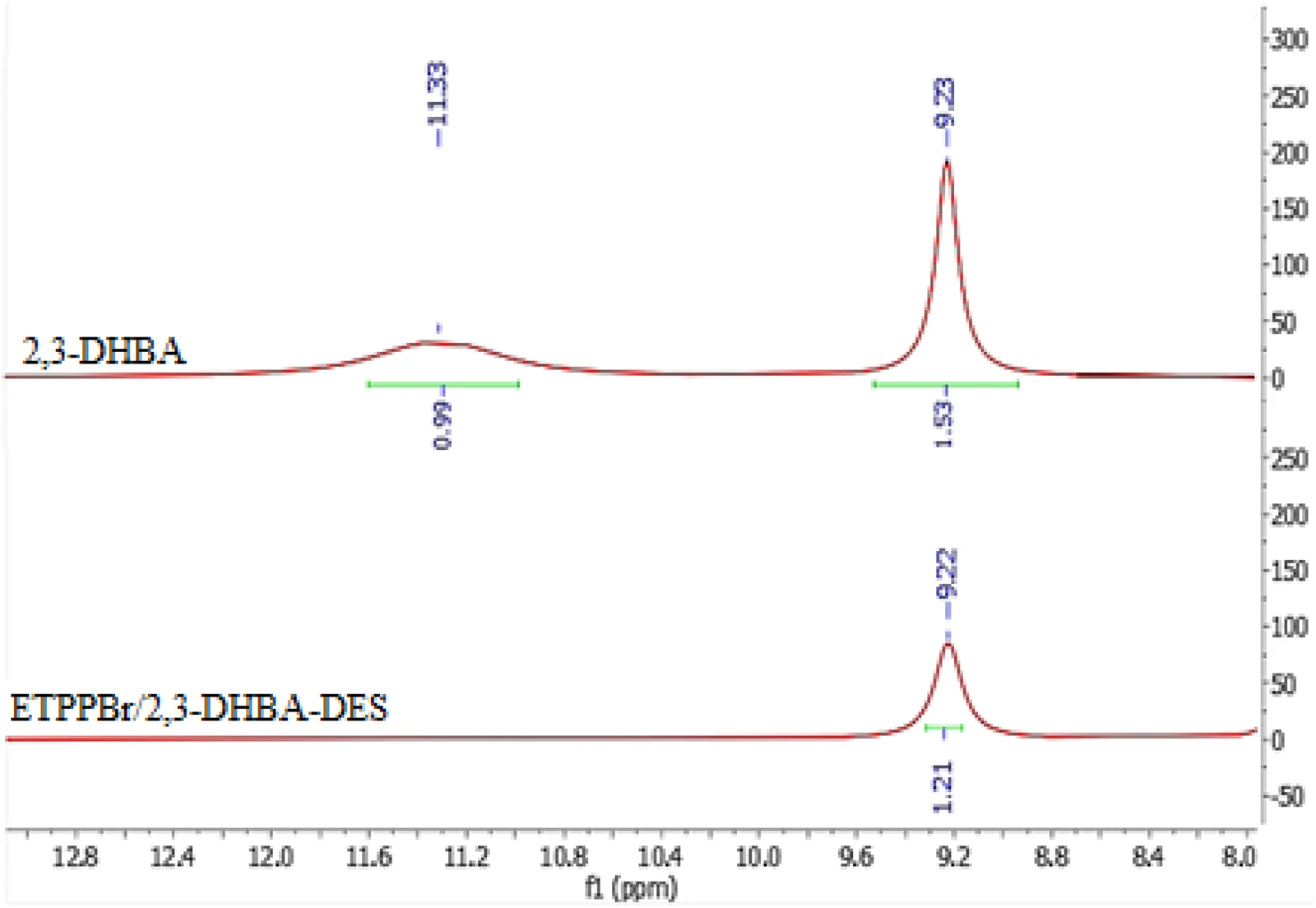
Comparison of the expanded ^1^H NMR spectra in the carboxylic acid proton region of 2,3-DHBA and ETPPBr/2,3-DHBA-DES.

To further investigate the interactions between the HBA and HBD, the carboxylic acid proton region of DES was compared with that of pure 2,3-DHBA (Fig. 8). A decrease in the integral intensity of the carboxylic acid proton together with slight changes in the corresponding resonance was observed after DES formation, suggesting the establishment of intermolecular hydrogen-bonding interactions between ETPPBr and 2,3-DHBA. These observations support the formation of the ETPPBr/2,3-DHBA-DES.

The complete ^1^H NMR spectra of the starting materials are provided in the SI.^33^

### 3.6. Density measurement of ETPPBr/2,3-DHBA-DES

The density of ETPPBr/2,3-DHBA-DES was determined using the Archimedes method and found to be 1.286 g mL^−1^. This value lies within the typical density range reported for deep eutectic solvents (1.0–1.6 g mL^−1^), indicating that the prepared DES exhibits physicochemical properties comparable to those of previously reported DES systems.

### 3.7. Optimization of the model reaction conditions

The reaction conditions for the synthesis of 7-(3-hydroxyphenyl)-7,14-dihydro-6*H*-benzo[*h*] chromeno[4,3-*b*]quinolin-6-one (a1, model reaction) were optimized by investigating the effects of catalyst loading, reaction temperature, and solvent. The model reaction was carried out using 3-hydroxybenzaldehyde, 1-naphthylamine, and 4-hydroxycoumarin in a 1 : 1 : 1 molar ratio. The results are summarized in Tables 1–3.

The effect of catalyst loading was first investigated under solvent-free conditions at 80 °C for 1 h (Table 1). Among the examined catalyst loadings, 0.50 mmol of ETPPBr/2,3-DHBA-DES afforded the highest isolated yield (85%) and was therefore selected as the optimum catalyst loading for subsequent experiments. The decrease in product yield at higher DES loadings may be attributed to the increased viscosity of the reaction medium, which can hinder effective mixing and mass transfer. The increased amount of 2,3-DHBA introduced with the DES may also alter the reaction environment and reduce the effective catalytic efficiency. Furthermore, increasing the amount of catalyst may accelerate the reverse reaction, thereby reducing the reaction yield.

**Table 1:** Effect of catalyst loading on the model reaction (a1) under solvent-free conditions at 80 °C for 1 h

Entry	DES (mmol)^a^	Yield (%)
1	1.50 = 1.019 g	70
2	1.00 = 0.679 g	78
3	0.50 = 0.340 g	85
4	0.25 = 0.170 g	60

a 1 mmol DES = 1 mmol ETPPBr + 2 mmol 2,3-DHBA = 0.679 g.

The effect of reaction temperature was investigated using the optimized catalyst loading (0.50 mmol) under solvent-free conditions for 1 h (Table 2). The highest product yield (85%) was obtained at 80 °C. Both lower and higher temperatures resulted in decreased product yields. Therefore, 80 °C was selected as the optimum reaction temperature for subsequent experiments.

**Table 2:** Effect of reaction temperature on the model reaction (a1) under solvent-free conditions using 0.50 mmol of ETPPBr/2,3-DHBA-DES

Entry	Temp. (°C)	Yield (%)
1	60	52
2	80	85
3	100	70
4	120	74

The effect of solvent was investigated using the optimized catalyst loading (0.50 mmol) under different reaction media for 1 h (Table 3). Among the solvents examined, the highest product yield (85%) was obtained under solvent-free conditions at 80 °C. Reactions carried out in organic solvents under reflux afforded lower yields, while no product was obtained in water. Therefore, solvent-free conditions were selected as the optimum reaction medium for subsequent experiments.

**Table 3:** Effect of solvent on the model reaction (a1) using 0.50 mmol of ETPPBr/2,3-DHBA-DES

Entry	Reaction conditions	Solvent	Yield (%)
1	Reflux	Ethanol	69
2	Reflux	Ethyl acetate	43
3	Reflux	Water	—
4	Reflux	*n*-Hexane	10
5	80 °C	Solvent free	85

In addition, three appropriate control experiments were conducted under similar reaction conditions, as follows:

1 The synthesis of a1 without the use of ETPPBr/2,3-DHBA (catalyst free).

2 The synthesis of a1 with the use of ETPPBr alone, and.

3 The synthesis of a1 with the use of 2,3-DHBA alone.

In the catalyst-free reaction, the yield was about 28%. In the absence of a DES catalyst, one would ostensibly expect the reaction yield to be zero; however, since aldehydes and amines are inherently capable of reacting with one another—and given that 4-hydroxycoumarin possesses sufficient nucleophilic activity-the reaction yield was about 28%.

In the use of ETPPBr alone, the yield was increased to about 33%. Apparently, the phosphonium salt increased the polarity of the reaction medium, thereby enhancing the reaction yield.

In the use of 2,3-DHBA alone, the reaction yield increased to about 91%. When DES is employed, its acidic COOH group and two phenolic OH groups likely interact with the positive and negative moieties of the phosphonium salt; conversely, when 2,3-DHBA is used alone, such interactions do not occur, allowing it to effectively protonate the aldehyde carbonyl group involved in the three-component reaction, thereby facilitating the reaction and increasing the yield.

### 3.8. Synthesis of a(1–10)

Using the optimized reaction conditions, a(1–10) were synthesized *via* the three-component reaction of various aldehydes (benzaldehydes, picolin-aldehyde, or thiophene-2-carbaldehyde), 1-naphthylamine, and 4-hydroxycoumarin in the presence of ETPPBr/2,3-DHBA-DES (0.50 mmol) under solvent-free conditions at 80 °C. The desired products were obtained in good isolated yields within reasonable reaction times, as summarized in Table 4.

**Table 4:** Synthesis of a(1–10) under the optimized reaction conditions

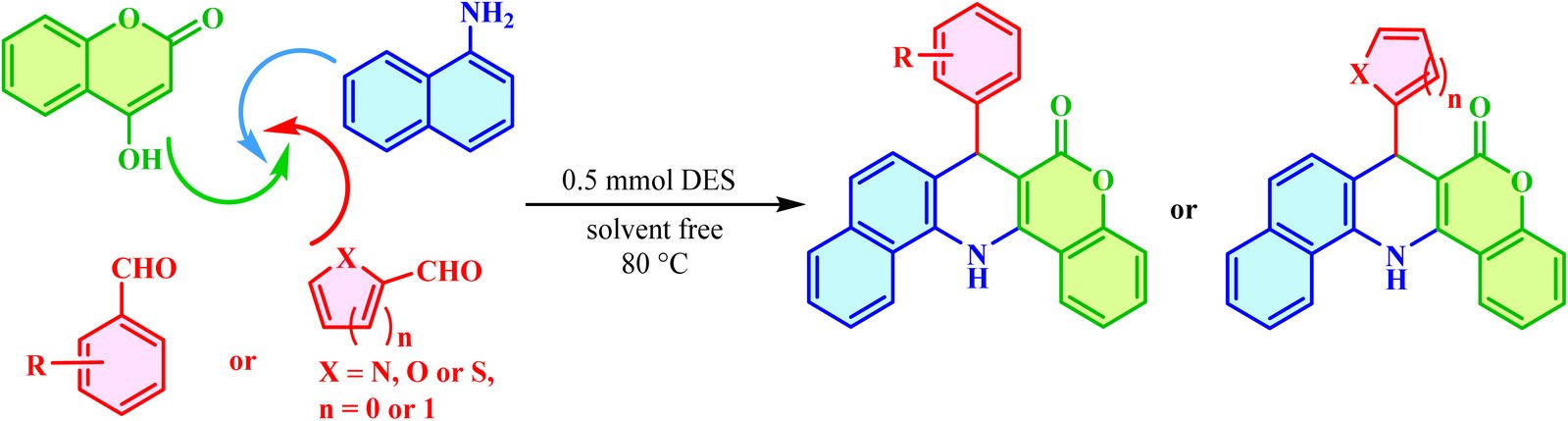					
Entry	Product	Time (min)	Yield (%)	M.P. (°C) found	M.P. (°C) literature^34,35^
1	a1	45	85	>300	337
2	a2	60	82	>300	>300
3	a3	54	72.4	>300	>300
4	a4	58	76.9	>300	>300
5	a5	90	78.7	>300	335
6	a6	67	60.8	285–289	280–283
7	a7	45	83.9	>300	330–333
8	a8	45	85	>300	305–308
9	a9	90	80.5	>300	>300
10	a10	67	66	210	204

### 3.9. Spectral data of a(1–10) (Fig. 9)

#### 3.9.1. 7-(3-Hydroxyphenyl)-7,14-dihydro-6*H*-benzo[*h*]chromeno[4,3-*b*]quinolin-6-one (a1)

**Fig. 9:**
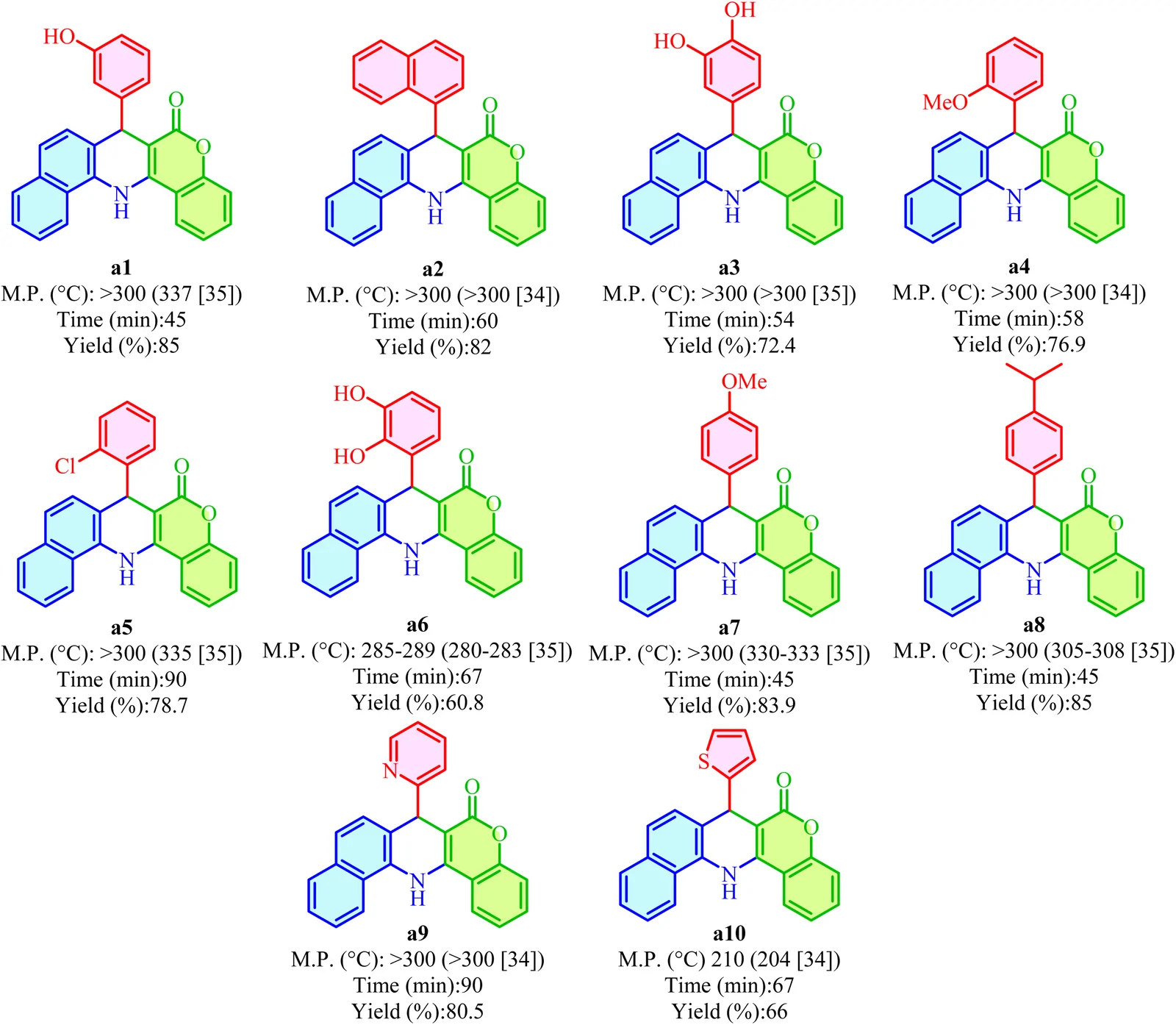
Structures of a(1–10) catalyzed by ETPPBr/2,3-DHBA-DES.

Cream solid, M.p. >300 °C, IR (KBr, cm^−1^): 3440, 3268, 3063, 2900, 1660, 1522, 1471, 1268. ^1^H NMR (250 MHz, DMSO-*d*_6_): *δ* = 9.45 (s, 1H), 9.23 (s, 1H), 8.64 (d, *J* = 8.4 Hz, 1H), 8.56 (d, *J* = 8.1 Hz, 1H), 7.87 (d, *J* = 8.1 Hz, 1H), 7.66–7.59 (m, 2H), 7.52 (d, *J* = 7.4 Hz, 2H), 7.40 (q, *J* = 11.1 Hz, 3H), 7.00 (t, *J* = 7.7 Hz, 1H), 6.75 (d, *J* = 7.4 Hz, 1H), 6.66 (s, 1H), 6.52 (d, *J* = 5.6 Hz, 1H), 5.29 (s, 1H). ^13^C NMR (62.5 MHz, DMSO-*d*_6_) *δ* = 161, 157.9, 152.7, 148.6, 144.3, 133.0, 132.3, 130.5, 129.7, 128.7, 127.8, 126.5, 126.4, 124.4, 124.1, 123.9, 123.2, 122.3, 121, 118.5, 117.2, 114.6, 114.1, 113.9, 99.1, 41.9, EI-MS (70 eV): *m*/*z* 391 [M]^+^˙, 298 (base peak) (Fig. 9).

#### 3.9.2. 7-(Naphthalen-1-yl)-7,14-dihydro-6*H*-benzo[*h*]chromeno[4,3-*b*]quinolin-6-one (a2)

Cream solid, M.p. >300 °C, IR (KBr, cm^−1^): 3360, 3031, 1668, 1618, 1525, 1475, 1265. ^1^H NMR (250 MHz, DMSO-*d*_6_) *δ* = 9.44 (s, 1H), 8.80 (d, *J* = 8.6 Hz, 1H), 8.64 (t, *J* = 10.2 Hz, 2H), 7.86 (d, *J* = 8.1 Hz, 1H), 7.75 (d, *J* = 7.7 Hz, 1H), 7.65 (q, *J* = 7.6 Hz, 4H), 7.51 (d, *J* = 7.5 Hz, 3H), 7.39 (d, *J* = 8.7 Hz, 4H), 7.14 (d, *J* = 8.2 Hz, 1H), 6.26 (s, 1H). ^13^C NMR (62.5 MHz, DMSO-*d*_6_) *δ* = 152.8, 145.5, 144.6, 133.8, 133, 132.3, 130.8, 129.9, 128.6, 126.8, 126.5, 124.5, 124.1, 123.3, 122.4, 121.9, 117.2, 114, 100.1, 37.1, EI-MS (70 eV): *m*/*z* 425 [M]^+^˙, 298 (base peak).

#### 3.9.3. 7-(3,4-Dihydroxyphenyl)-7,14-dihydro-6*H*-benzo[*h*]chromeno[4,3-*b*]quinolin-6-one (a3)

Yellow solid, M.p. >300 °C, IR (KBr, cm^−1^): 3510, 3437, 3025, 2881, 1660, 1524, 1471, 1266. ^1^H NMR (250 MHz, DMSO-*d*_6_) *δ* = 9.43 (s, 1H), 8.71–8.51 (m, 4H), 7.88 (d, *J* = 8.1 Hz, 1H), 7.65–7.53 (m, 4H), 7.45–7.31 (m, 3H), 6.59 (d, *J* = 22.6 Hz, 3H), 5.16 (s, 1H). ^13^C NMR (62.5 MHz, DMSO-*d*_6_) *δ* = 161, 152.7, 145.4, 144.4, 143.9, 138.6, 133, 132.2, 130.4, 128.6, 127.9, 126.4, 124.2, 123.9, 123.3, 122.3, 121.8, 118.5, 117.2, 115.8, 115.2, 114.2, 99.5, EI-MS (70 eV): *m*/*z* 407 [M]^+^˙, 298 (base peak).

#### 3.9.4. 7-(2-Methoxyphenyl)-7,14-dihydro-6*H*-benzo[*h*]chromeno[4,3-*b*]quinolin-6-one (a4)

Cream solid, M.p. >300 °C, IR (KBr, cm^−1^): 3345, 3068, 1666, 1622, 1519, 1473, 1240. ^1^H NMR (250 MHz, DMSO-*d*_6_) *δ* = 9.35 (s, 1H), 8.56 (t, *J* = 7.6 Hz, 2H), 7.83 (d, *J* = 8.1 Hz, 1H), 7.63 (dd, *J* = 13.8, 7.6 Hz, 2H), 7.50 (dt, *J* = 8.5, 4.2 Hz, 3H), 7.39 (d, *J* = 8.4 Hz, 2H), 7.10 (t, *J* = 8.6 Hz, 2H), 6.95 (d, *J* = 8.1 Hz, 1H), 6.76 (t, *J* = 7.3 Hz, 1H), 5.68 (d, *J* = 2.6 Hz, 1H), 3.83 (s, 3H). ^13^C NMR (62.5 MHz, DMSO-*d*_6_) *δ* = 160.7, 156.1, 152.8, 145.2, 136, 132.9, 132.2, 130.3, 129, 128.5, 128.2, 127.3, 126.3, 124.1, 123.2, 122.2, 121.4, 121.2, 117.2, 114.1, 112.1, 98.5, 56.2, 35.6, EI-MS (70 eV): *m*/*z* 405 [M]^+^˙, 298 (base peak).

#### 3.9.5. 7-(2-Chlorophenyl)-7,14-dihydro-6*H*-benzo[*h*]chromeno[4,3-*b*]quinolin-6-one (a5)

Cream solid, M.p. >300 °C, IR (KBr, cm^−1^): 3322, 3061, 1664, 1620, 1519, 1469, 1273. ^1^H NMR (250 MHz, DMSO-*d*_6_) *δ* = 9.36 (s, 1H), 8.59 (dd, *J* = 13.1, 8.3 Hz, 2H), 7.84 (d, *J* = 8.1 Hz, 1H), 7.65 (d, *J* = 8.4 Hz, 2H), 7.53 (dd, *J* = 11.6, 7.8 Hz, 4H), 7.37 (d, *J* = 8.1 Hz, 2H), 7.25 (d, *J* = 8.6 Hz, 1H), 7.16–7.10 (m, 2H), 5.90 (s, 1H). ^13^C NMR (62.5 MHz, DMSO-*d*_6_) *δ* = 152.8, 145.3, 133.1, 133, 131.4, 131.1, 130.2, 129.8, 128.6, 128.2, 126.8, 126.6, 124.5, 124.2, 123.2, 122.4, 120.2, 117.2, 113.8, 39, EI-MS (70 eV): *m*/*z* 409 [M]^+^˙, 298 (base peak).

#### 3.9.6. 7-(2,3-Dihydroxyphenyl)-7,14-dihydro-6*H*-benzo[*h*]chromeno[4,3-*b*]quinolin-6-one (a6)

Yellow solid, M.p. 285–289 °C, IR (KBr, cm^−1^): 3376, 3045, 1648, 1522, 1474, 1266. ^1^H NMR (250 MHz, DMSO-*d*_6_) *δ* = 9.30 (d, *J* = 13.7 Hz, 2H), 8.66–8.47 (m, 3H), 7.83 (d, *J* = 7.8 Hz, 1H), 7.65–7.32 (m, 7H), 6.56–6.30 (m, 3H), 5.80 (s, 1H). ^13^C NMR (62.5 MHz, DMSO-*d*_6_) *δ* = 161, 152.7, 145.6, 145.1, 142, 135.1, 133, 132.2, 130, 128.5, 127.5, 126.3, 124.2, 123.9, 123.1, 122.2, 119.5, 119.2, 117.2, 114.1, 113.7, 99, 34.9, EI-MS (70 eV): *m*/*z* 407 [M]^+^˙, 242 (base peak).

#### 3.9.7. 7-(4-Methoxyphenyl)-7,14-dihydro-6*H*-benzo[*h*]chromeno[4,3-*b*]quinolin-6-one (a7)

Yellow solid, M.p. >300 °C, IR (KBr, cm^−1^): 3296, 1664, 1621, 1520, 1402, 1245. ^1^H NMR (250 MHz, DMSO-*d*_6_) *δ* = 9.43 (s, 1H), 8.60 (dd, *J* = 18.4, 8.2 Hz, 2H), 7.88 (d, *J* = 7.8 Hz, 1H), 7.65–7.30 (m, 7H), 7.15 (d, *J* = 8.2 Hz, 2H), 6.78 (d, *J* = 8.2 Hz, 2H), 5.31 (s, 1H), 3.61 (s, 3H). ^13^C NMR (62.5 MHz, DMSO-*d*_6_) *δ* = 160.9, 158.4, 152.7, 144.1, 139.7, 133.1, 132.2, 130.4, 128.8, 128.6, 127.9, 126.5, 126.4, 124.4, 124.1, 124, 123.2, 122.3, 121.2, 117.2, 114.3, 99.4, 55.4, 41.1, EI-MS (70 eV): *m*/*z* 405 [M]^+^˙, 298 (base peak).

#### 3.9.8. 7-(4-Isopropylphenyl)-7,14-dihydro-6*H*-benzo[*h*]chromeno[4,3-*b*]quinolin-6-one (a8)

Yellow solid, M.p. >300 °C, IR (KBr, cm^−1^): 3354, 2958, 1666, 1619, 1525, 1480, 1270. ^1^H NMR (250 MHz, DMSO-*d*_6_) *δ* = 9.50 (s, 1H), 8.62 (dd, *J* = 16.6, 8.1 Hz, 2H), 7.88 (d, *J* = 8.1 Hz, 2H), 7.56 (ddt, *J* = 25.4, 18.8, 8.3 Hz, 6H), 7.36 (t, *J* = 7.8 Hz, 2H), 7.20 (d, *J* = 7.8 Hz, 2H), 7.03 (d, *J* = 7.6 Hz, 2H), 5.33 (s, 1H), 2.71 (d, *J* = 6.9 Hz, 1H), 1.06 (d, *J* = 6.9 Hz, 6H). ^13^C NMR (62.5 MHz, DMSO-*d*_6_) *δ* = 161, 156.8, 154.1, 152.7, 147.6, 147, 144.9, 139.4, 135, 132.3, 130.5, 128.6, 127.6, 126.8, 126.6, 124.5, 123.9, 121.7, 117.2, 38.1, 33.4, 24.2, EI-MS (70 eV): *m*/*z* 417 [M]^+^˙, 242 (base peak).

#### 3.9.9. 7-(Pyridin-2-yl)-7,14-dihydro-6*H*-benzo[*h*]chromeno[4,3-*b*]quinolin-6-one (a9)

Brown solid, M.p. >300 °C, IR (KBr, cm^−1^): 3419, 3045, 1681, 1636, 1589, 1526, 1480, 1266. ^1^H NMR (250 MHz, DMSO-*d*_6_) *δ* = 9.47 (s, 1H), 8.61–8.49 (m, 2H), 8.32 (s, 1H), 7.85 (d, *J* = 8.1 Hz, 1H), 7.65 (d, *J* = 7.6 Hz, 2H), 7.58–7.35 (m, 7H), 7.13–7.04 (m, 1H), 5.50 (s, 1H). ^13^C NMR (62.5 MHz, DMSO-*d*_6_) *δ* = 164.5, 152.8, 149.8, 145.1, 137.1, 133.1, 132.3, 130.7, 128.6, 127.7, 126.6, 126.3, 124.1, 123.2, 122.3, 122.1, 120, 117.2, 114.2, 97.9, 44.5, EI-MS (70 eV): *m*/*z* 376 [M]^+^˙, 298 (base peak).

#### 3.9.10. 7-(Thiophen-2-yl)-7,14-dihydro-6*H*-benzo[*h*]chromeno[4,3-*b*]quinolin-6-one (a10)

Yellow solid, M.p. 210 °C, IR (KBr, cm^−1^): 3296, 3059, 2926, 1677, 1620, 1523, 1395, 1269. ^1^H NMR (250 MHz, DMSO-*d*_6_) *δ* = 9.70 (s, 1H), 8.61 (d, *J* = 8.2 Hz, 1H), 8.57 (d, *J* = 7.8 Hz, 1H), 7.88 (d, *J* = 12.2 Hz, 2H), 7.65 (d, *J* = 7.5 Hz, 3H), 7.50–7.39 (m, 3H), 7.20 (d, *J* = 4.8 Hz, 1H), 6.80 (dt, *J* = 6.8, 3.5 Hz, 2H), 5.69 (s, 1H). ^13^C NMR (62.5 MHz, DMSO-*d*_6_) *δ* = 161, 152.8, 151.2, 144.7, 136.4, 134.9, 133.3, 132.3, 131, 128.6, 127.8, 127.2, 126.7, 126.4, 124.9, 124.4, 124.1, 124, 123.5, 122.5, 120.4, 117.2, 49.1, 37.1, EI-MS (70 eV): *m*/*z* 381 [M]^+^˙, 298 (base peak).

### 3.10. Proposed mechanism for the synthesis of a(1–10) by ETPPBr/2,3-DHBA-DES

**Fig. 10:**
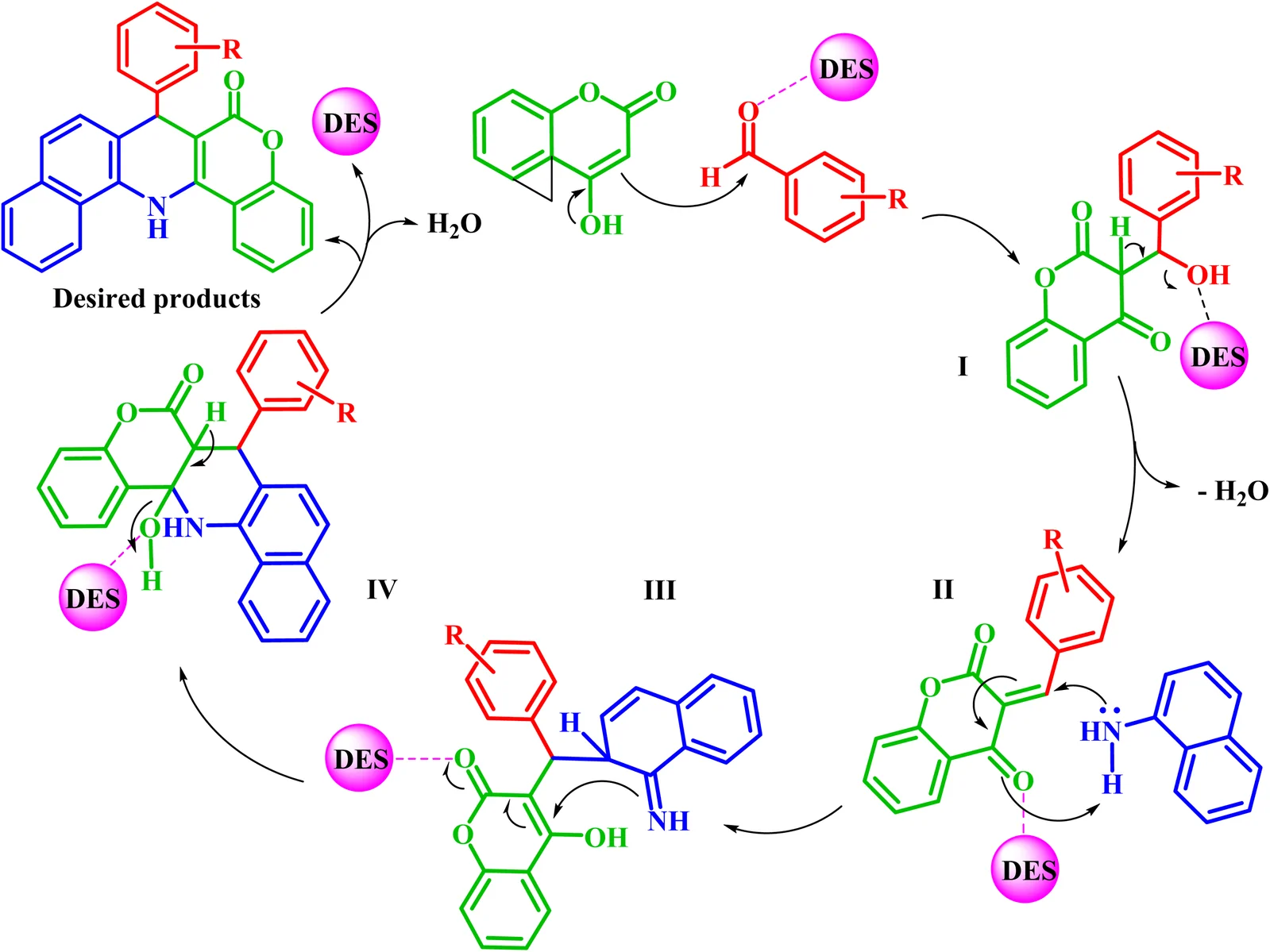
Proposed mechanism for the synthesis of a(1–10) catalyzed by ETPPBr/2,3-DHBA-DES.

A plausible mechanism for the synthesis of a(1–10) catalyzed by ETPPBr/2,3-DHBA-DES is illustrated in Fig. 10. Initially, the aldehyde carbonyl group is likely activated—either through hydrogen-bonding interactions with the acidic protons of the carboxylic acid or *via* proton transfer from the DES—thereby increasing the polarity of the carbonyl bond and facilitating the Knoevenagel reaction with 4-hydroxycoumarin to yield intermediate I. Subsequent dehydration from intermediate I produces intermediate II, and the nucleophilic attack of 1-naphthylamine on intermediate II affords intermediate III. Intramolecular cyclization of intermediate III leads to the formation of intermediate IV, and its dehydration yields the desired product a(1–10).

### 3.11. Reusability of ETPPBr/2,3-DHBA-DES

**Fig. 11:**
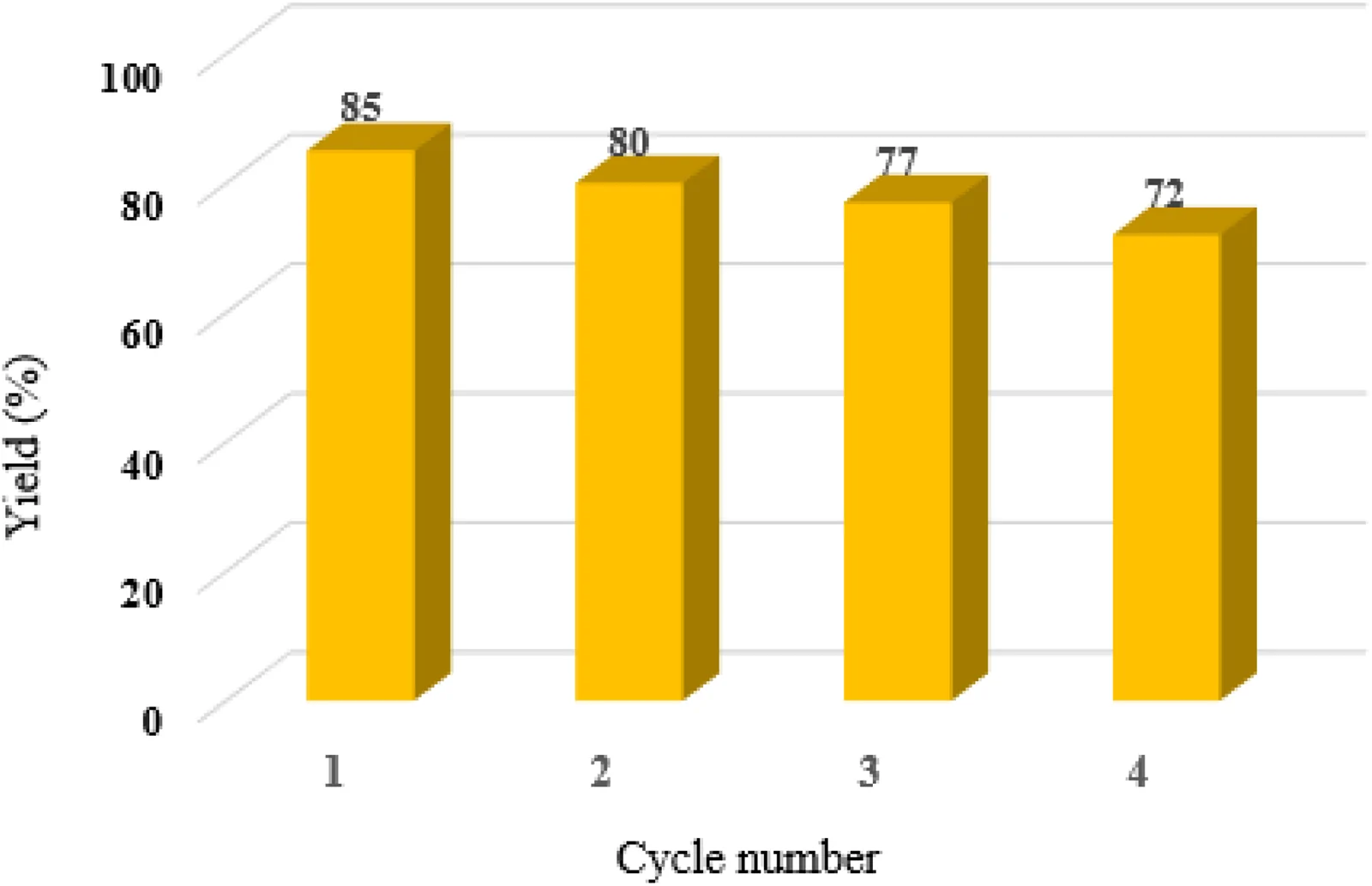
Reusability of ETPPBr/2,3-DHBA-DES in the synthesis of compound a1.

The recyclability of ETPPBr/2,3-DHBA-DES was evaluated using the model reaction for the synthesis of compound a1. After completion of the reaction, the product was separated by filtration. The remaining reaction mixture containing the DES catalyst was extracted with equal volumes of water and ethyl acetate (or chloroform). The aqueous phase containing the DES was collected, dried, and directly reused in subsequent reaction cycles without further purification. As shown in Fig. 11, the catalyst retained acceptable catalytic activity after four consecutive cycles, although a gradual decrease in yield was observed, which may be attributed to minor catalyst loss during the recovery process.

The recovered DES was characterized by FT-IR after the fourth catalytic cycle, and no significant changes in the characteristic absorption bands were observed, indicating that the hydrogen-bond network of the DES was largely preserved during the recycling process (Fig. 5).

### 3.12. Comparison of the catalytic performance with previously reported systems

The catalytic performance of ETPPBr/2,3-DHBA-DES was compared with previously reported catalytic systems based on average reaction times and isolated yields of the synthesized derivatives (Table 5). Although the reported MOF-based catalysts generally provided higher yields and shorter reaction times, their preparation involves multistep procedures using metal precursors. In contrast, the present DES was prepared within 30 min by simple mixing of its components, without the need for organic solvents, post-synthetic purification, or activation. These features, together with its recyclability, make ETPPBr/2,3-DHBA-DES a simple and practical catalytic system for this transformation.

**Table 5:** Comparison of catalytic systems for the synthesis of a1

Entry	Catalyst	Preparation time	Metal-free	Conditions	Time (min)	Yield%	Ref.
1	Basu-DPU	2 days	No	Solvent-free, 110 °C	10	95	34
	Zr-MOF						
2	Ce-MOF-EGTA	4 days	No	Solvent-free, 80 °C	10	95	35
3	ETPPBr/2,3-DHBA-DES	30 min	Yes	Solvent-free, 80 °C	45	85	This work

## 4. Conclusion

In this study, ETPPBr/2,3-DHBA-DES was successfully prepared, characterized, and applied as a catalyst for the solvent-free synthesis of a(1–10). The prepared DES exhibited good catalytic activity, affording the desired products in good to excellent yields under mild reaction conditions. Furthermore, it showed good thermal stability and could be recovered and reused for four consecutive reaction cycles with only a gradual decrease in catalytic performance. These findings demonstrate that phosphonium-based deep eutectic solvents can serve as reusable and environmentally benign catalytic systems for multicomponent reactions and provide a useful platform for the development of sustainable synthetic methodologies.

## Conflicts of interest

There are no conflicts to declare.

## Supplementary Material

RA-OLF-D6RA07325E-s001

## Data Availability

All data generated or analyzed during this study are included in the main body of the text as well as the supplementary information (SI) file. Supplementary information is available. See DOI: https://doi.org/10.1039/d6ra07325e.
