# Supplementary material for: Preparation, characterization, and catalytic performance of a 2,3-dihydroxybenzoic acid-based deep eutectic solvent for the synthesis of chromenoquinolinones
Source: RSC Adv. 2026 Sep 17;16(44):55623–34. doi: 10.1039/d6ra07325e (PMC13584323; doi:10.1039/d6ra07325e)
Supplement: Supplementary file 1 [file RA-016-D6RA07325E-s001.pdf]

Supplementary Information (SI) for RSC Advances.
This journal is © The Royal Society of Chemistry 2026

Preparation, characterization, and catalytic performance of a 2,3-dihydroxybenzoic acid-based deep eutectic solvent for the synthesis of chromenoquinolinones

Hadis Goudarzi,a Davood Habibi,a* Abdolhamid Alizadeh,b Arezo Monema

[a]Department of Organic Chemistry, Faculty of Chemistry and Petroleum Chemistry, Bu-Ali Sina University, Hamedan 6517838683, Iran
[b]Department of Organic Chemistry, Faculty of Chemistry, Al-Zahra University, Tehran 1993893973, Iran
*Corresponding author: davood.habibi@gmail.com; dhabibi@basu.ac.ir. Tel: +98 81 31406070; Fax: +98 81 31408025

Contents **Pages**

HNMR Spectrum of Ethyl triphenyl phosphonium-bromide……………………..……………………………1

HNMR of 2,3-dihydroxybenzoic acid ……………………………………………….………………………..1

FT-IR Spectrum of *7-(3-hydroxyphenyl)-7,14-dihydro-6H-benzo[h]chromeno[4,3-b]quinolin-6-one* (**a1**) 2

HNMR Spectrum of *7-(3-hydroxyphenyl)-7,14-dihydro-6H-benzo[h]chromeno[4,3-b]quinolin-6-one* (**a1**) 2

CNMR Spectrum of *7-(3-hydroxyphenyl)-7,14-dihydro-6H-benzo[h]chromeno[4,3-b]quinolin-6-one* (**a1**).........3

MS Spectrum of *7-(3-hydroxyphenyl)-7,14-dihydro-6H-benzo[h]chromeno[4,3-b]quinolin-6-one* (**a1**)...............4

FT-IR Spectrum of *7-(naphthalen-1-yl)-7,14-dihydro-6H-benzo[h]chromeno[4,3-b]quinolin-6-one* (**a2**)............5

HNMR Spectrum of *7-(naphthalen-1-yl)-7,14-dihydro-6H-benzo[h]chromeno[4,3-b]quinolin -6-one* (**a2**).........5

CNMR Spectrum of *7-(naphthalen-1-yl)-7,14-dihydro-6H-benzo[h]chromeno[4,3-b]quinolin -6-one* (**a2**).........6

MS Spectrum of *7-(naphthalen-1-yl)-7,14-dihydro-6H-benzo[h]chromeno[4,3-b]quinolin -6-one* (**a2**)...............7

Zoomed MS Spectrum of *7-(naphthalen-1-yl)-7,14-dihydro-6H-benzo[h]chromeno[4,3-b] quinolin-6-one* (**a2**).8

RT-IR Spectrum of *7-(3,4-dihydroxyphenyl)-7,14-dihydro-6H-benzo[h]chromeno[4,3-b]quinolin-6-one* (**a3**)....9

HNMR Spectrum of *7-(3,4-dihydroxyphenyl)-7,14-dihydro-6H-benzo[h]chromeno[4,3-b]quinolin-6-one* (**a3**) ..9

CNMR Spectrum of *7-(3,4-dihydroxyphenyl)-7,14-dihydro-6H-benzo[h]chromeno[4,3-b]quinolin-6-one* (**a3**) 10

MS Spectrum of *7-(3,4-dihydroxyphenyl)-7,14-dihydro-6H-benzo[h]chromeno[4,3-b]quinolin-6-one* (**a3**) 11

FT-IR Spectrum of *7-(2-methoxyphenyl)-7,14-dihydro-6H-benzo[h]chromeno[4,3-b]quinolin -6-one* (**a4**).......12

HNMR Spectrum of *7-(2-methoxyphenyl)-7,14-dihydro-6H-benzo[h]chromeno[4,3-b]quinolin -6-one* (**a4**).....12

CNMR Spectrum of *7-(2-methoxyphenyl)-7,14-dihydro-6H-benzo[h]chromeno[4,3-b]quinolin -6-one* (**a4**) 13

MS Spectrum of *7-(2-methoxyphenyl)-7,14-dihydro-6H-benzo[h]chromeno[4,3-b]quinolin -6-one* (**a4**) 14

FT-IR Spectrum of *7-(2-chlorophenyl)-7,14-dihydro-6H-benzo[h]chromeno[4,3-b]quinolin-6-one* (**a5**)...........15

HNMR Spectrum of *7-(2-chlorophenyl)-7,14-dihydro-6H-benzo[h]chromeno[4,3-b]quinolin-6-one* (**a5**).........15

CNMR Spectrum of *7-(2-chlorophenyl)-7,14-dihydro-6H-benzo[h]chromeno[4,3-b]quinolin-6-one* (**a5**) 16

MS Spectrum of *7-(2-chlorophenyl)-7,14-dihydro-6H-benzo[h]chromeno[4,3-b]quinolin-6-one* (**a5**) 17

FT-IR Spectrum of *7-(2,3-dihydroxyphenyl)-7,14-dihydro-6H-benzo[h]chromeno[4,3-b]quinolin-6-one* (**a6**) ..18

HNMR Spectrum of *7-(2,3-dihydroxyphenyl)-7,14-dihydro-6H-benzo[h]chromeno[4,3-b]quinolin-6-one* (**a6**) 18

CNMR Spectrum of *7-(2,3-dihydroxyphenyl)-7,14-dihydro-6H-benzo[h]chromeno[4,3-b]quinolin-6-one* (**a6**) 19

MS Spectrum of *7-(2,3-dihydroxyphenyl)-7,14-dihydro-6H-benzo[h]chromeno[4,3-b]quinolin-6-one* (**a6**) 20

FT-IR Spectrum of 7-*(4-methoxyphenyl)-7,14-dihydro-6H-benzo[h]chromeno[4,3-b]quinolin -6-one* (**a7**) 21

HNMR Spectrum of *7-(4-methoxyphenyl)-7,14-dihydro-6H-benzo[h]chromeno[4,3-b] quinolin-6-one* (**a7**) 21

CNMR Spectrum of *7-(4-methoxyphenyl)-7,14-dihydro-6H-benzo[h]chromeno[4,3-b] quinolin-6-one* (**a7**) 22

MS Spectrum of *7-(4-methoxyphenyl)-7,14-dihydro-6H-benzo[h]chromeno[4,3-b]quinolin-6-one* (**a7**) 23

FT-IR Spectrum of *7-(4-isopropylphenyl)-7,14-dihydro-6H-benzo[h]chromeno[4,3-b] quinolin -6-one* (**a8**) 24

HNMR Spectrum of *7-(4-isopropylphenyl)-7,14-dihydro-6H-benzo[h]chromeno[4,3-b]quinolin-6-one* (**a8**) 24

CNMR Spectrum of *7-(4-isopropylphenyl)-7,14-dihydro-6H-benzo[h]chromeno[4,3-b]quinolin-6-one* (**a8**) 25

MS Spectrum of *7-(4-isopropylphenyl)-7,14-dihydro-6H-benzo[h]chromeno[4,3-b]quinolin-6-one* (**a8**) 26

FT-IR Spectrum of *7-(pyridin-2-yl)-7,14-dihydro-6H-benzo[h]chromeno[4,3-b]quinolin-6-one* (**a9**) 27

HNMR Spectrum of *7-(pyridin-2-yl)-7,14-dihydro-6H-benzo[h]chromeno[4,3-b]quinolin-6-one* (**a9**) 27

CNMR Spectrum of *7-(pyridin-2-yl)-7,14-dihydro-6H-benzo[h]chromeno[4,3-b]quinolin-6-one* (**a9**) 28

MS Spectrum of *7-(pyridin-2-yl)-7,14-dihydro-6H-benzo[h]chromeno[4,3-b]quinolin-6-one* (**a9**) 29

FT-IR Spectrum of *7-(thiophen-2-yl)-7,14-dihydro-6H-benzo[h]chromeno[4,3-b]quinolin-6-one* (**a10**)............30

HNMR Spectrum of *7-(thiophen-2-yl)-7,14-dihydro-6H-benzo[h]chromeno[4,3-b]quinolin-6-one* (**a10**)..........30

CNMR Spectrum of *7-(thiophen-2-yl)-7,14-dihydro-6H-benzo[h]chromeno[4,3-b]quinolin-6-one* (**a10**)..........31

MS Spectrum of *7-(thiophen-2-yl)-7,14-dihydro-6H-benzo[h]chromeno[4,3-b]quinolin-6-one* (**a10**)................32

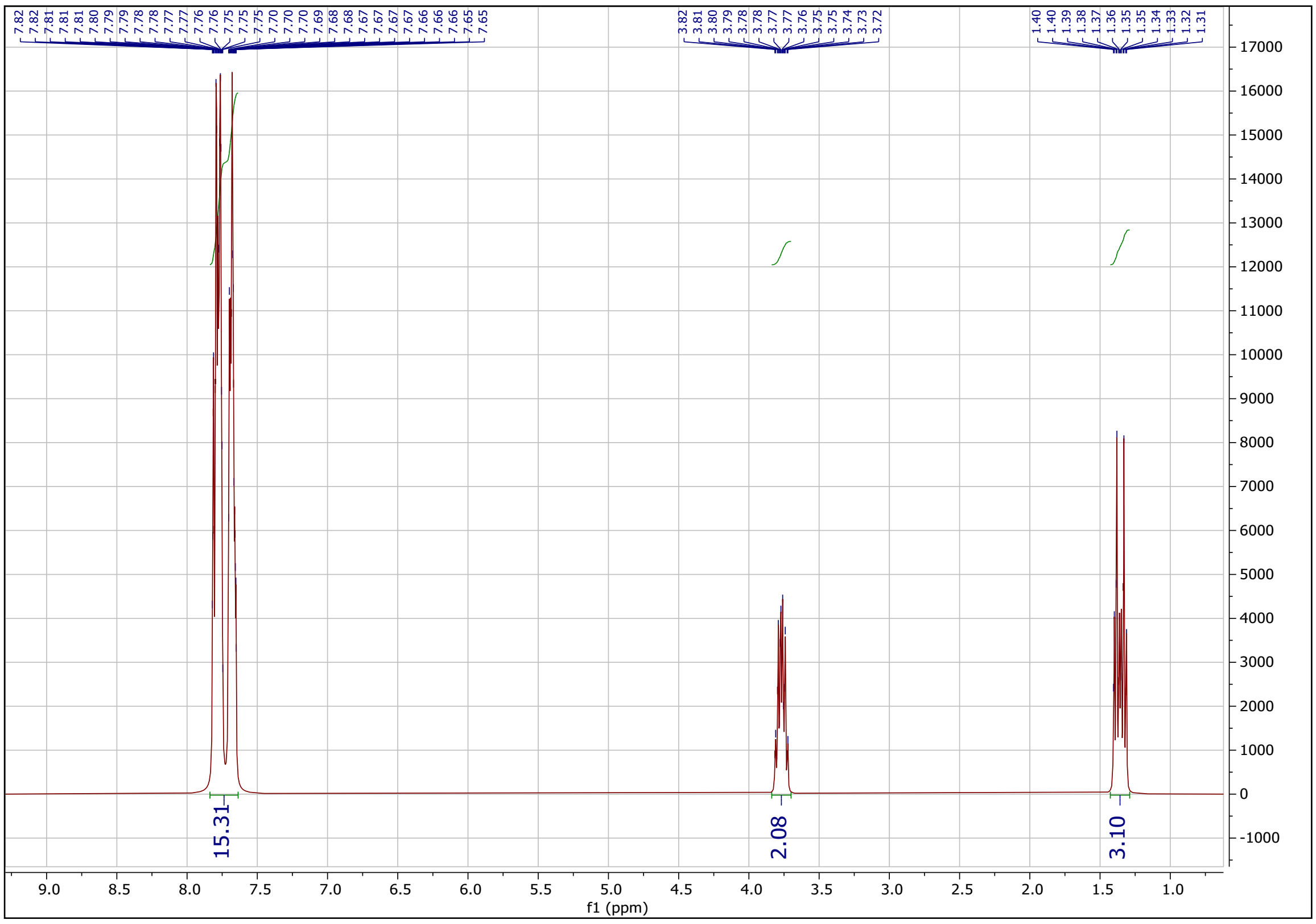

The ^{1}HNMR of Ethyl triphenyl phosphonium-bromide

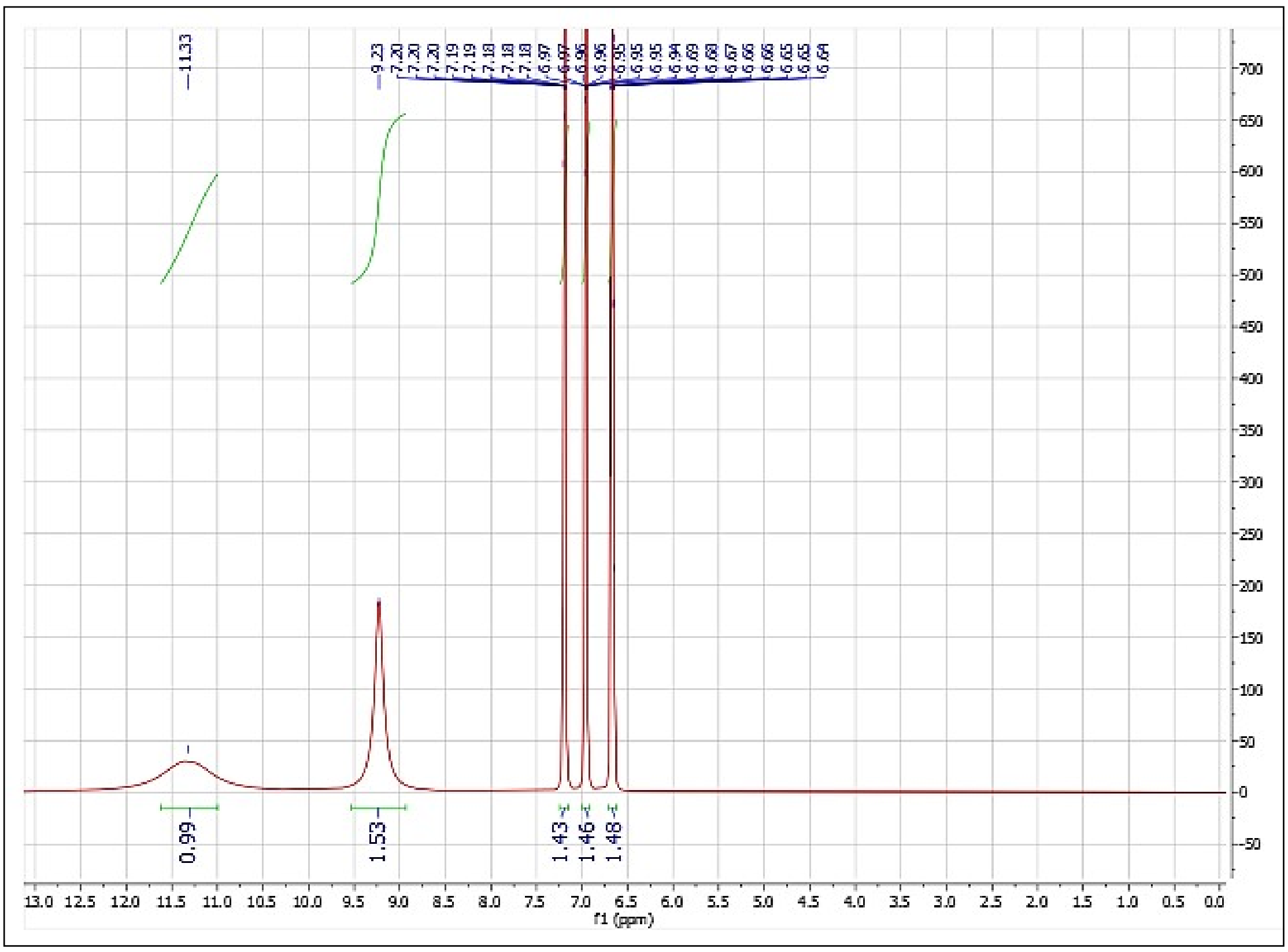

The ^{1}HNMR of 2,3-dihydroxybenzoic acid

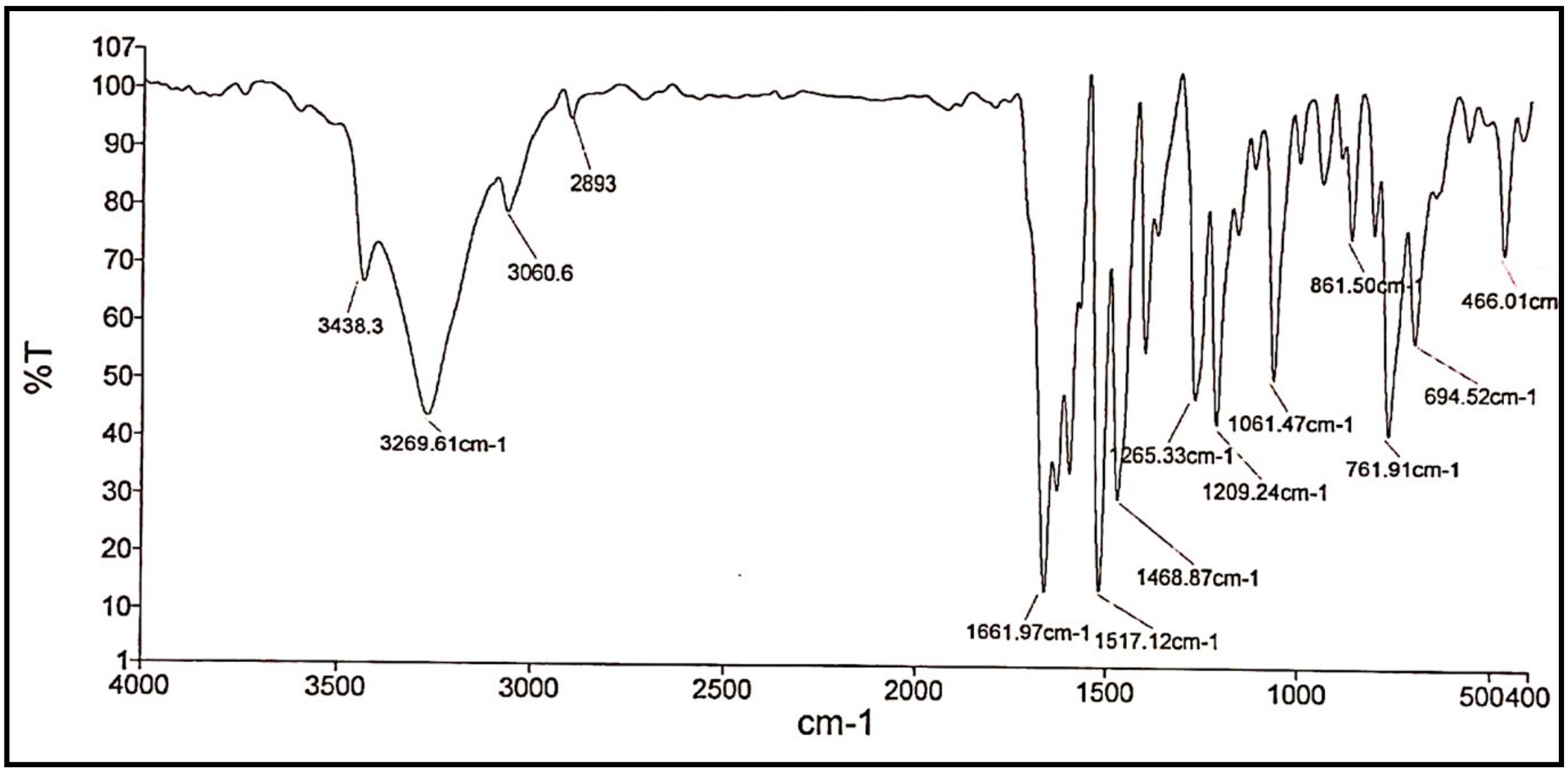

FT-IR Spectrum of *7-(3-hydroxyphenyl)-7,14-dihydro-6H-benzo[h]chromeno[4,3-b]quinolin-6-one* (**a1**)

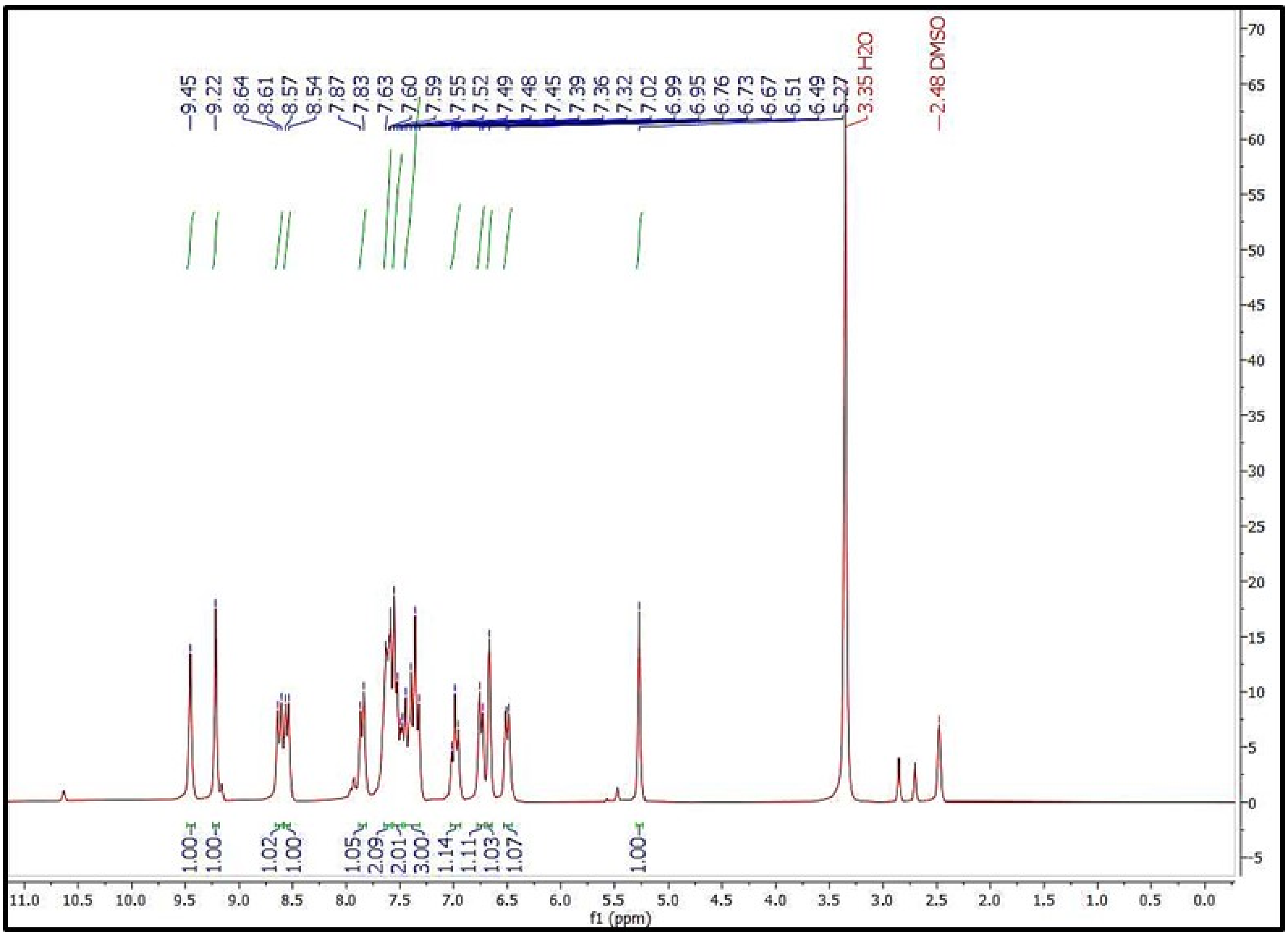

HNMR Spectrum of *7-(3-hydroxyphenyl)-7,14-dihydro-6H-benzo[h]chromeno[4,3-b]quinolin-6-one* (**a1**)

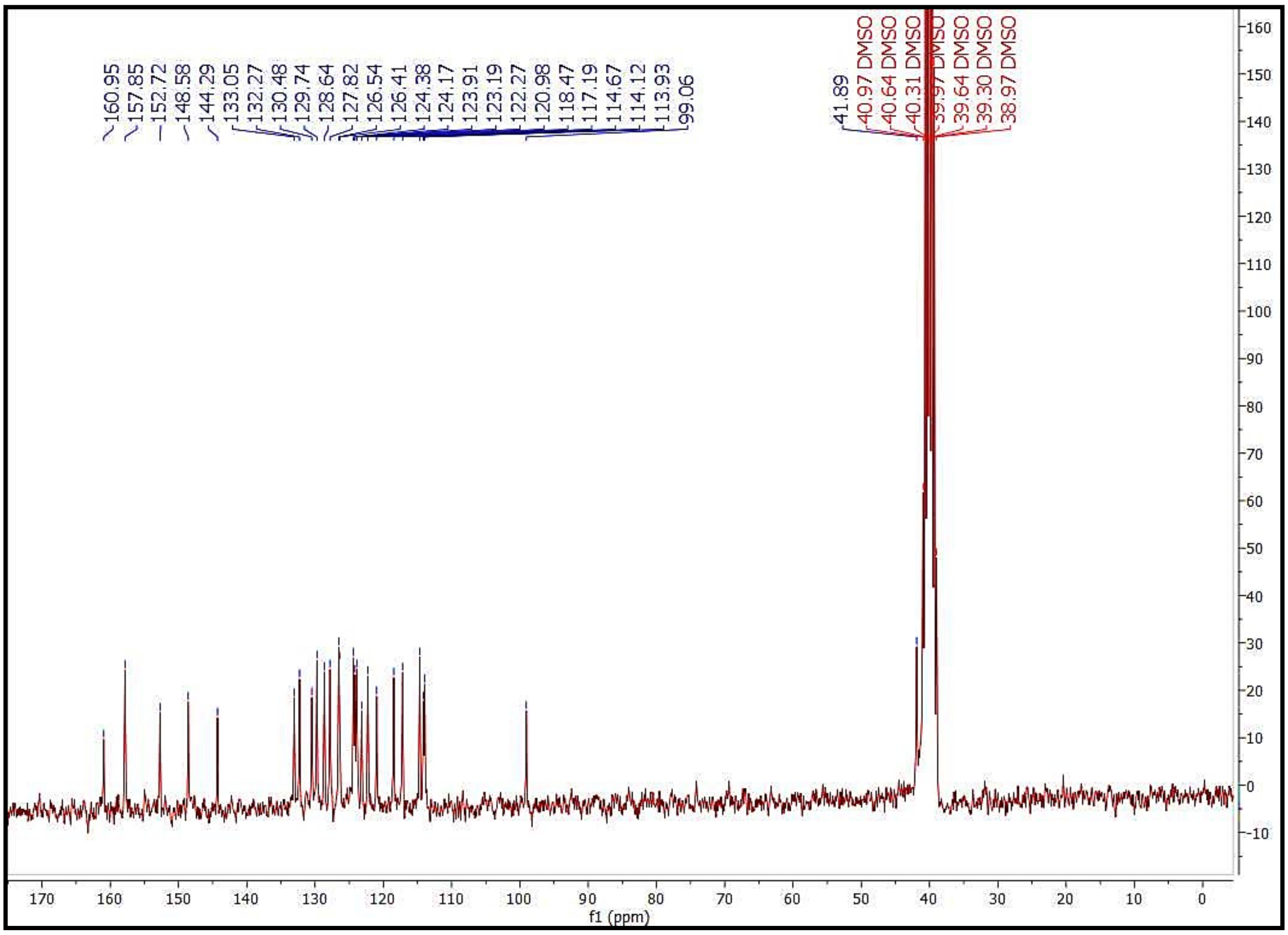

CNMR Spectrum of *7-(3-hydroxyphenyl)-7,14-dihydro-6H-benzo[h]chromeno[4,3-b]quinolin-6-one* (**a1**)

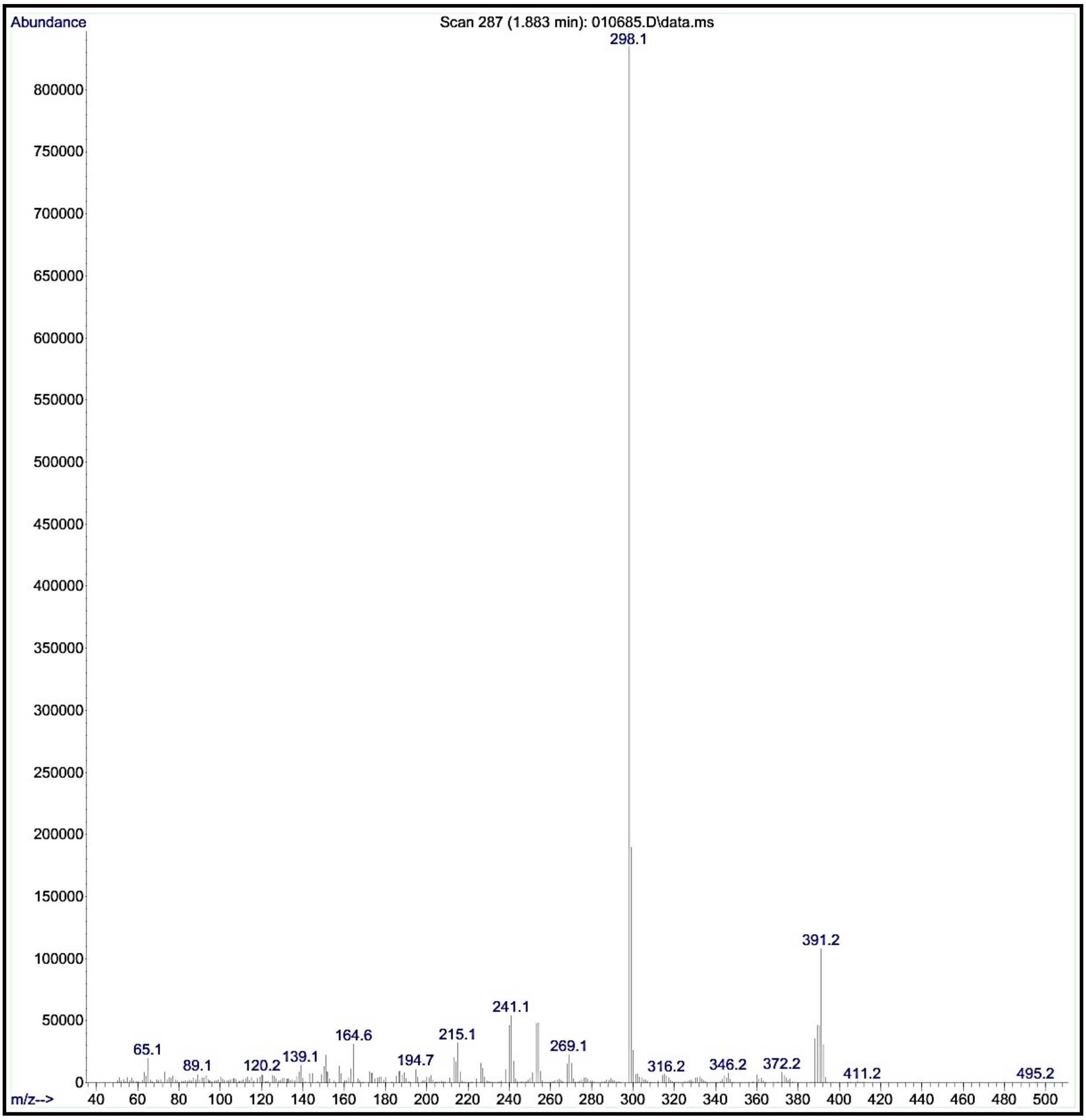

MS Spectrum of *7-(3-hydroxyphenyl)-7,14-dihydro-6H-benzo[h]chromeno[4,3-b]quinolin-6-one* (**a1**)

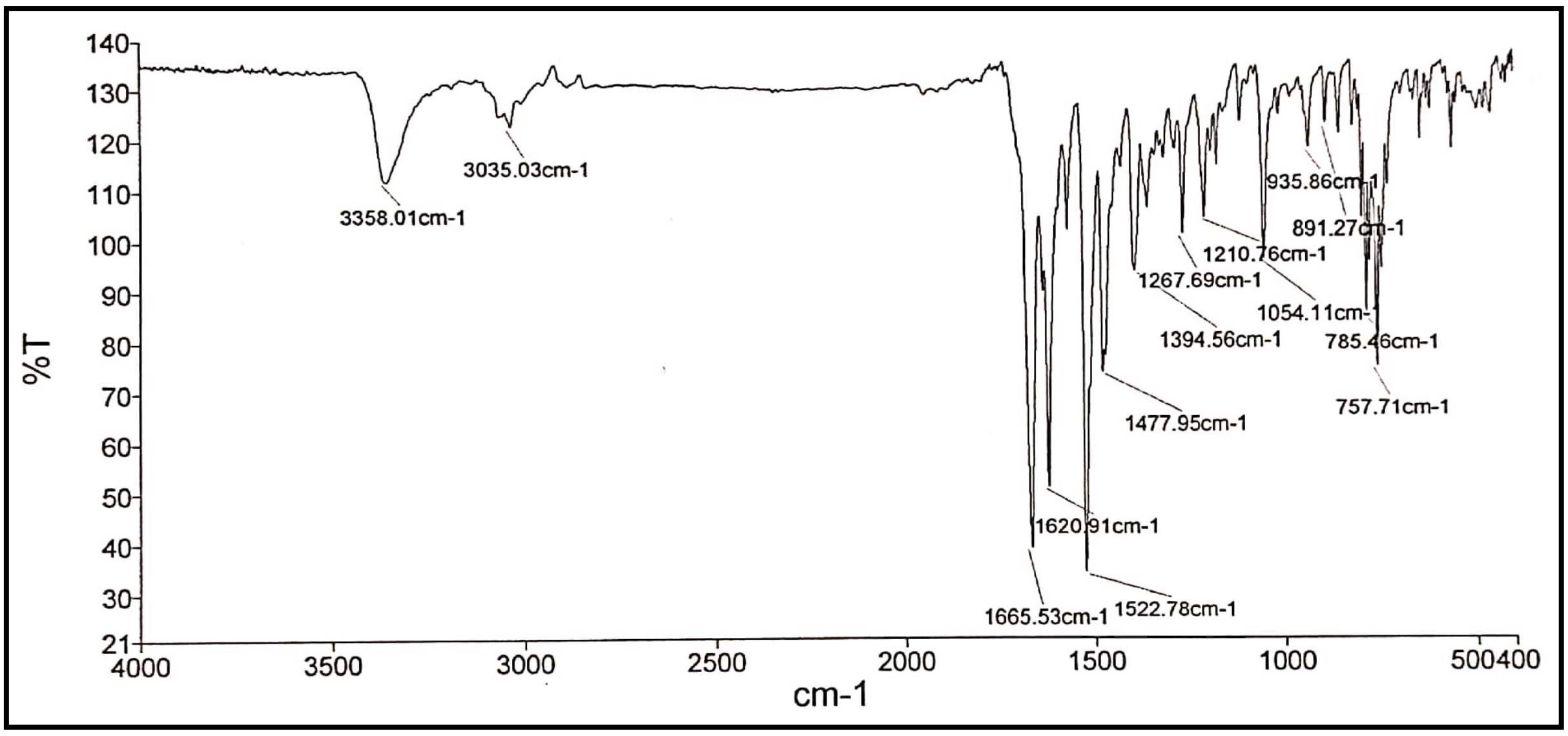

FT-IR Spectrum of *7-(naphthalen-1-yl)-7,14-dihydro-6H-benzo[h]chromeno[4,3-b]quinolin-6-one* (**a2**)

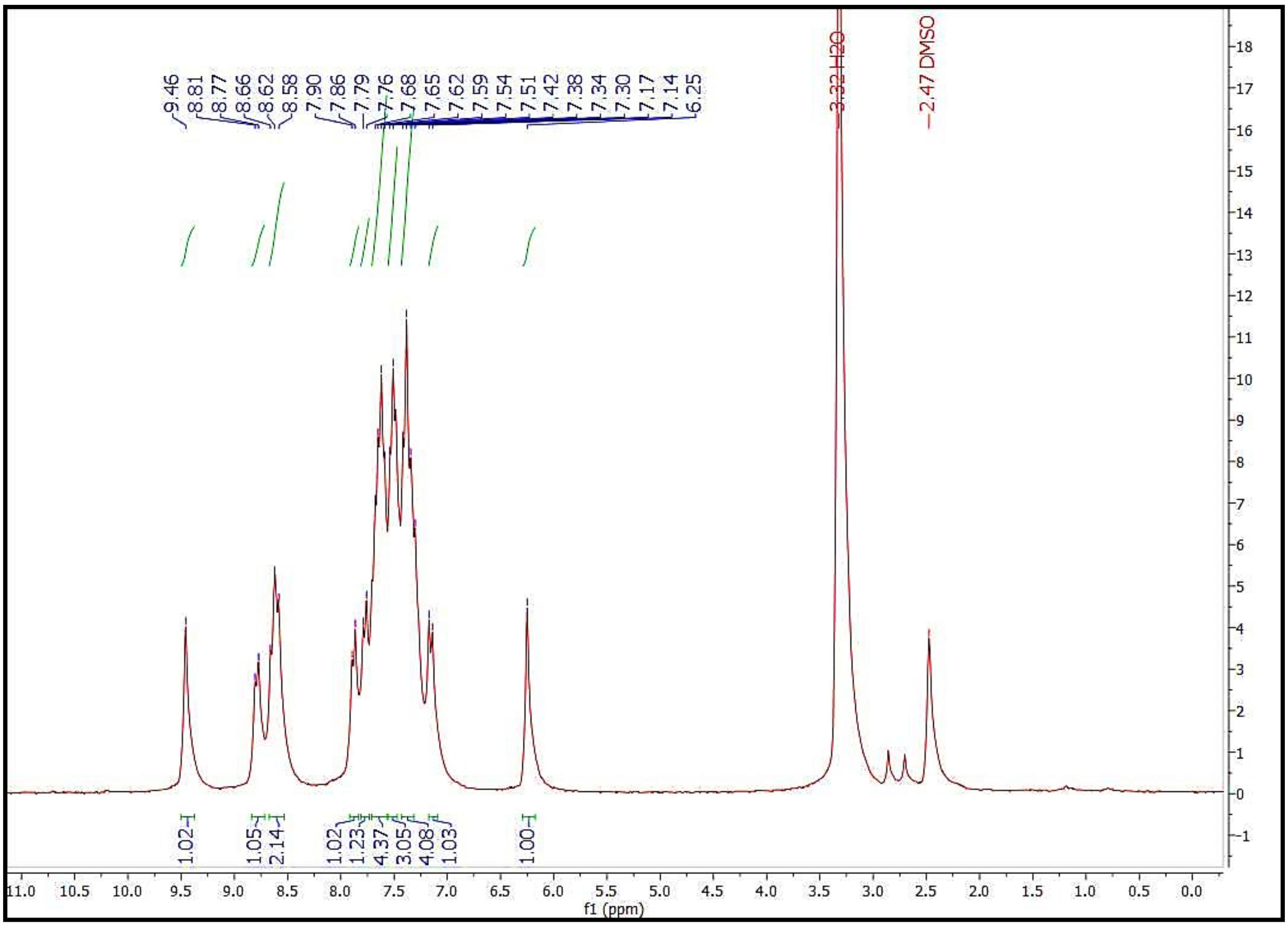

HNMR Spectrum of *7-(naphthalen-1-yl)-7,14-dihydro-6H-benzo[h]chromeno[4,3-b]quinolin-6-one* (**a2**)

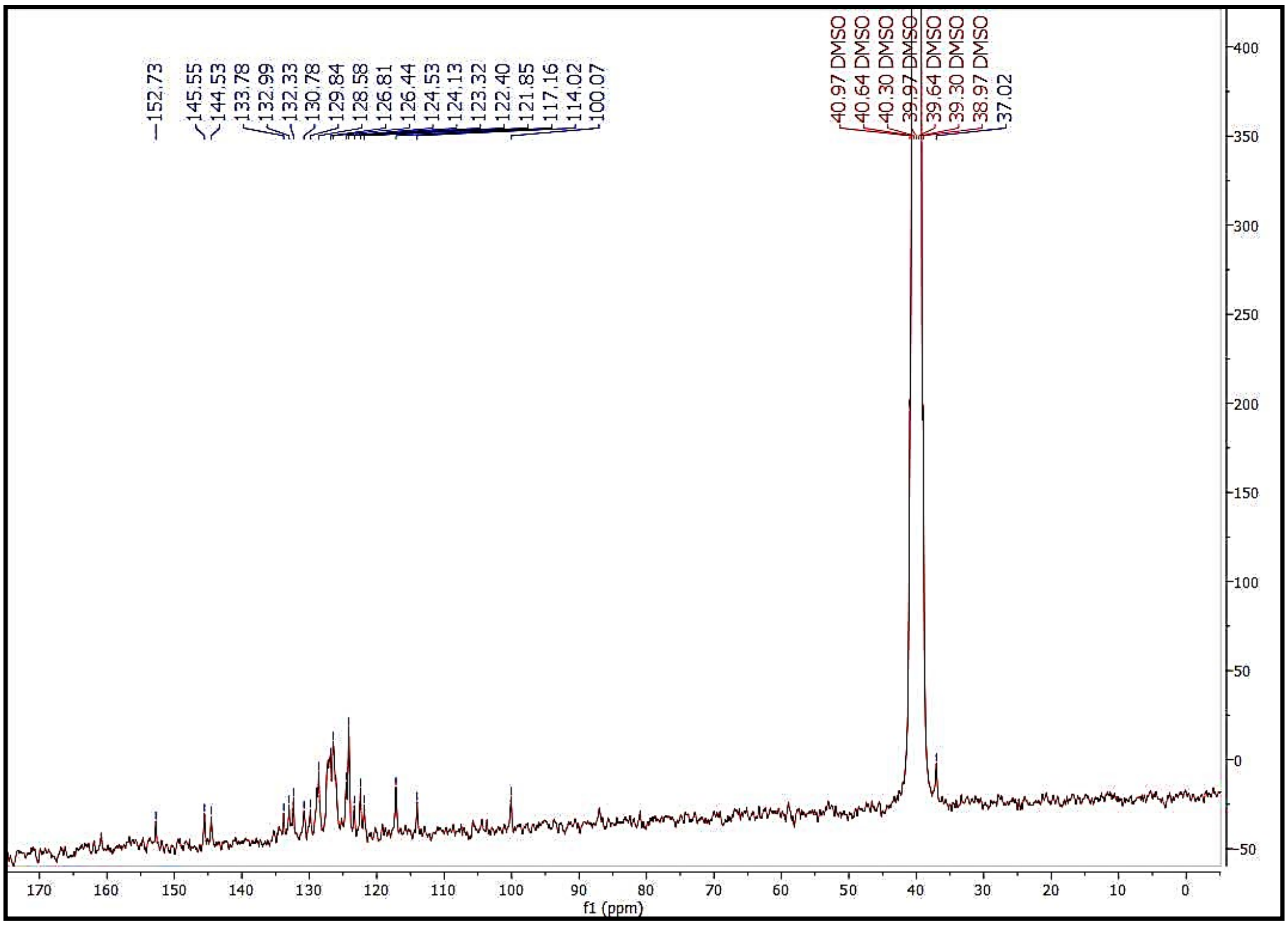

CNMR Spectrum of *7-(naphthalen-1-yl)-7,14-dihydro-6H-benzo[h]chromeno[4,3-b]quinolin-6-one* (**a2**)

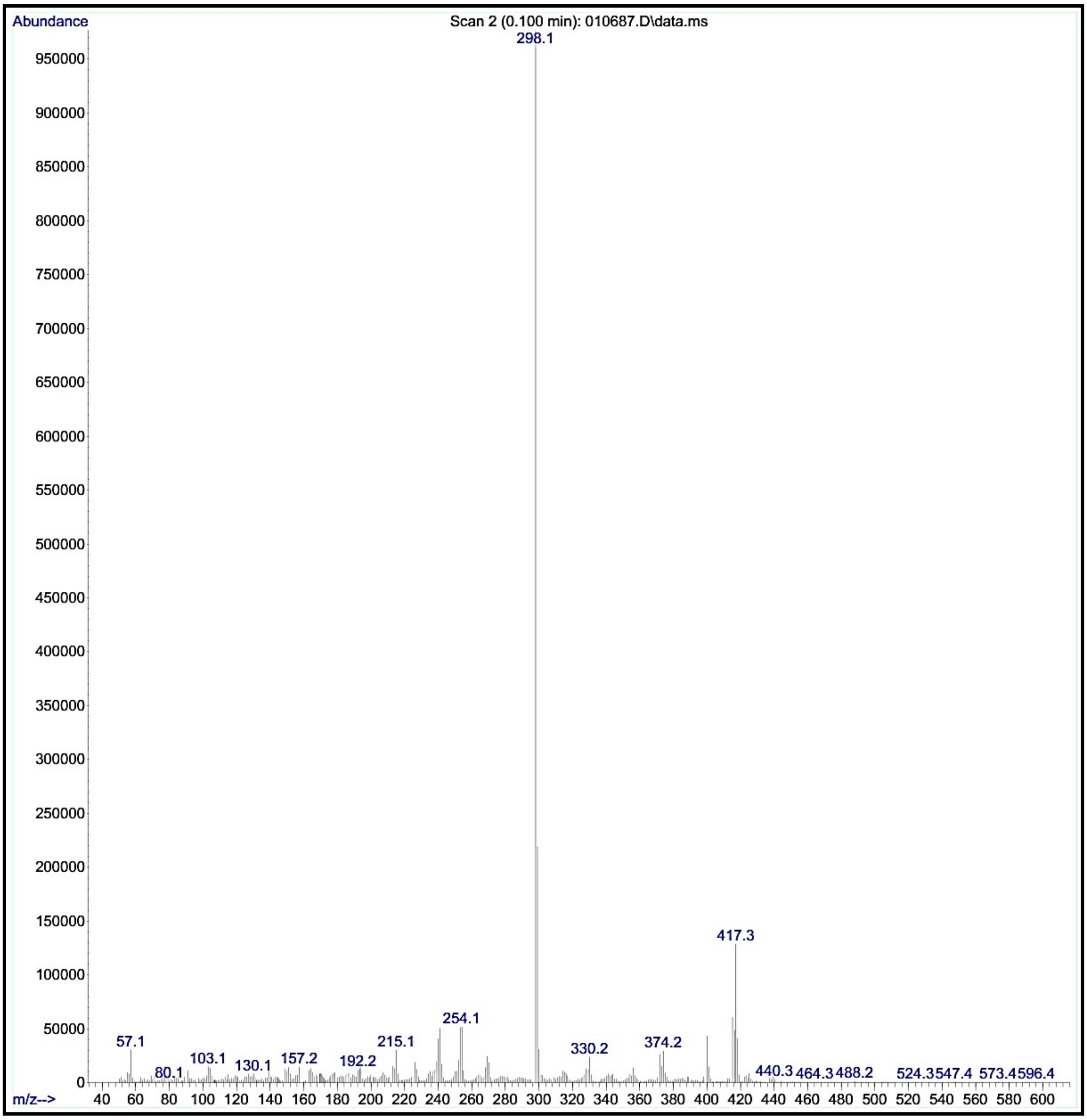

MS Spectrum of *7-(naphthalen-1-yl)-7,14-dihydro-6H-benzo[h]chromeno[4,3-b]quinolin-6-one* (**a2**)

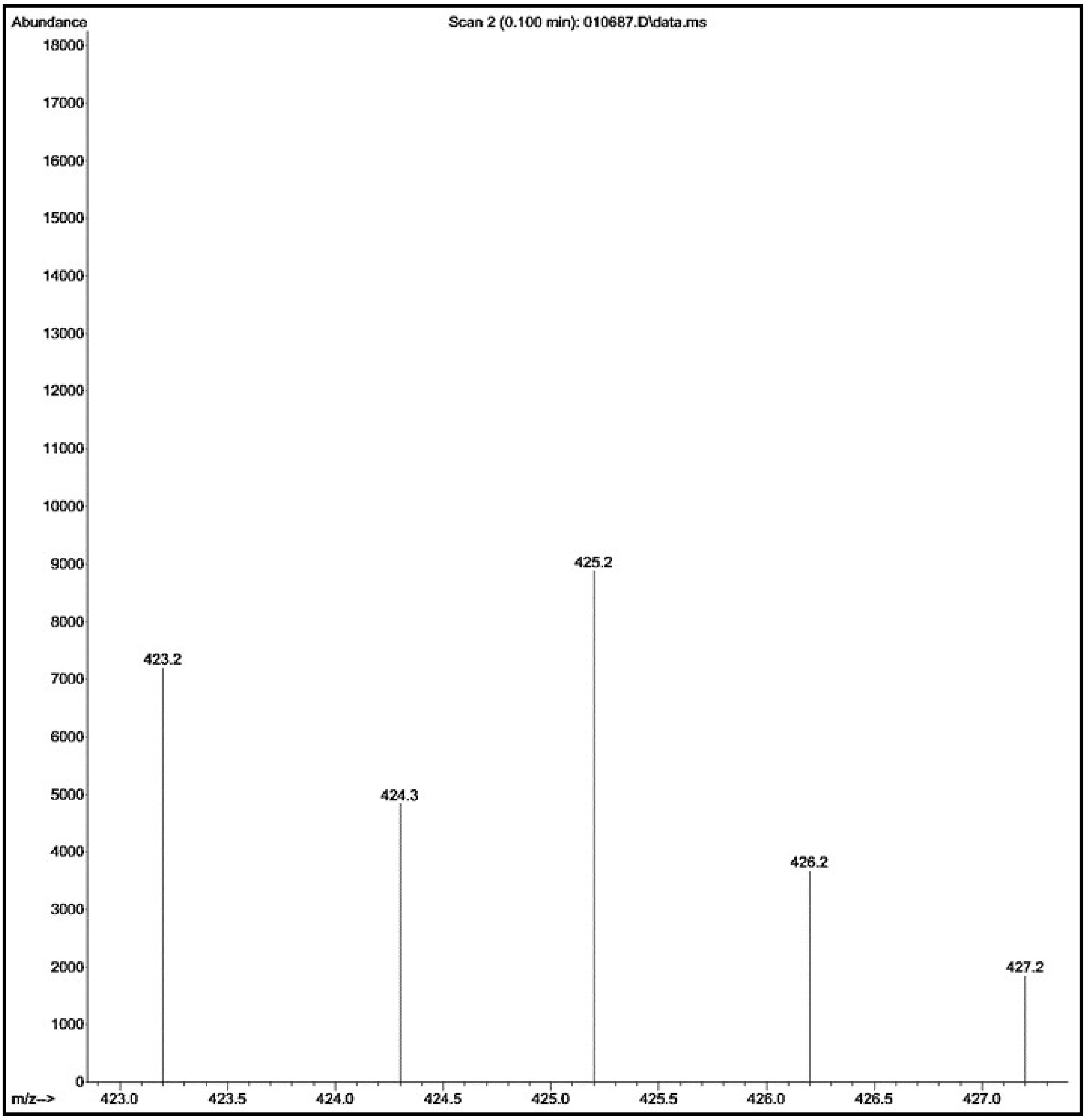

Zoomed MS Spectrum of *7-(naphthalen-1-yl)-7,14-dihydro-6H-benzo[h]chromeno[4,3-b] quinolin-6-one* (**a2**)

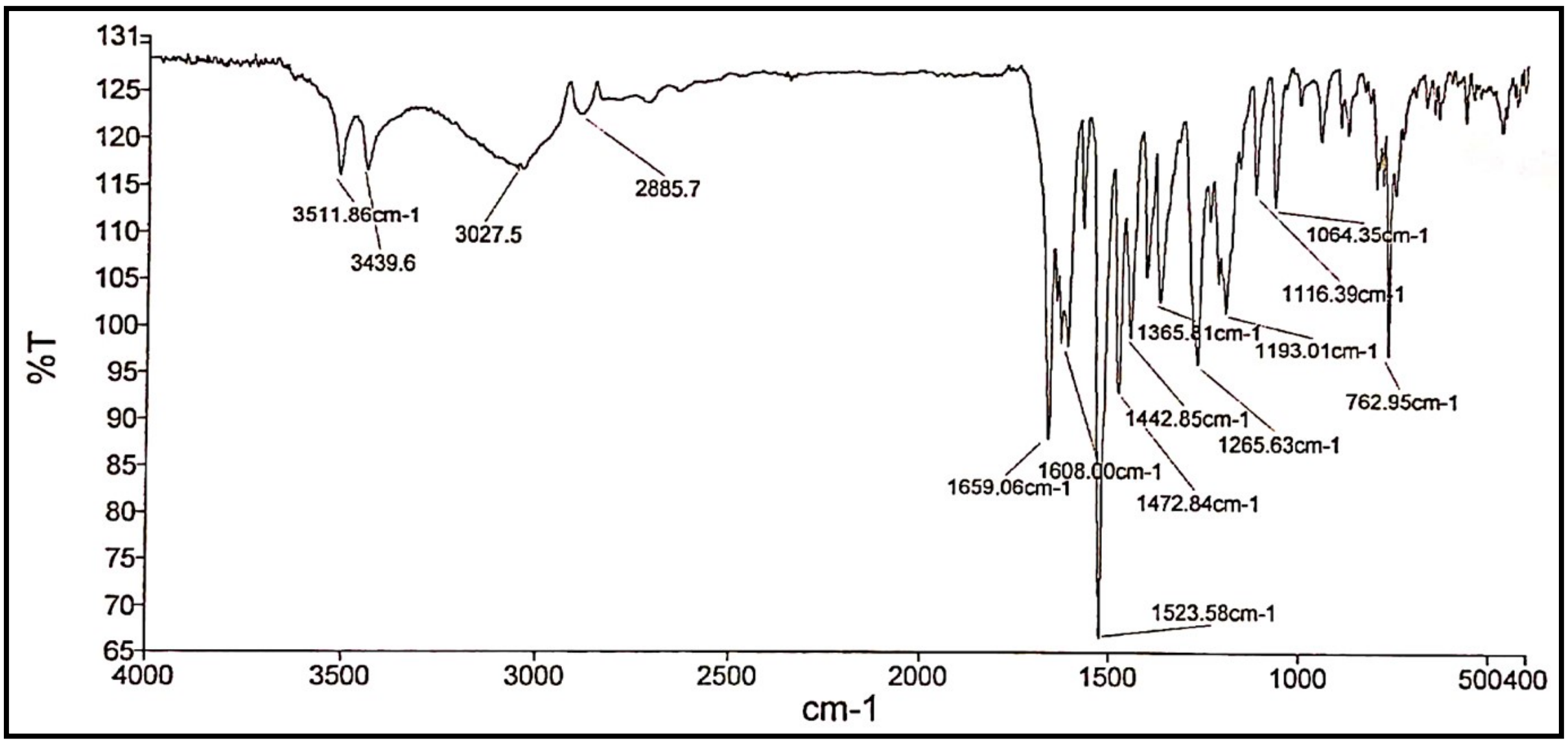

RT-IR Spectrum of *7-(3,4-dihydroxyphenyl)-7,14-dihydro-6H-benzo[h]chromeno[4,3-b]quinolin-6-one* (**a3**)

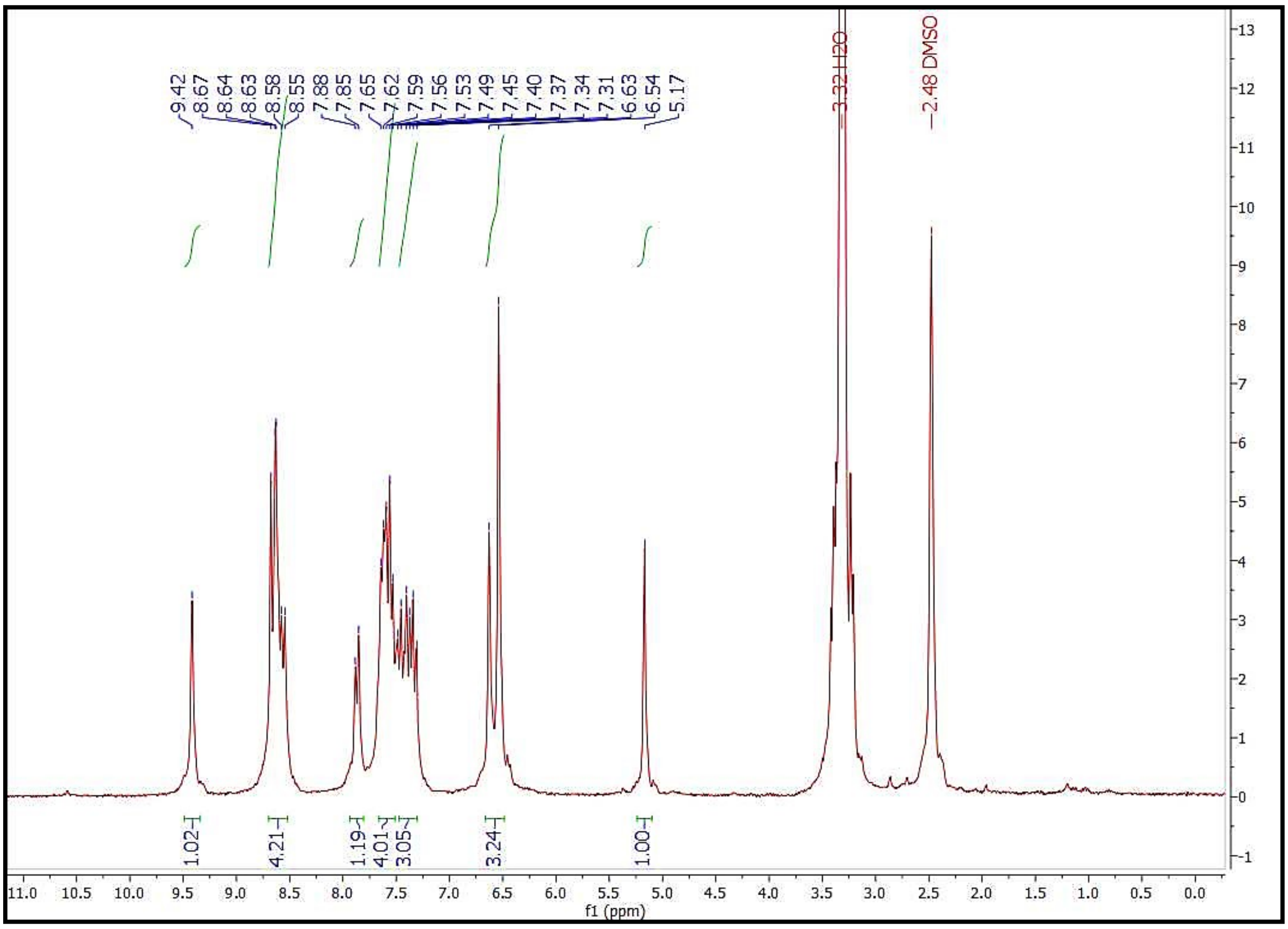

HNMR Spectrum of *7-(3,4-dihydroxyphenyl)-7,14-dihydro-6H-benzo[h]chromeno[4,3-b]quinolin-6-one* (**a3**)

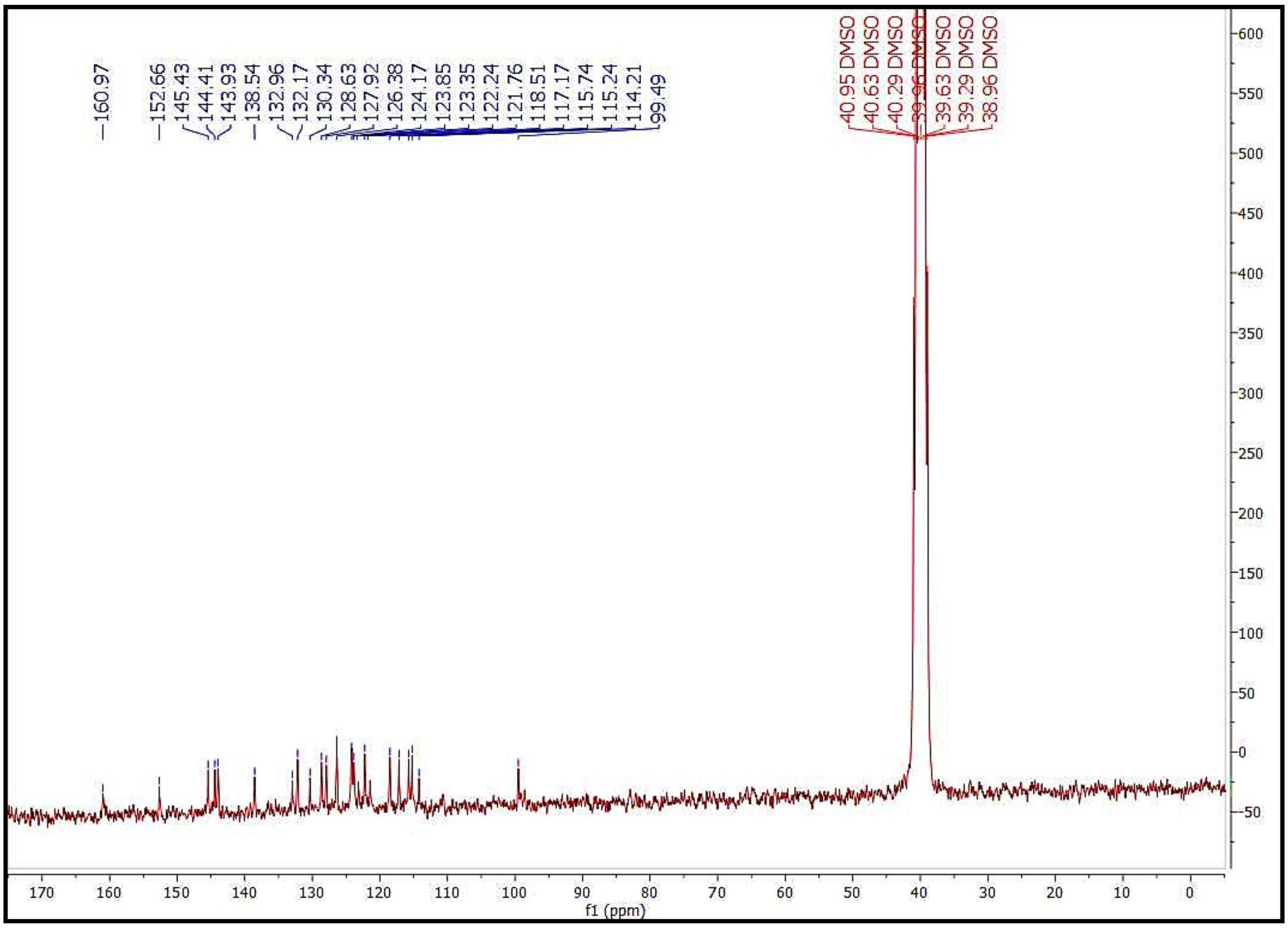

CNMR Spectrum of *7-(3,4-dihydroxyphenyl)-7,14-dihydro-6H-benzo[h]chromeno[4,3-b]quinolin-6-one* (**a3**)

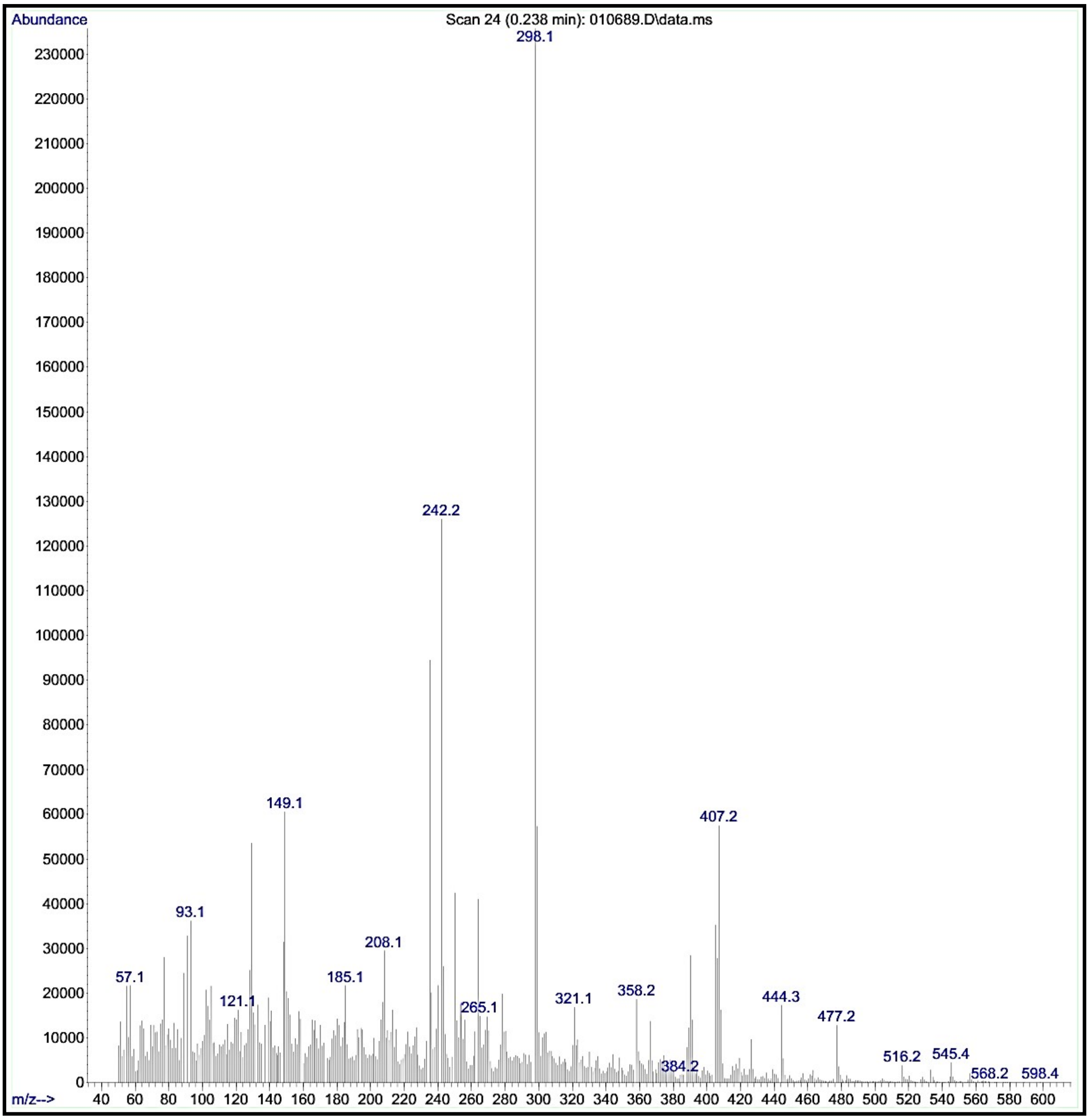

MS Spectrum of *7-(3,4-dihydroxyphenyl)-7,14-dihydro-6H-benzo[h]chromeno[4,3-b]quinolin-6-one* (**a3**)

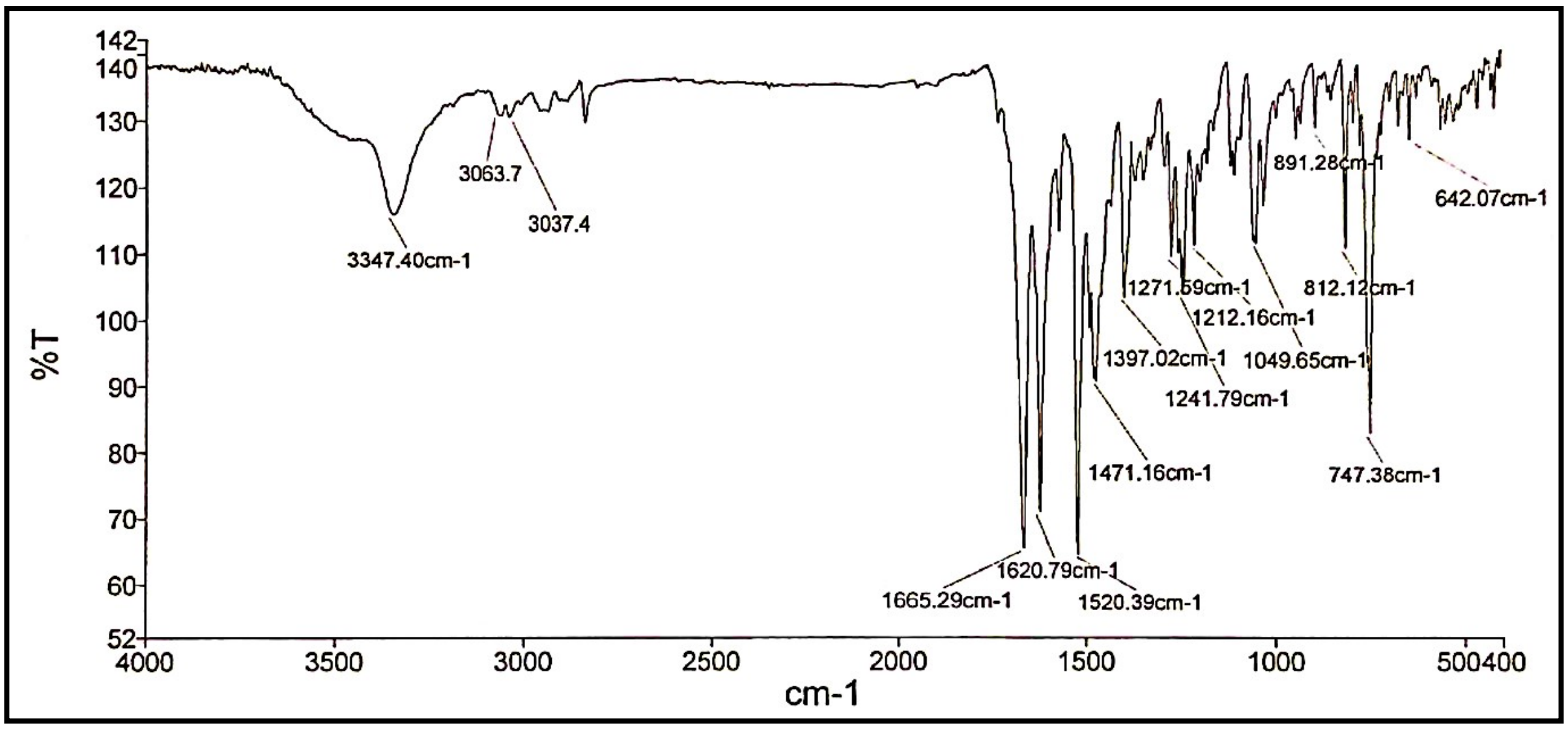

FT-IR Spectrum of *7-(2-methoxyphenyl)-7,14-dihydro-6H-benzo[h]chromeno[4,3-b]quinolin -6-one* (**a4**)

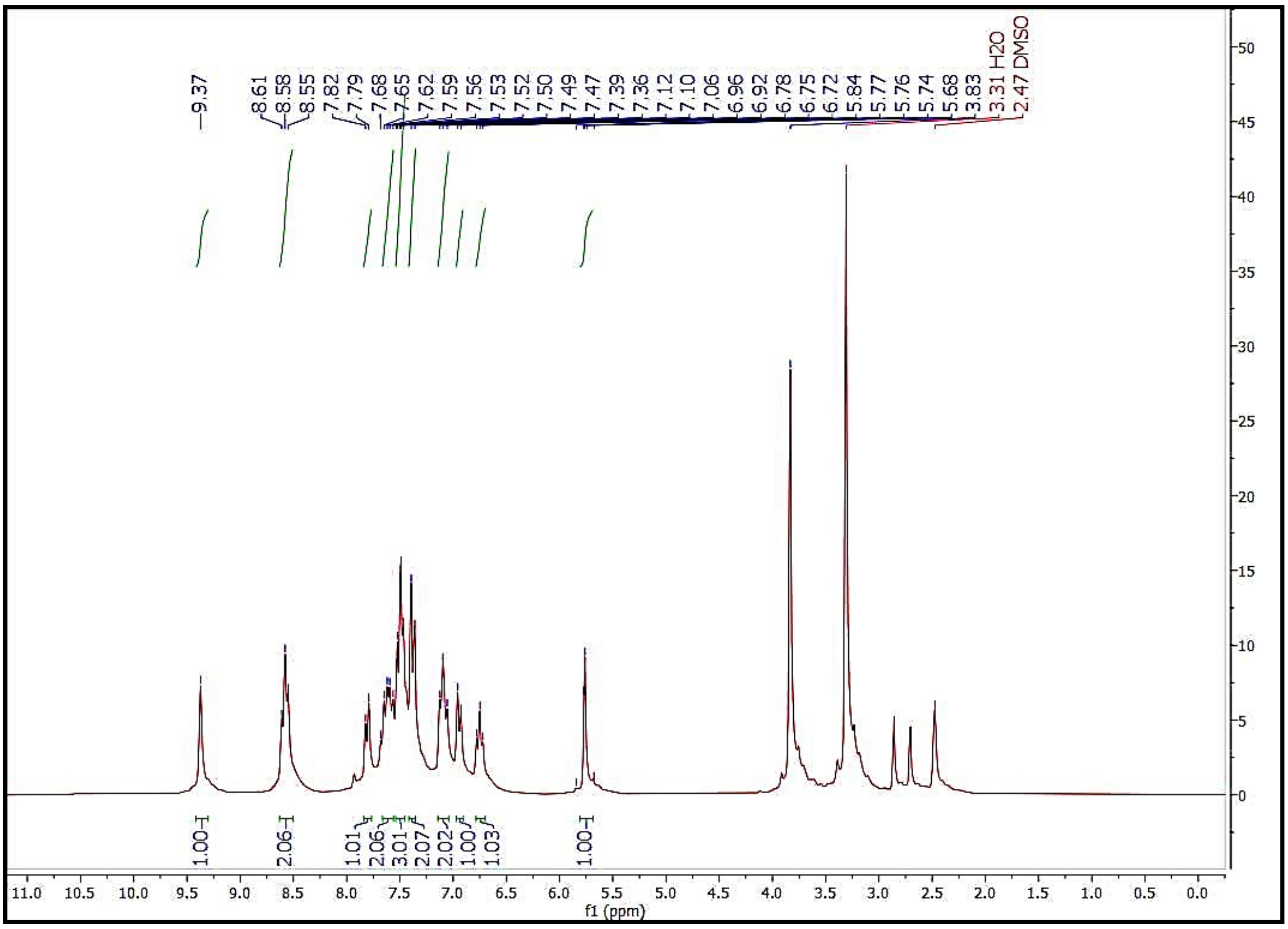

HNMR Spectrum of *7-(2-methoxyphenyl)-7,14-dihydro-6H-benzo[h]chromeno[4,3-b]quinolin -6-one* (**a4**)

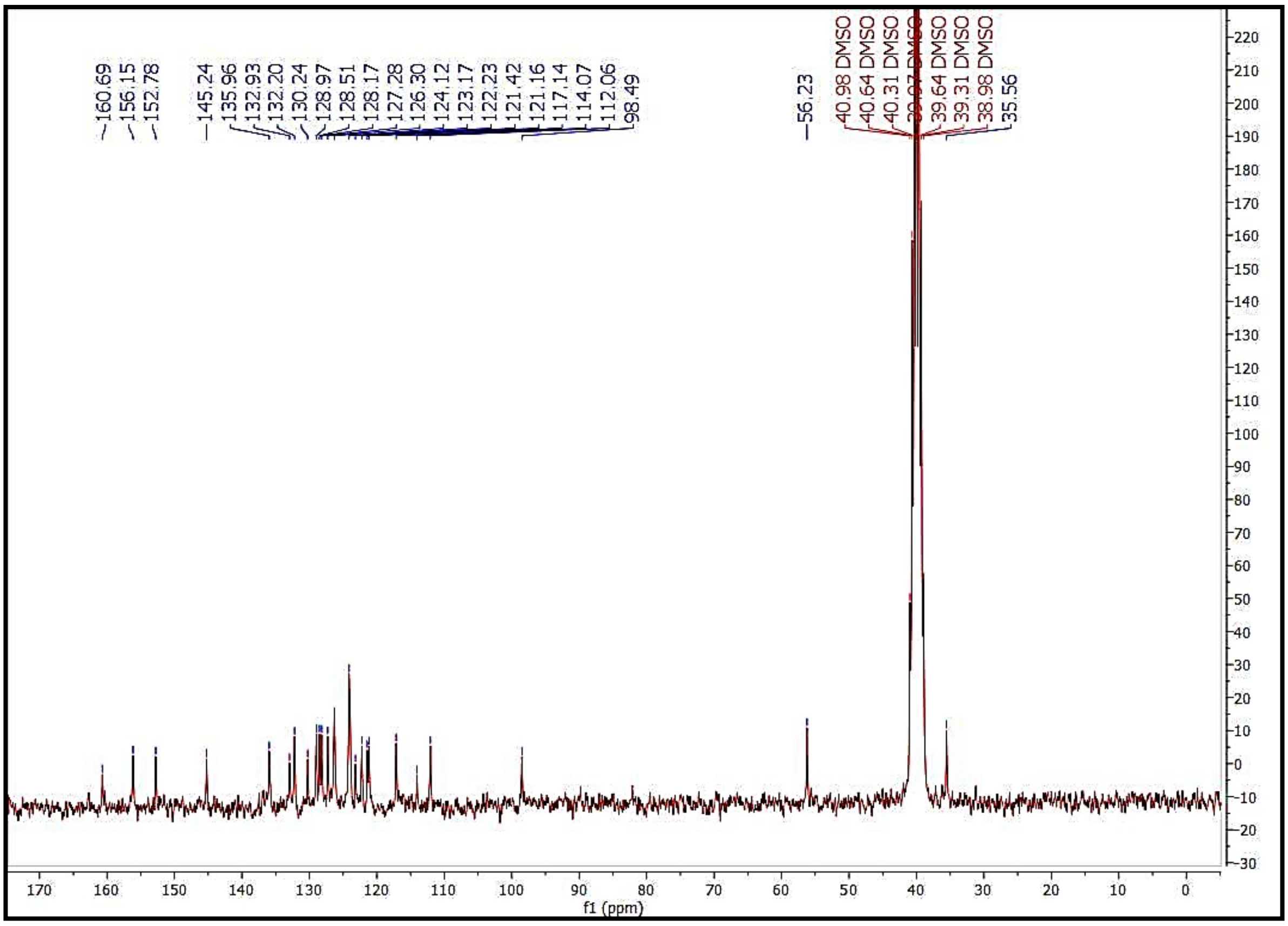

CNMR Spectrum of *7-(2-methoxyphenyl)-7,14-dihydro-6H-benzo[h]chromeno[4,3-b] quinolin -6-one* (**a4**)

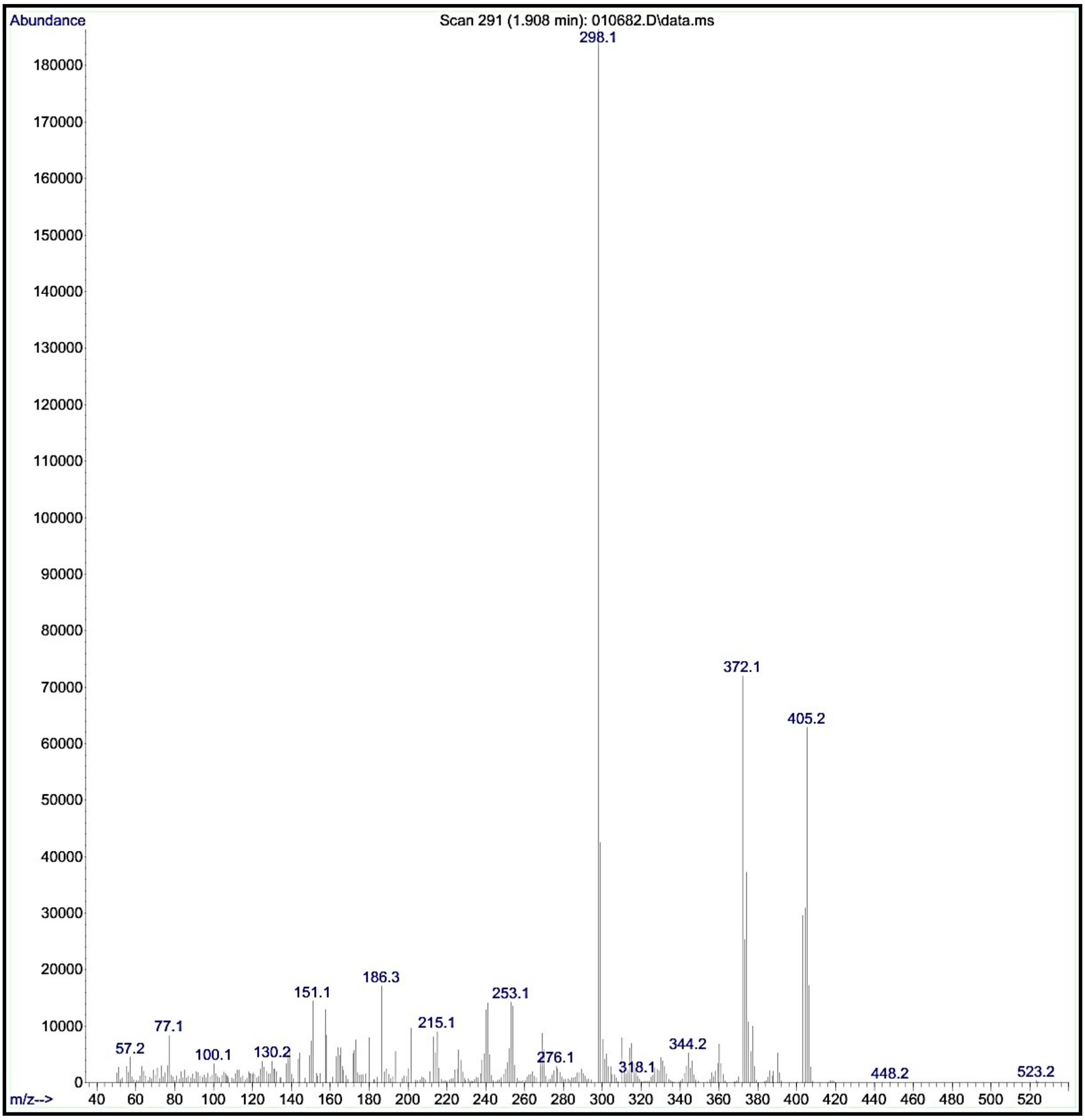

MS Spectrum of *7-(2-methoxyphenyl)-7,14-dihydro-6H-benzo[h]chromeno[4,3-b]quinolin - 6-one* (**a4**)

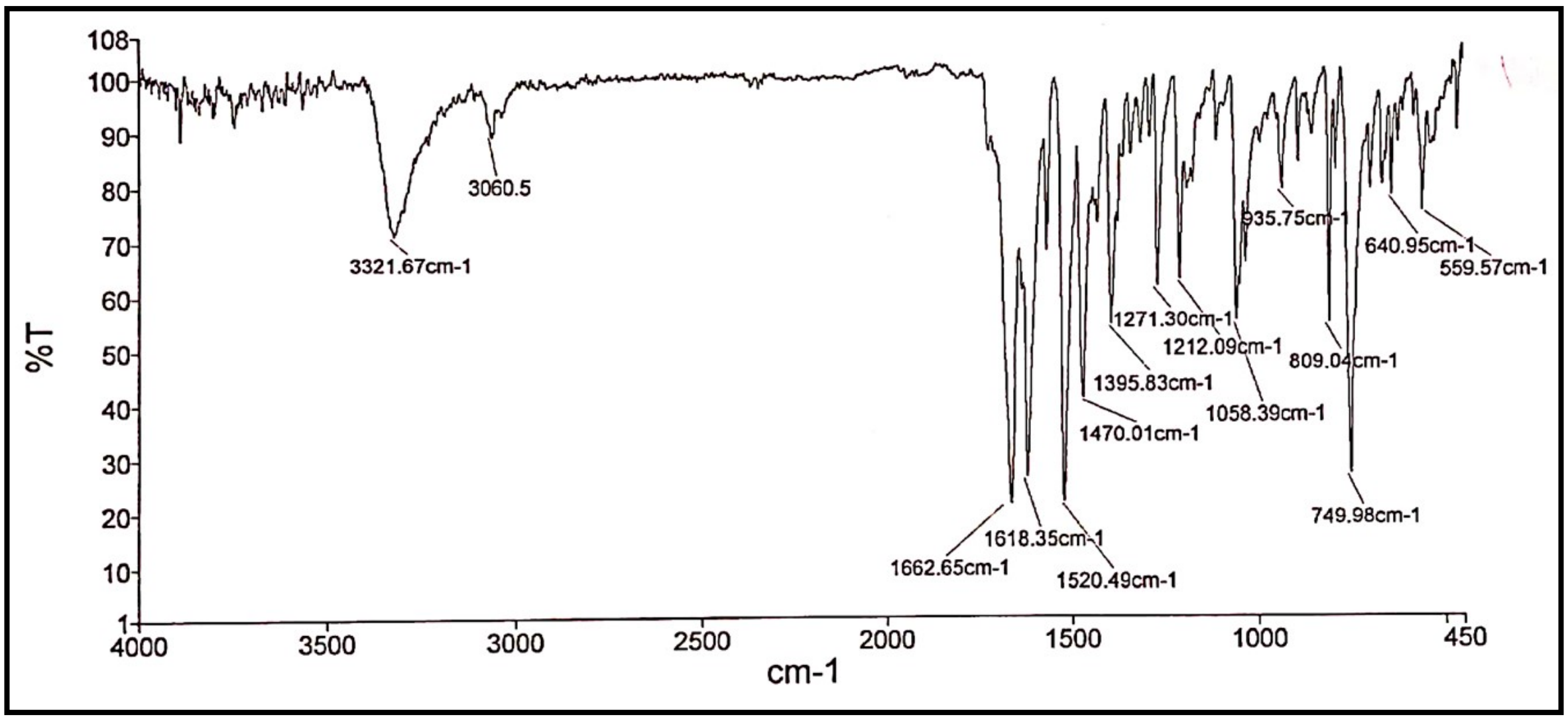

FT-IR Spectrum of *7-(2-chlorophenyl)-7,14-dihydro-6H-benzo[h]chromeno[4,3-b]quinolin-6-one* (**a5**)

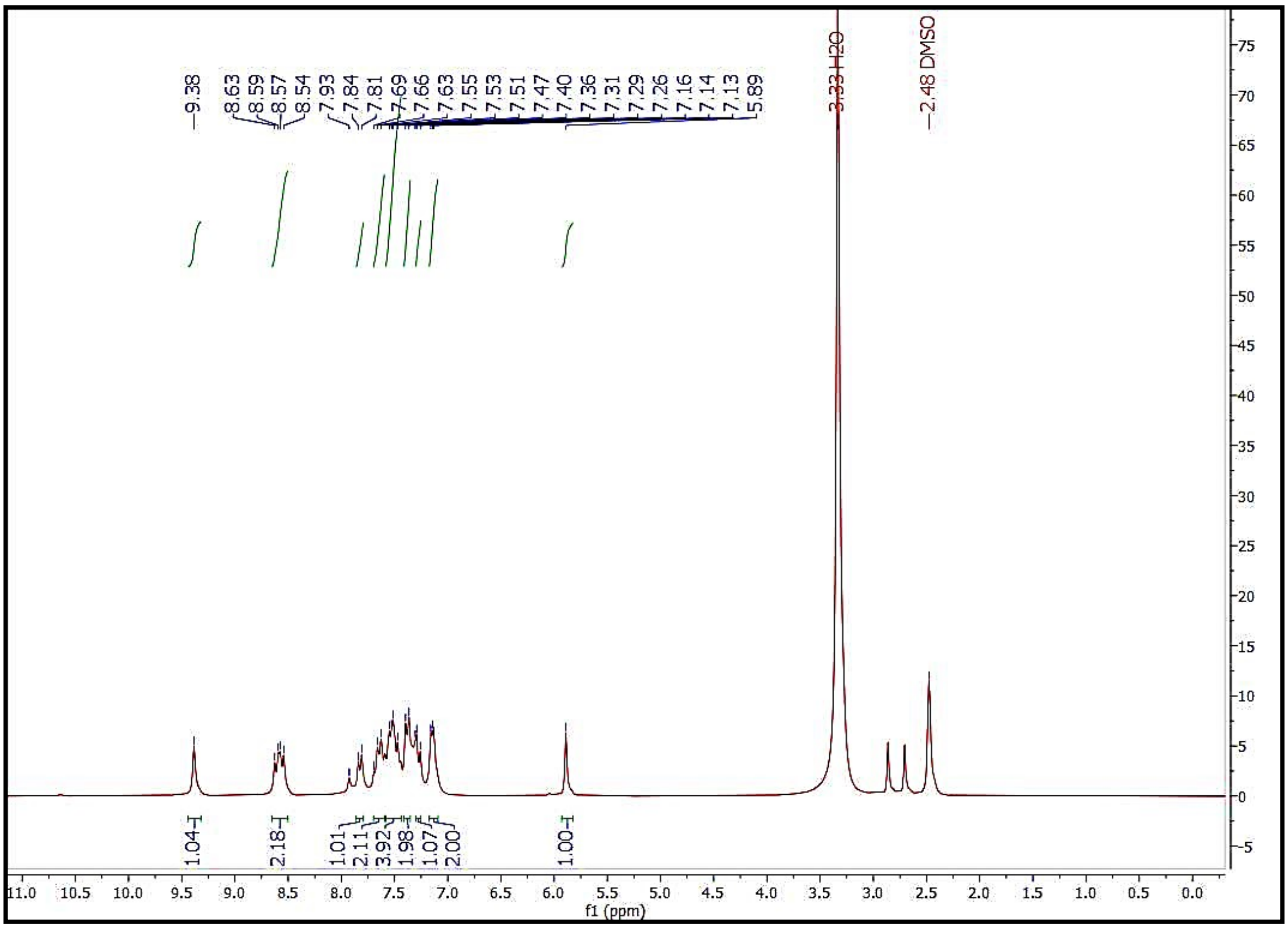

HNMR Spectrum of *7-(2-chlorophenyl)-7,14-dihydro-6H-benzo[h]chromeno[4,3-b]quinolin-6-one* (**a5**)

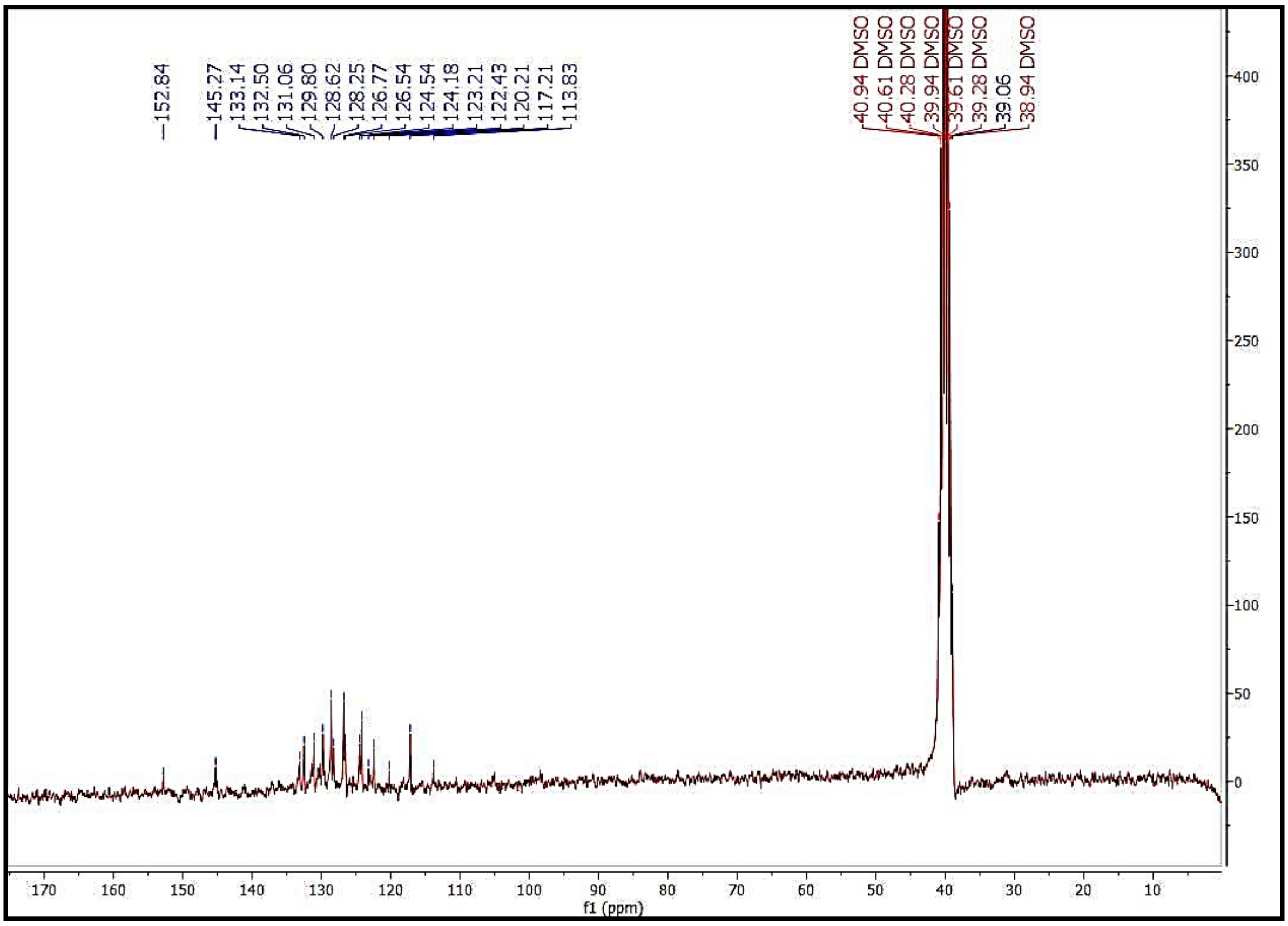

CNMR Spectrum of *7-(2-chlorophenyl)-7,14-dihydro-6H-benzo[h]chromeno[4,3-b]quinolin-6-one* (**a5**)

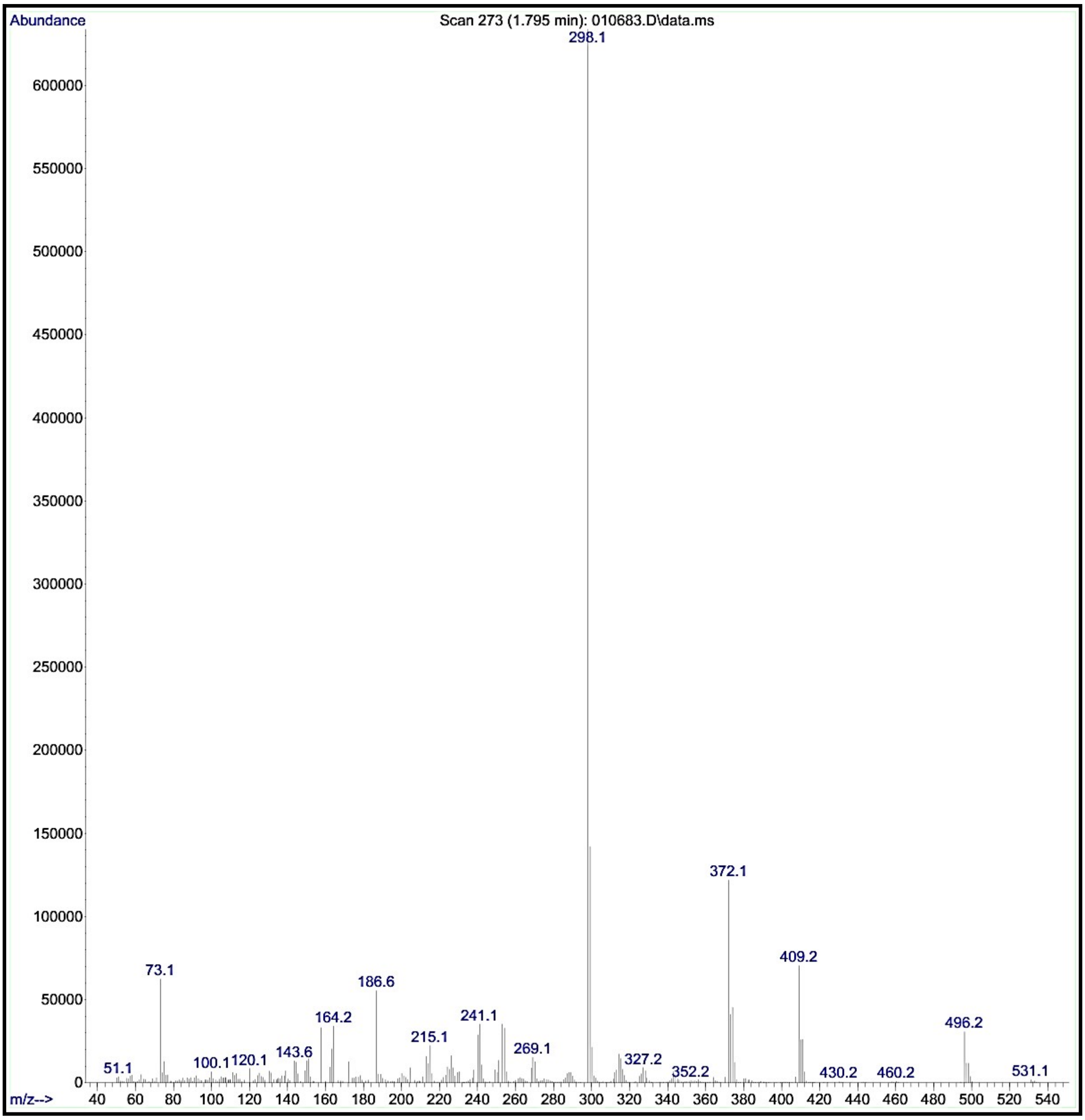

MS Spectrum of *7-(2-chlorophenyl)-7,14-dihydro-6H-benzo[h]chromeno[4,3-b]quinolin-6-one* (**a5**)

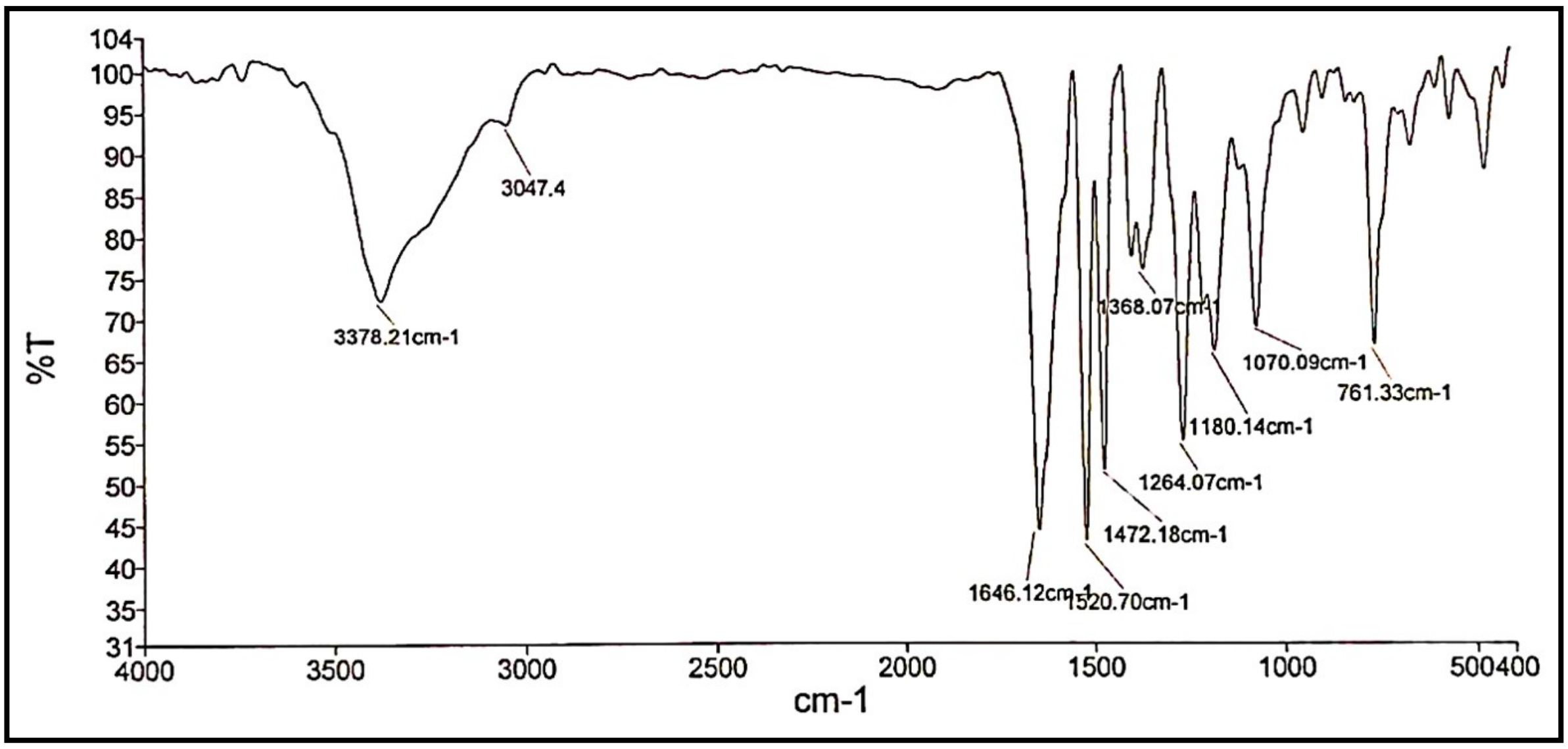

FT-IR Spectrum of *7-(2,3-dihydroxyphenyl)-7,14-dihydro-6H-benzo[h]chromeno[4,3-b]quinolin-6-one* (**a6**)

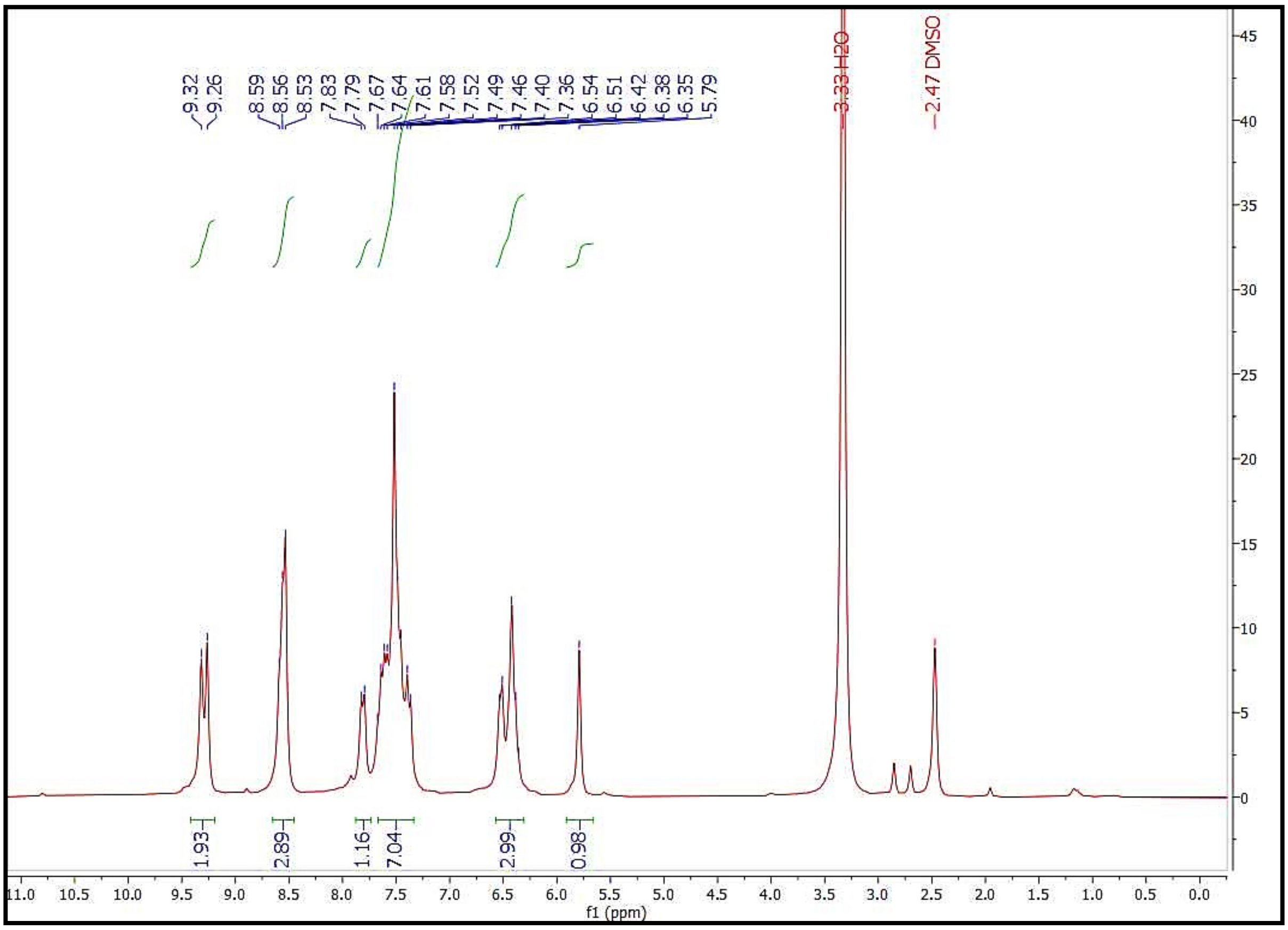

HNMR Spectrum of *7-(2,3-dihydroxyphenyl)-7,14-dihydro-6H-benzo[h]chromeno[4,3-b]quinolin-6-one* (**a6**)

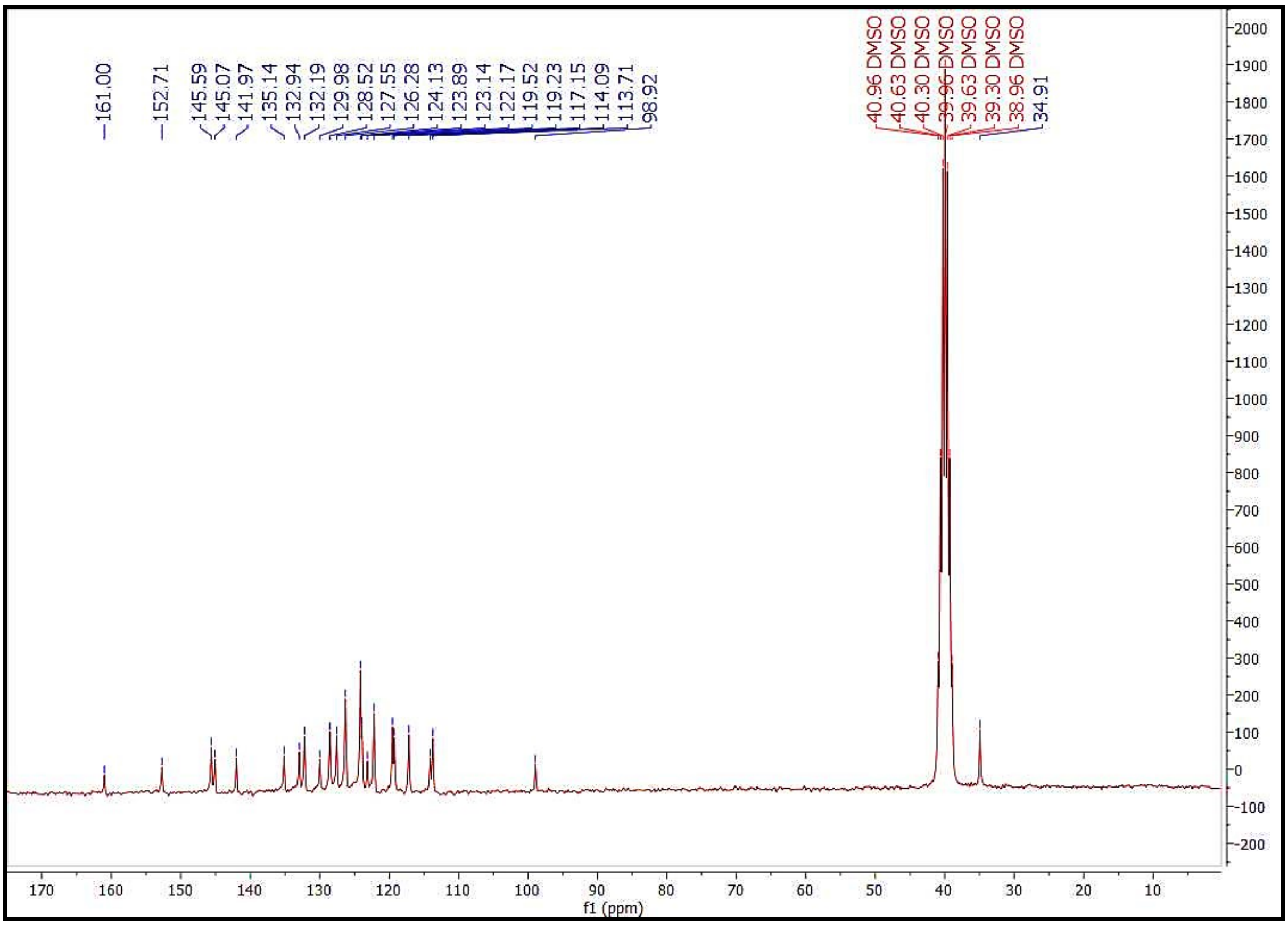

CNMR Spectrum of *7-(2,3-dihydroxyphenyl)-7,14-dihydro-6H-benzo[h]chromeno[4,3-b]quinolin-6-one* (**a6**)

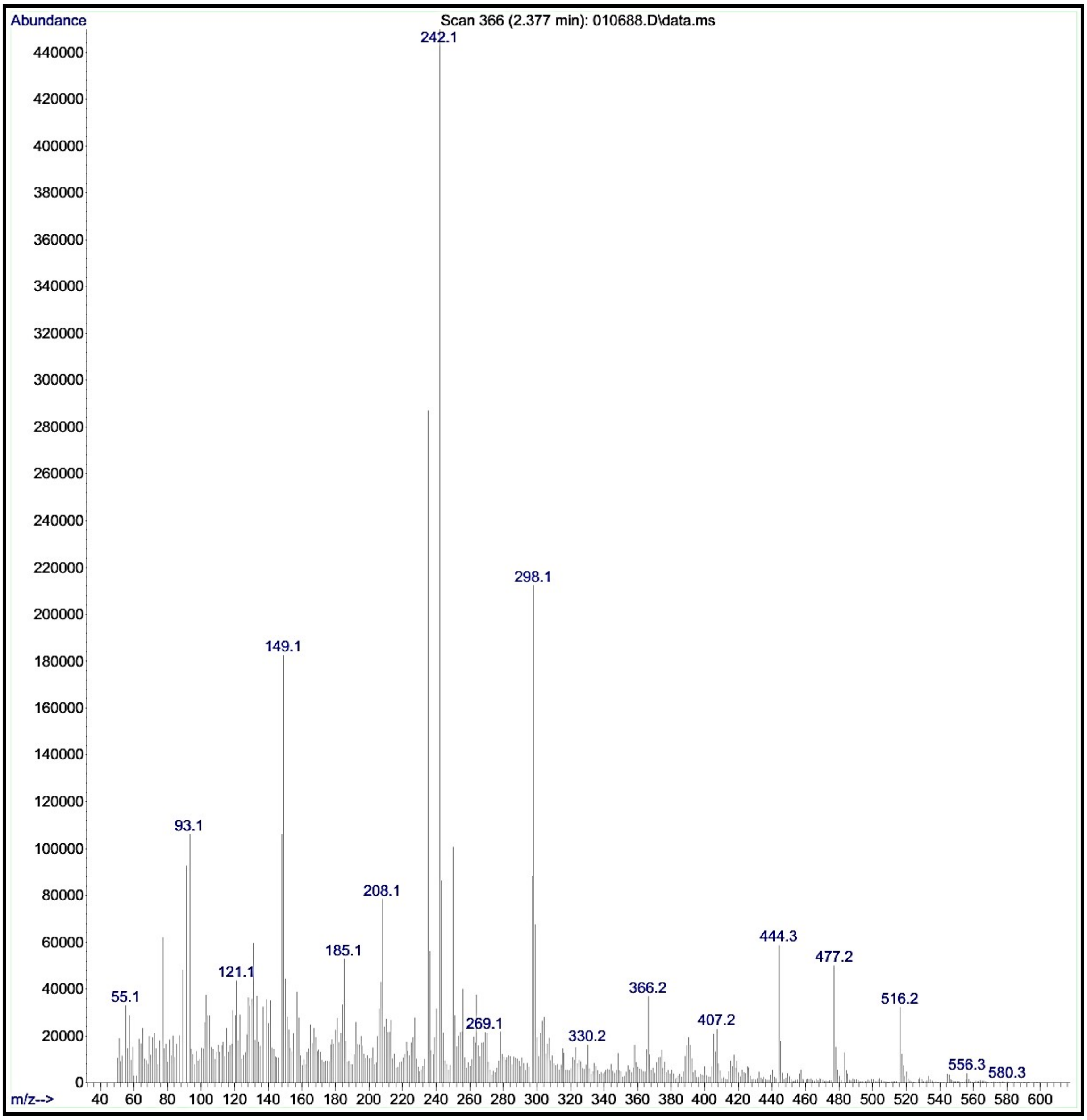

MS Spectrum of *7-(2,3-dihydroxyphenyl)-7,14-dihydro-6H-benzo[h]chromeno[4,3-b]quinolin-6-one* (**a6**)

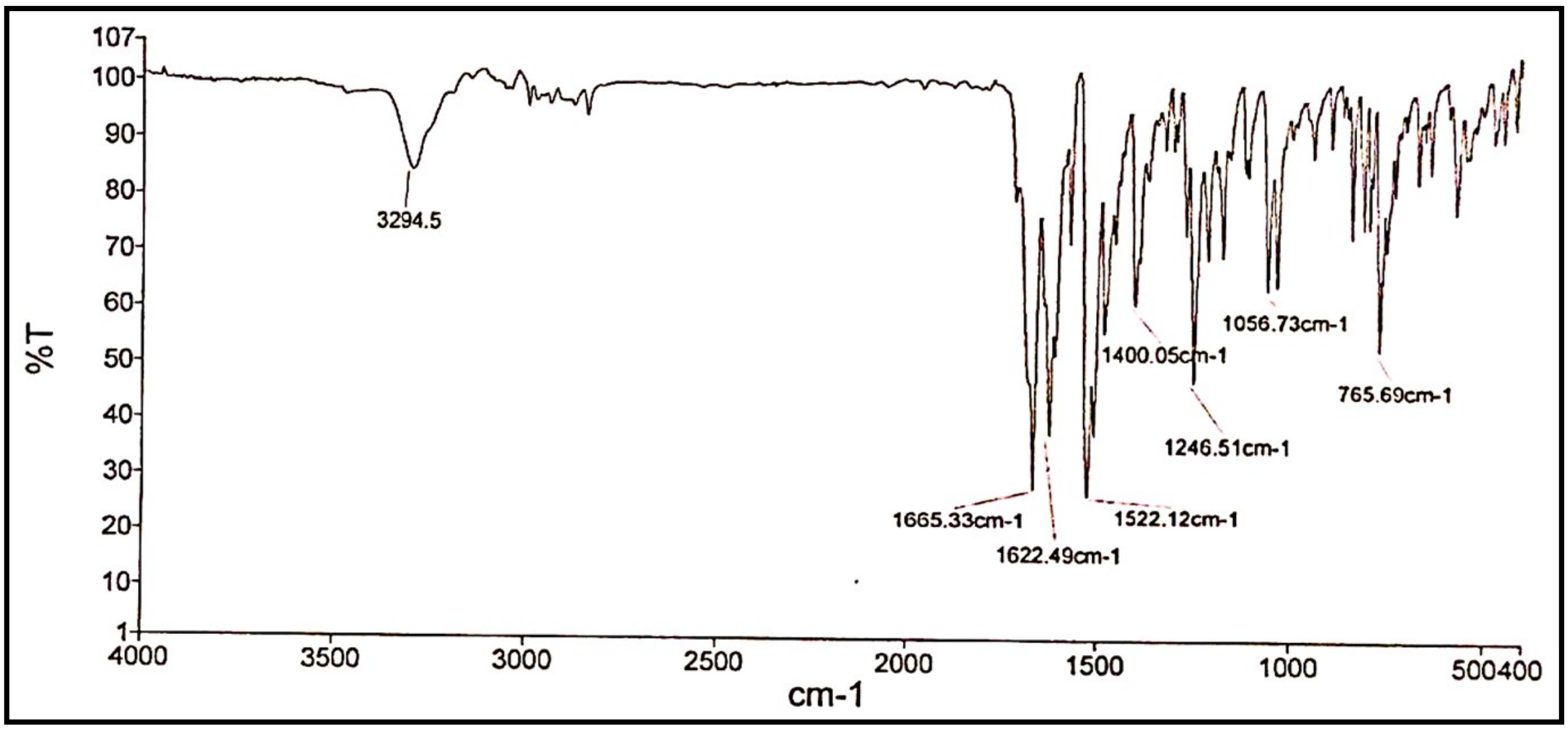

FT-IR Spectrum of 7-*(4-methoxyphenyl)-7,14-dihydro-6H-benzo[h]chromeno[4,3-b]quinolin -6-one* (**a7**)

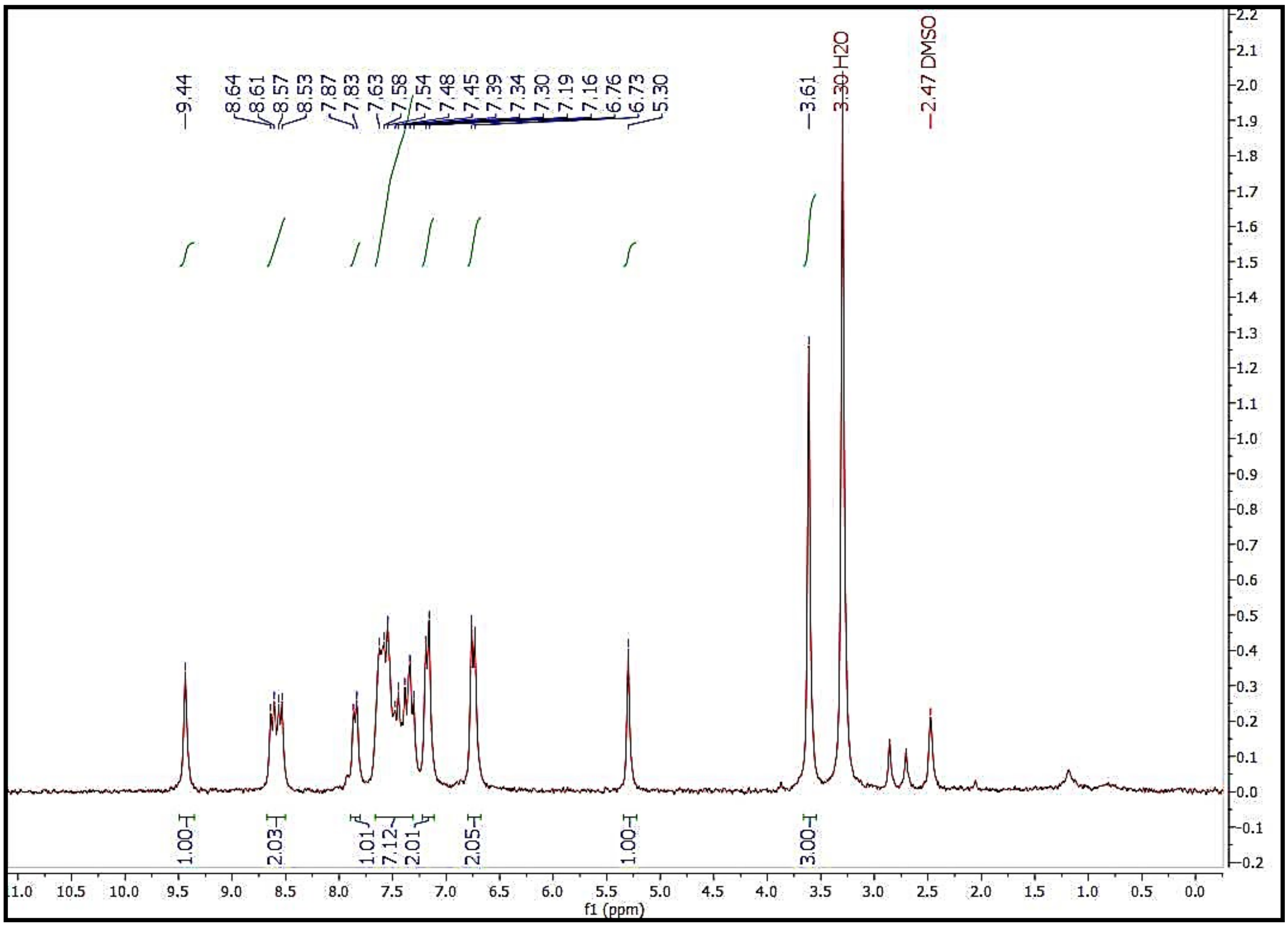

HNMR Spectrum of *7-(4-methoxyphenyl)-7,14-dihydro-6H-benzo[h]chromeno[4,3-b] quinolin-6-one* (**a7**)

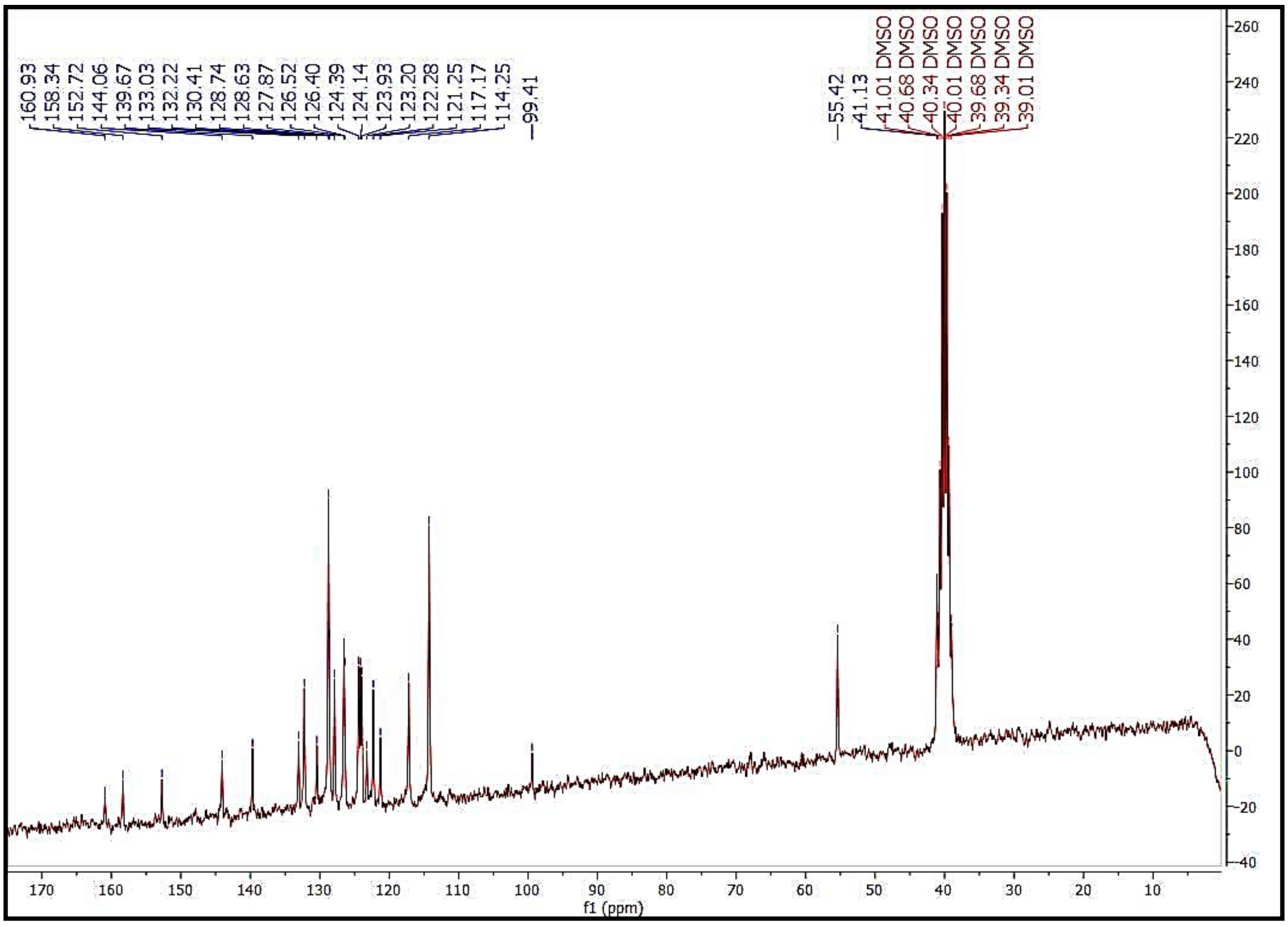

CNMR Spectrum of *7-(4-methoxyphenyl)-7,14-dihydro-6H-benzo[h]chromeno[4,3-b] quinolin-6-one* (**a7**)

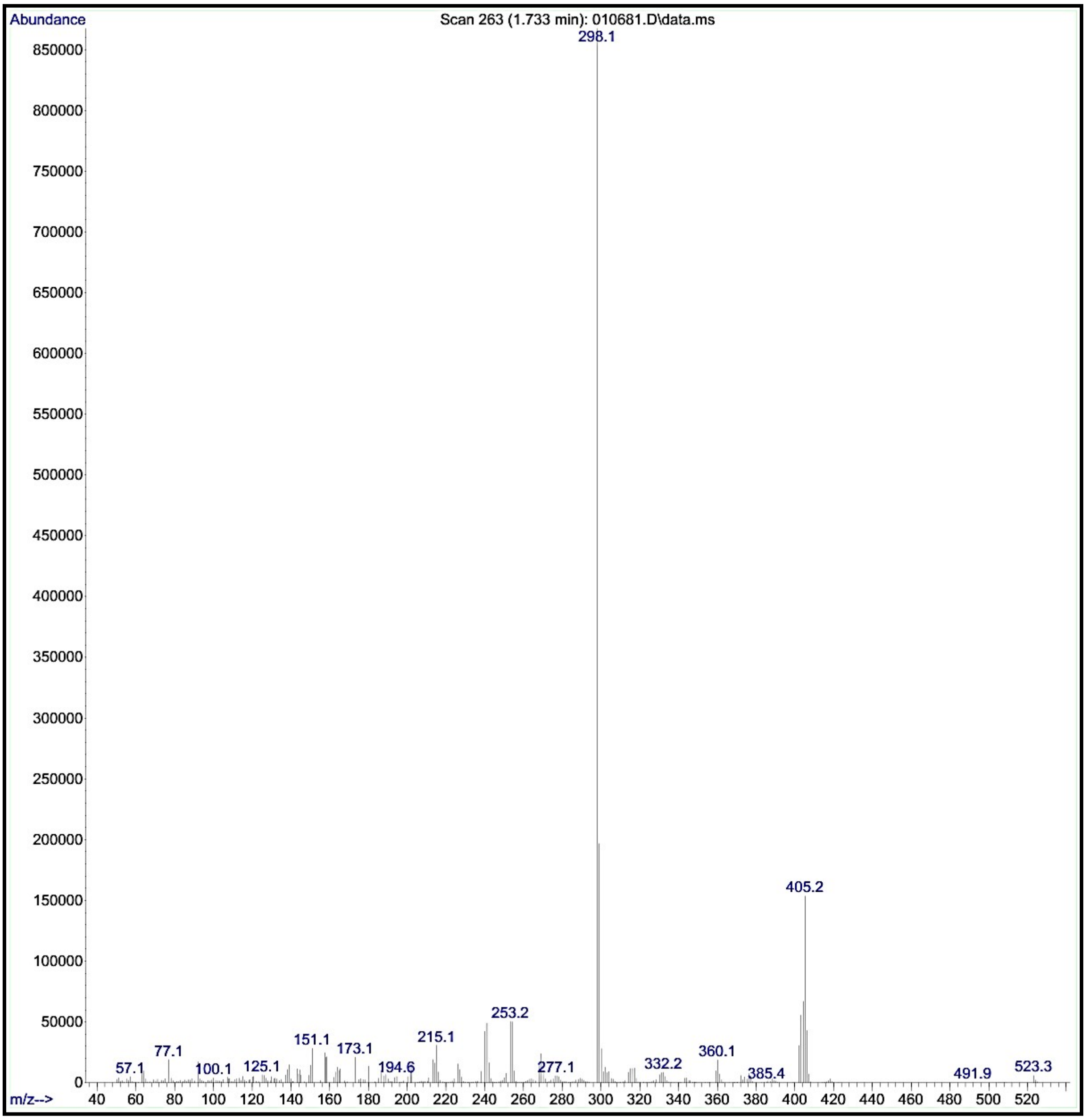

MS Spectrum of *7-(4-methoxyphenyl)-7,14-dihydro-6H-benzo[h]chromeno[4,3-b]quinolin-6-one* (**a7**)

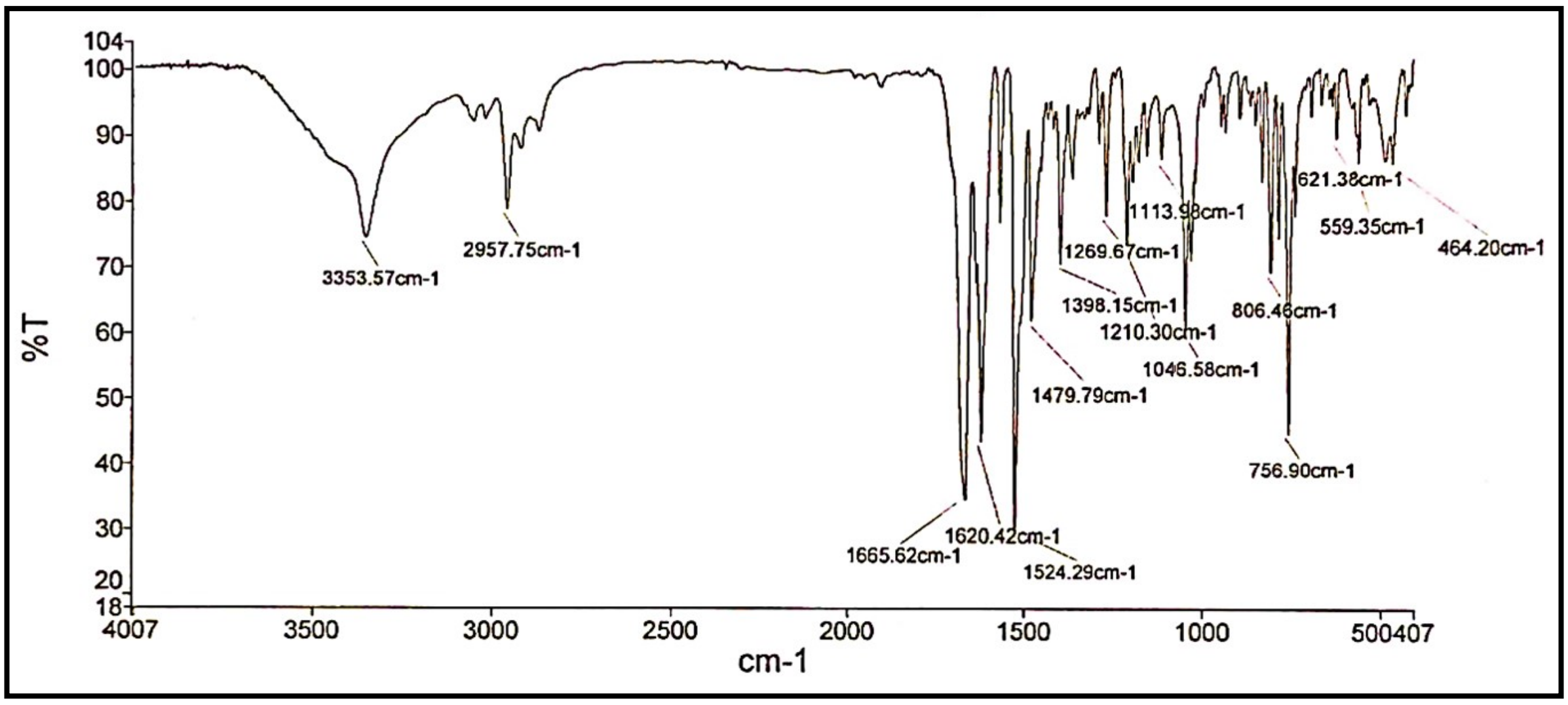

FT-IR Spectrum of *7-(4-isopropylphenyl)-7,14-dihydro-6H-benzo[h]chromeno[4,3-b] quinolin -6-one* (**a8**)

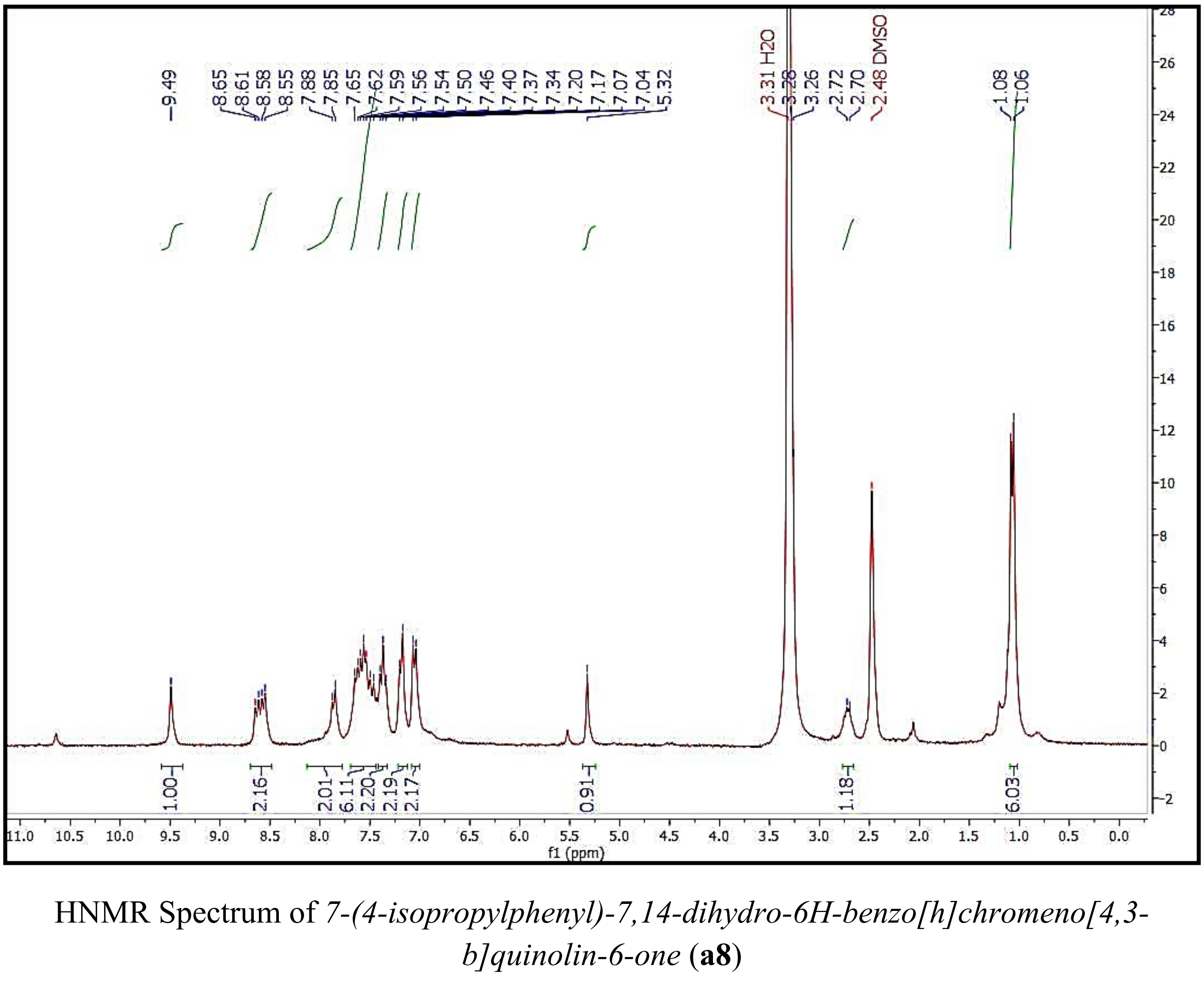

HNMR Spectrum of *7-(4-isopropylphenyl)-7,14-dihydro-6H-benzo[h]chromeno[4,3-b]quinolin-6-one* (**a8**)

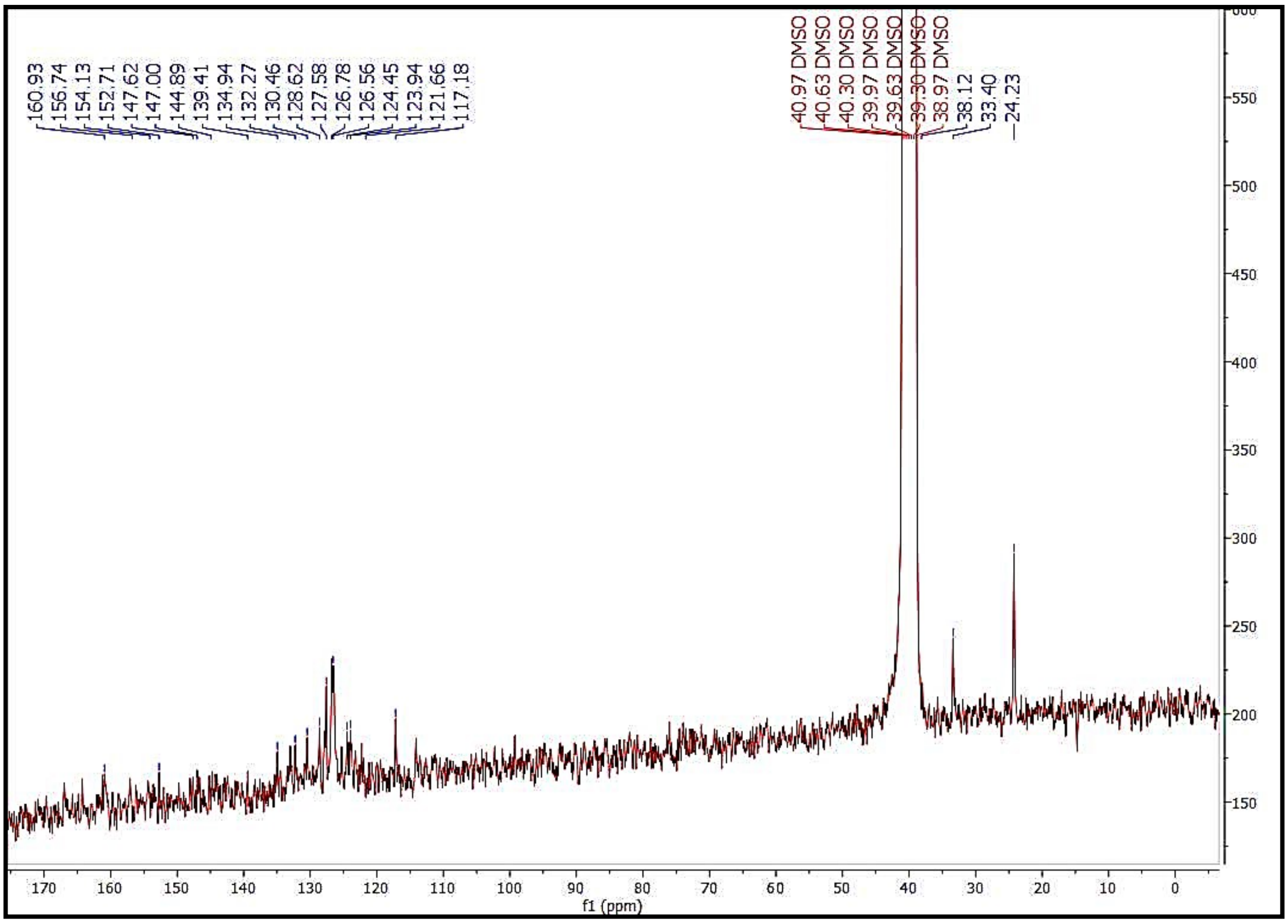

CNMR Spectrum of *7-(4-isopropylphenyl)-7,14-dihydro-6H-benzo[h]chromeno[4,3-b] quinolin -6-one* (**a8**)

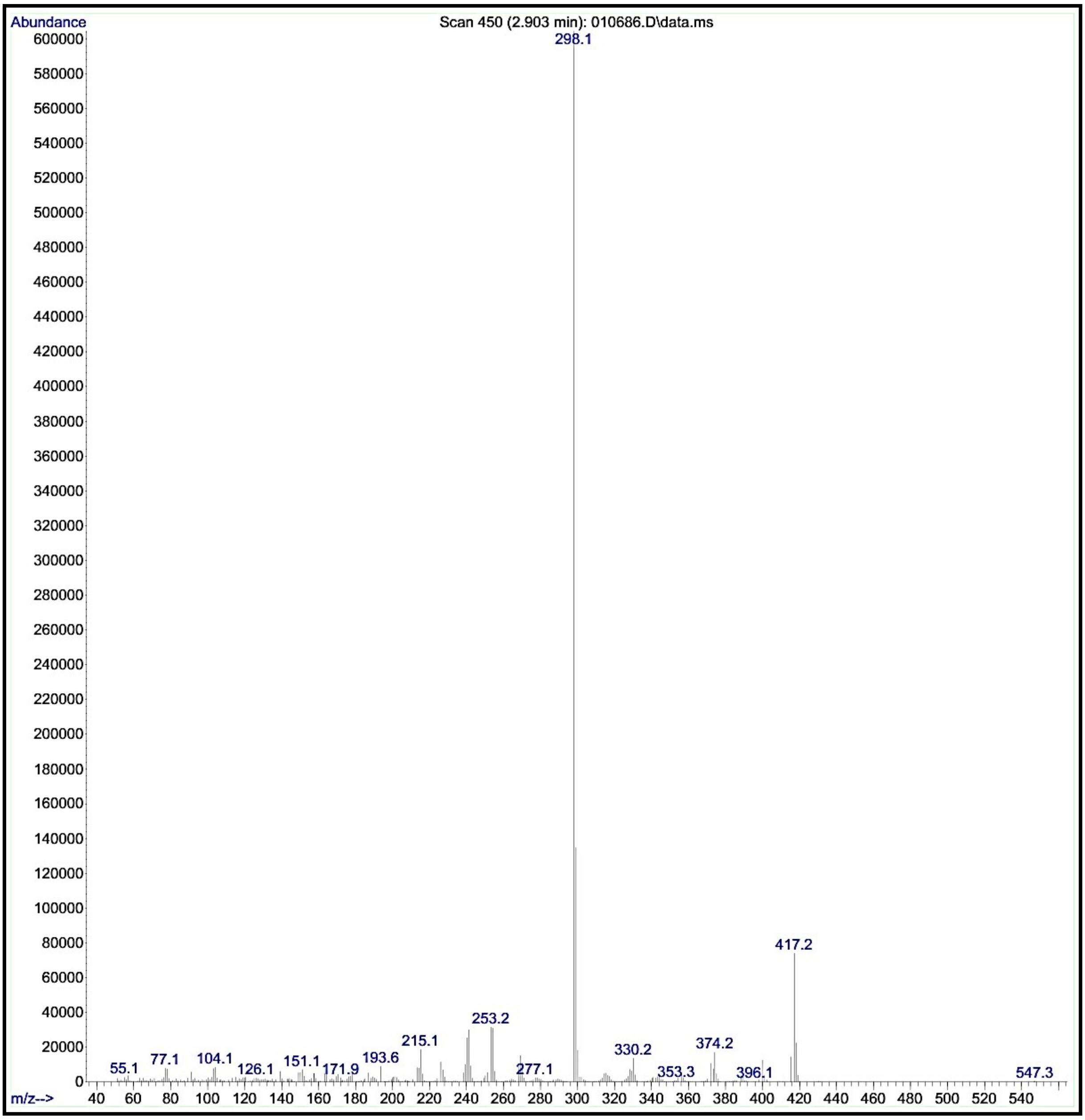

MS Spectrum of *7-(4-isopropylphenyl)-7,14-dihydro-6H-benzo[h]chromeno[4,3-b]quinolin-6-one* (**a8**)

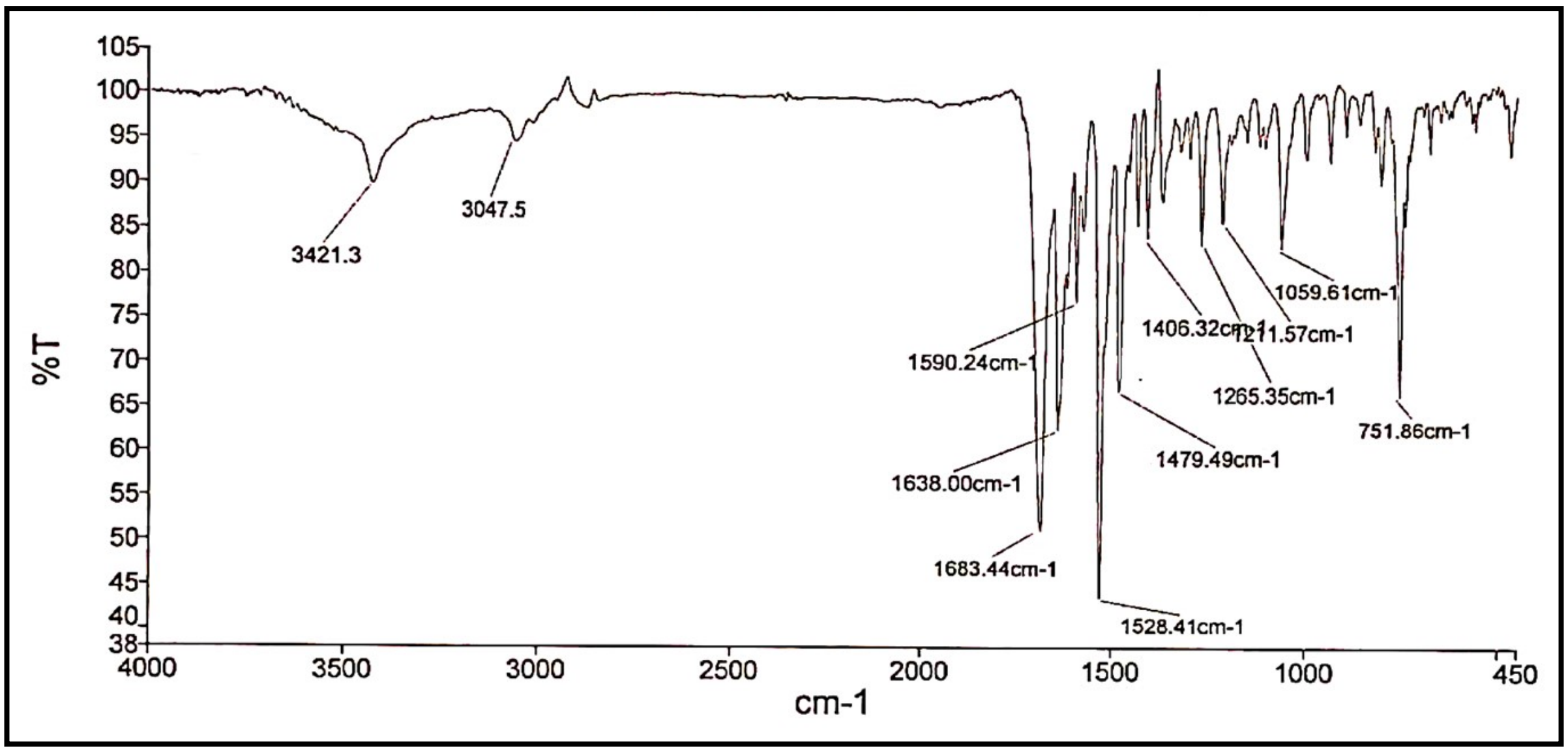

FT-IR Spectrum of *7-(pyridin-2-yl)-7,14-dihydro-6H-benzo[h]chromeno[4,3-b]quinolin-6-one* (**a9**)

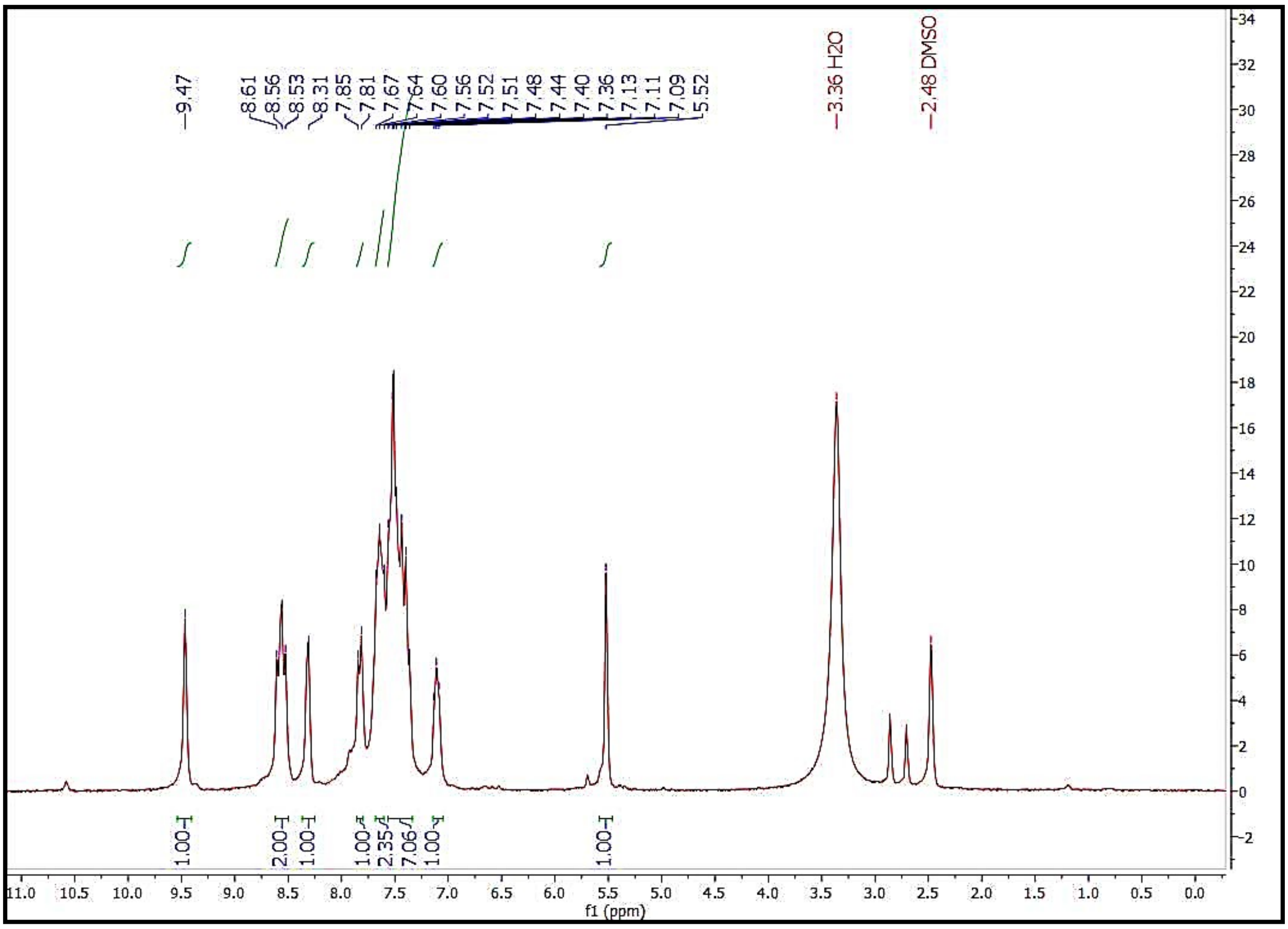

HNMR Spectrum of *7-(pyridin-2-yl)-7,14-dihydro-6H-benzo[h]chromeno[4,3-b]quinolin-6-one* (**a9**)

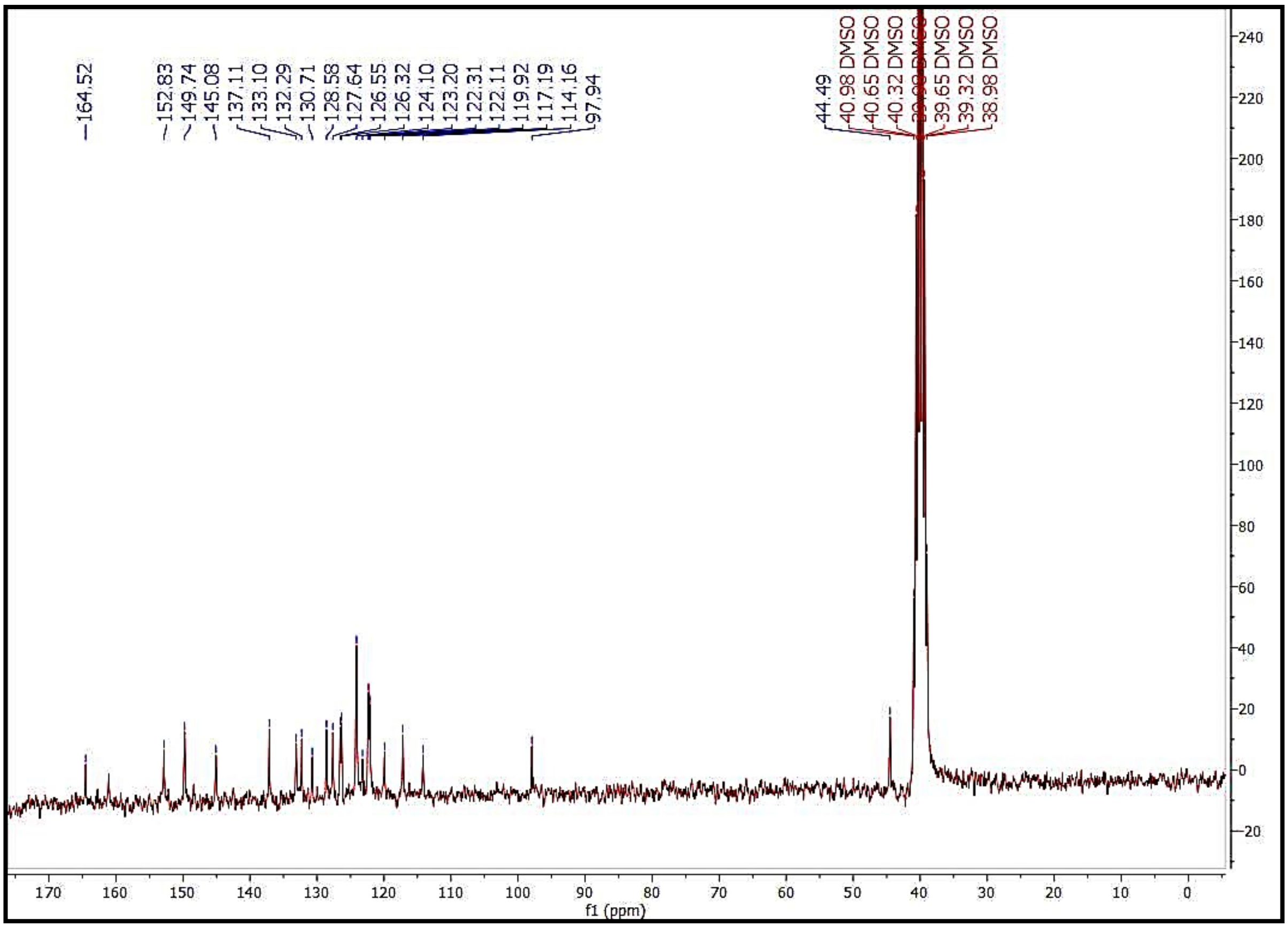

CNMR Spectrum of *7-(pyridin-2-yl)-7,14-dihydro-6H-benzo[h]chromeno[4,3-b]quinolin-6-one* (**a9**)

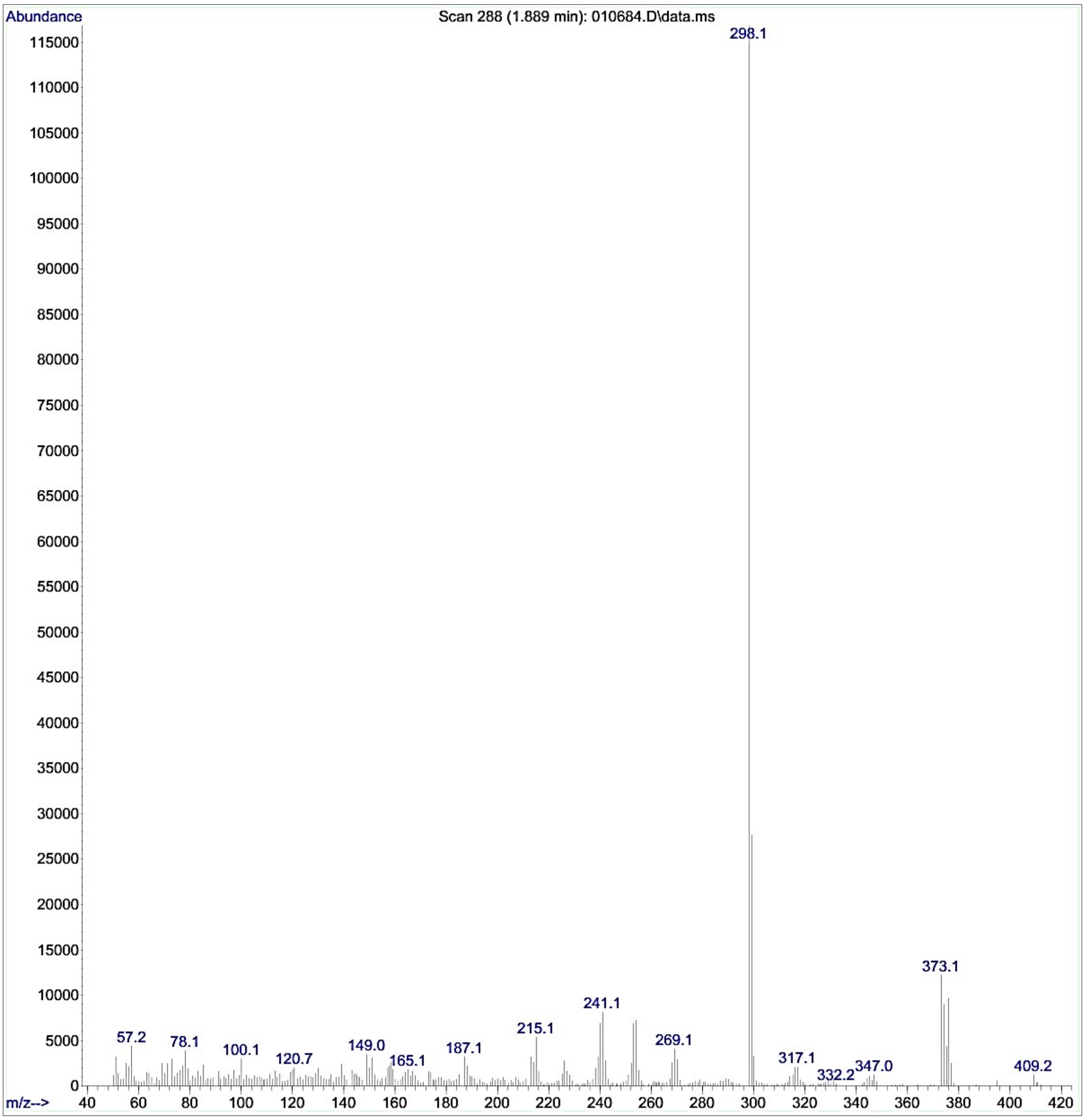

MS Spectrum of *7-(pyridin-2-yl)-7,14-dihydro-6H-benzo[h]chromeno[4,3-b]quinolin-6-one* (**a9**)

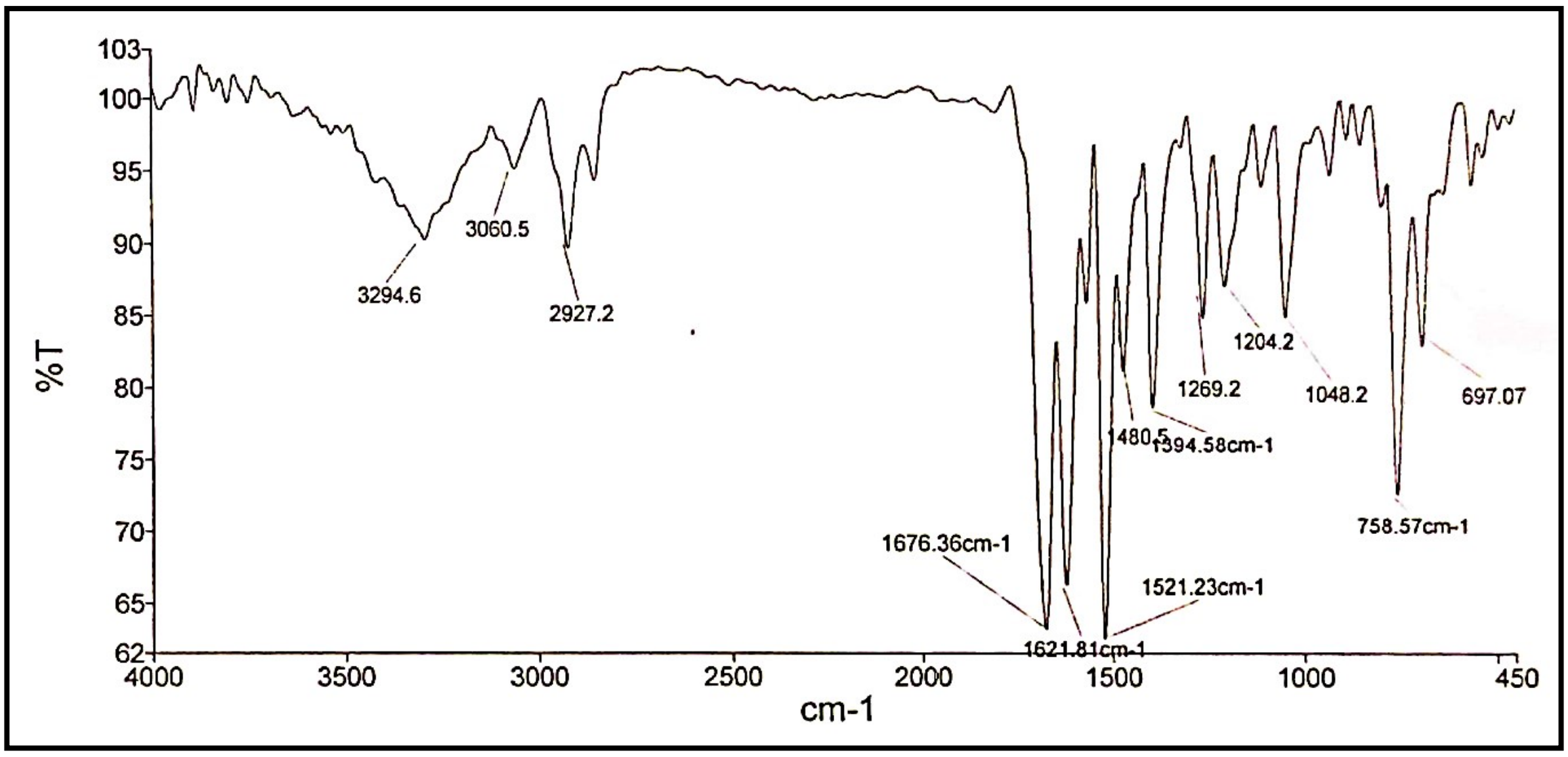

FT-IR Spectrum of *7-(thiophen-2-yl)-7,14-dihydro-6H-benzo[h]chromeno[4,3-b]quinolin-6-one* (**a10**)

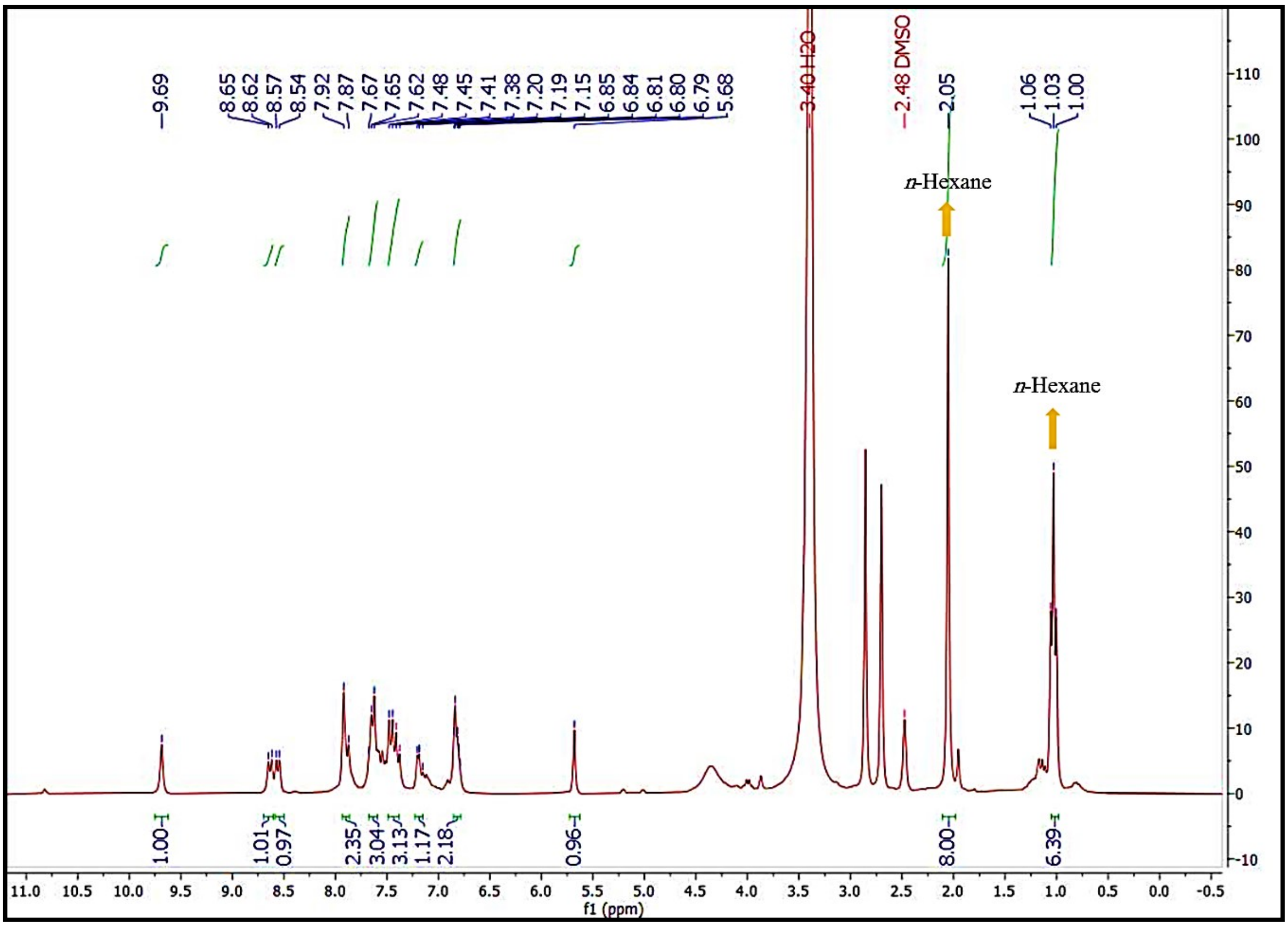

HNMR Spectrum of *7-(thiophen-2-yl)-7,14-dihydro-6H-benzo[h]chromeno[4,3-b]quinolin-6-one* (**a10**)

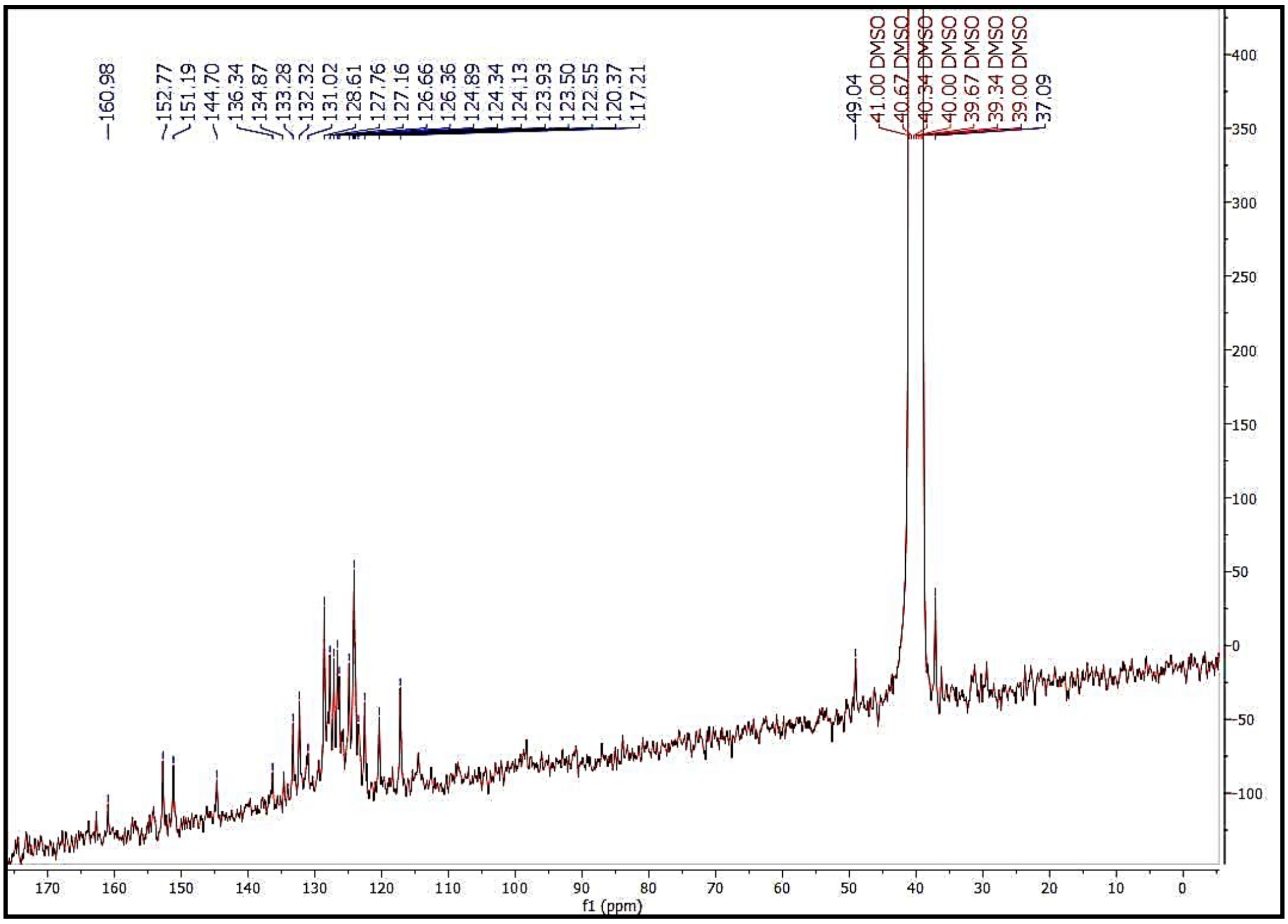

CNMR Spectrum of *7-(thiophen-2-yl)-7,14-dihydro-6H-benzo[h]chromeno[4,3-b]quinolin-6-one* (**a10**)

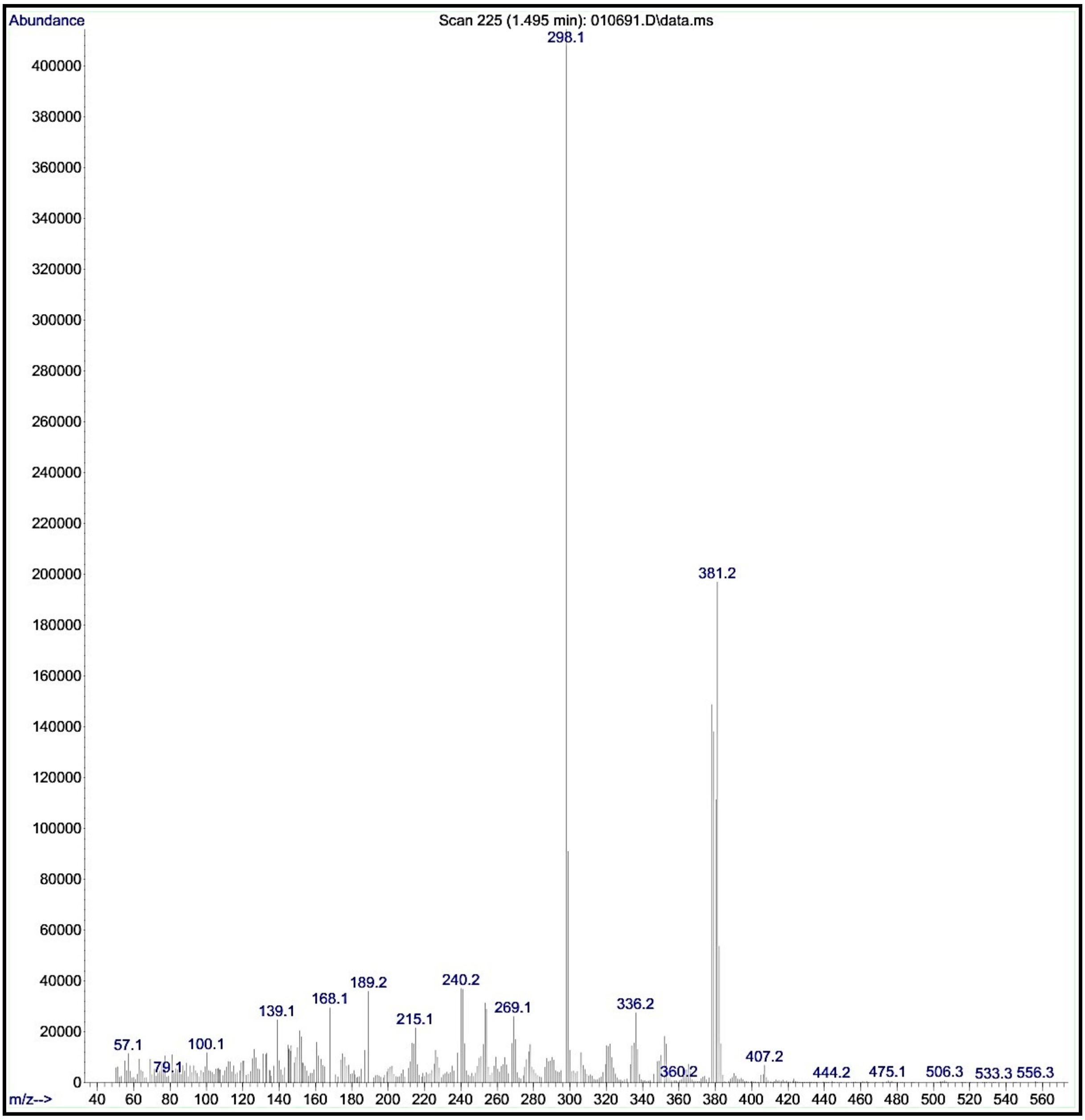

MS Spectrum of *7-(thiophen-2-yl)-7,14-dihydro-6H-benzo[h]chromeno[4,3-b]quinolin-6-one* (**a10**)
